# Chemical Investigation of the Calcareous Marine Sponge *Pericharax heteroraphis*, Clathridine-A Related Derivatives Isolation, Synthesis and Osteogenic Activity

**DOI:** 10.3390/md22050196

**Published:** 2024-04-25

**Authors:** Capucine Jourdain de Muizon, Céline Moriou, Marceau Levasseur, David Touboul, Bogdan I. Iorga, Hristo Nedev, Elsa Van Elslande, Pascal Retailleau, Sylvain Petek, Eric Folcher, Arnaud Bianchi, Mireille Thomas, Solène Viallon, Sylvie Peyroche, Sarah Nahle, Marthe Rousseau, Ali Al-Mourabit

**Affiliations:** 1 CNRS, Institut de Chimie des Substances Naturelles, Université Paris-Saclay, 91190 Gif-sur-Yvette, France; capucine.jourdain@ensc-rennes.fr (C.J.d.M.); celine.moriou@cnrs.fr (C.M.); marceau.levasseur@inrae.fr (M.L.); david.touboul@cnrs.fr (D.T.); bogdan.iorga@cnrs.fr (B.I.I.); hristo.nedev@cnrs.fr (H.N.); elsa.van-elslande@cnrs.fr (E.V.E.); pascal.retailleau@cnrs.fr (P.R.); 2 Laboratoire de Chimie Moléculaire (LCM), CNRS, École Polytechnique, Institut Polytechnique de Paris, 91120 Palaiseau, France; 3 IRD, CNRS, Ifremer, Univ Brest, LEMAR, IUEM, 29280 Plouzane, France; sylvain.petek@ird.fr; 4 IRD, SEOH, BPA5, Nouméa 98848, New Caledonia; eric.folcher@ird.fr; 5 Université de Lorraine, 54000 Nancy, France; arnaud.bianchi@univ-lorraine.fr; 6 SAINBIOSE U1059, INSERM, Mines Saint Etienne, Université Jean Monnet Saint-Etienne, 42023 Saint-Etienne, France; mireille.thomas@univ-st-etienne.fr (M.T.); solene.viallon@univ-st-etienne.fr (S.V.); sylvie.peyroche@univ-st-etienne.fr (S.P.); sarah.nahle@univ-st-etienne.fr (S.N.); 7 UMR5510 MATEIS, CNRS, INSA-Lyon, University of Lyon, 69621 Lyon, France

**Keywords:** sponge, clathrinida, *Pericharax heteroraphis*, clathridine, zinc complex, osteoporosis

## Abstract

As a result of screening a panel of marine organisms to identify lead molecules for the stimulation of endochondral bone formation, the calcareous sponge *Pericharax heteroraphis* was identified to exhibit significant activity during endochondral differentiation. On further molecular networking analysis, dereplication and chemical fractionation yielded the known clathridine A-related metabolites **3–6** and the homodimeric complex (clathridine A)_2_ Zn^2+^ (**9**), together with the new unstable heterodimeric complex (clathridine A–clathridimine)Zn^2+^ (**10**). With the presence of the zinc complexes annotated through the LC-MS analysis of the crude extract changing due to the instability of some metabolites and complexes constituting the mixture, we combined the isolation of the predicted molecules with their synthesis in order to confirm their structure and to understand their reactivity. Interestingly, we also found a large quantity of the contaminant benzotriazoles BTZ (**7**) and its semi-dimer (BTZ)_2_CH_2_ (**8**), which are known to form complexes with transition metals and are used for preventing corrosion in water. All isolated 2-aminoimidazole derivatives and complexes were synthesized not only for structural confirmation and chemical understanding but to further study their bioactivity during endochondral differentiation, particularly the positively screened imidazolone derivatives. Compounds leucettamine B, clathridine A and clathridimine were found to increase type X collagen transcription and stimulate endochondral ossification in the ATDC5 micromass model.

## 1. Introduction

Osteoporosis represents a major threat for people, especially women suffering from high risk for fractures after age 50. One in three women and one in five men over this age are affected by a broken bone due to osteoporosis [1,2]. The increased risk of bone fractures is closely associated with a reduction in healing that is affected by osteoporosis disorders [3]. More broadly, failure of bone fracture healing occurs in 5% to 10% of all patients [2].

**Figure 1:**
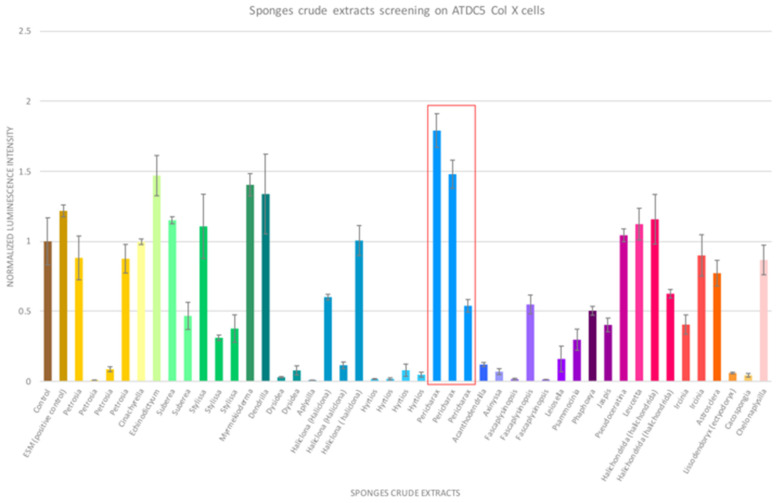
Screening results of 47 marine sponge species revealing the osteogenic activity of the calcareous sponges framed in red.

Considering the efforts devoted to the improvement of bone repair, we decided to explore the potential of marine organisms, namely sponges. Marine sponges are interesting producers of structurally diverse specialized metabolites that exhibit a broad spectrum of potent biological activities [4]. Interestingly, through a random screening of a panel of 47 sponges collected in the Wallis Island lagoon, two of the three calcareous sponges present in the panel revealed significant activity during endochondral differentiation and the biomineralization process (Figure 1). Therefore, we were intrigued by the activity during endochondral differentiation of these calcareous sponges, which are naturally endowed with the faculty to control their own biomineralization [5].

It is indeed interesting to note that the invertebrate calcareous sponges (class Calcarea) can produce spicules using calcium carbonate [6], while most of vertebrate skeletons adapt calcium phosphates as the building material. The question regarding the evolutionary process switching from the calcium carbonate to calcium phosphate in the skeletal tissues of vertebrates is not clear [7]. 

As far as we are concerned, apart from the fact that the molecular diversity generating the bioactivities of marine sponges are impressive, there is no established argument supporting the prediction of which phylum may yield optimal drug precursors for a given target! While the search for bioactive metabolites is often linked to opportunities devoid of any rationality, it is not uncommon that certain investigations lead to meaningful research findings. In our present case, the common property of the mineralization of bioactive calcareous sponges and bones to perform the transformation of calcium into an adapted and functional skeletal structure raises the question of whether common molecular factors would stimulate a common process [8]. Similar research has been carried out on bioactive nacre during bone biomineralization [9]. Such studies dedicated to calcareous sponge metabolites active during endochondral differentiation are lacking so far. For this purpose, we promptly decided to chemically investigate these calcareous sponges, which were the only ones to respond positively to the performed activity during endochondral differentiation screening. From a chemical point of view, some other calcareous sponges were studied. Several species of the Clathrinida order have been investigated and have often been found to contain 2-aminoimidazole/2-aminoimidazolone alkaloids and their derivatives. Broadly, one can distinguish clathridines, naamidines, naamines and leucettamines by their structural particularities [10,11]. Basically, they contain a central 2-aminoimidazole or 2-aminoimidazolone ring, N- or C-substituted by one or more methyl group or functionalized benzyl groups. Further substitutions of the amino function with a hydantoin motif or complexation by zinc increases the molecular diversity and, consequently, the interest in this family of molecules.

We recently reported the isolation and synthesis of the new glycerol ethers pericharaxins A (**1**) and B (**2**) from *Pericharax heteroraphis* [12]. In this study, additional investigations were conducted on the chemical composition of *Pericharax heteroraphis* metabolites, which led to the isolation of 10 compounds. Apart from leucettamine B (**3**), our study revealed that the composition of our South Pacific Ocean specimen is relatively different from those two other studies have already described for the same sponge species collected from the South China [13] and the Indo-Pacific Seas [14]. 

Further biological evaluations revealed that several 2-aminoimidazolones metabolites stimulate endochondral ossification. Endochondral ossification is an essential process of building bone and for the reparative phase of fracture healing [15]. For this purpose, we have used the mice cell line ATDC5 as a model able to reproduce the first stages of endochondral ossification [16]. This model has the advantage of presenting spatial and temporal correlations between the synthesis of type X collagen and the occurrence of endochondral ossification [17]. We developed a rapid in vitro screening platform to screen pro-endochondral differentiation small molecules, including sponges’ extracted molecules and the newly synthesized compounds. We screened compounds for their endochondral differentiation capacity using the human Col X–promoter luciferase reporter (hCol X–luc) transduced into the mouse chondrogenic line ATDC5. The most active compounds (leucettamine B and clathridine derivatives and complexes) are able to stimulate Col X transcription. These selected compounds were then tested on the ATDC5 micromass model [18] to investigate their capacity to stimulate endochondral differentiation and mineralization of the extracellular matrix.

## 2. Results and Discussion

### 2.1. Isolation and Struture Elucidation

**Figure 2:**
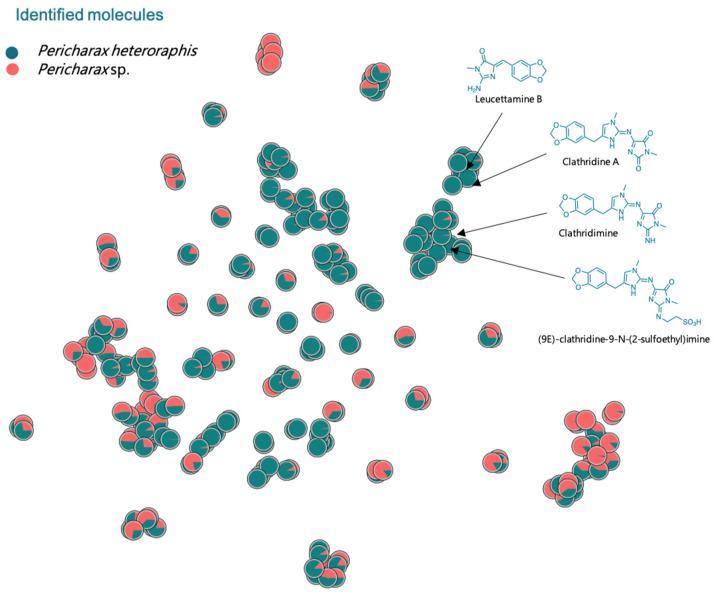
t-SNE graphical representation of the selected MS/MS features from the samples constructed with MetGem. Minimal cosine score was set at 0.75. Nodes colors correspond to the relative proportions of each feature in the sample groups: *Pericharax heteroraphis* and *Pericharax* sp.

The sponges *Pericharax heteroraphis* and *Pericharax* sp. were collected in the Wallis Island lagoon during the Wallis 2018 expedition [19]. The freeze-dried sponges were extracted with a mixture of CH_2_Cl_2_/MeOH: 1/1 and further partitioned with *n*-BuOH/H_2_O. The butanolic extracts were examined by LC-MS/MS for the generation of the molecular network and structures prediction using MetGem 1.3.6 software [20,21]. This allowed the annotation of several clusters, including those of the known 2-aminoimidazolone markers (Figure 2). Due to the lack of large and accessible databases on the marine world, the dereplication process remains complicated. For example, Loratadine, a synthetic antihistaminic, was suggested by MetGem but is likely not present in our extract. Oroidin and dibromophakelin were also annotated, but they are characteristic of other classes of sponges and were never found in a calcarea sponge. The green cluster on the right of the network contains the major compounds of the sponge *Pericharax heteroraphis.* As these compounds were detected with low retention times, we decided to focus on the polar part of this sponge. At the end of the study, all the identified 2-aminoimidazole derivatives were shown to belong to this specific cluster. The most remarkable of this annotation is the presence of the feature clathridine derivatives and leucettamine B.

From this preliminary analysis, we decided to chemically explore the extract of the sponge *Pericharax heteroraphis.*

**Figure 3:**
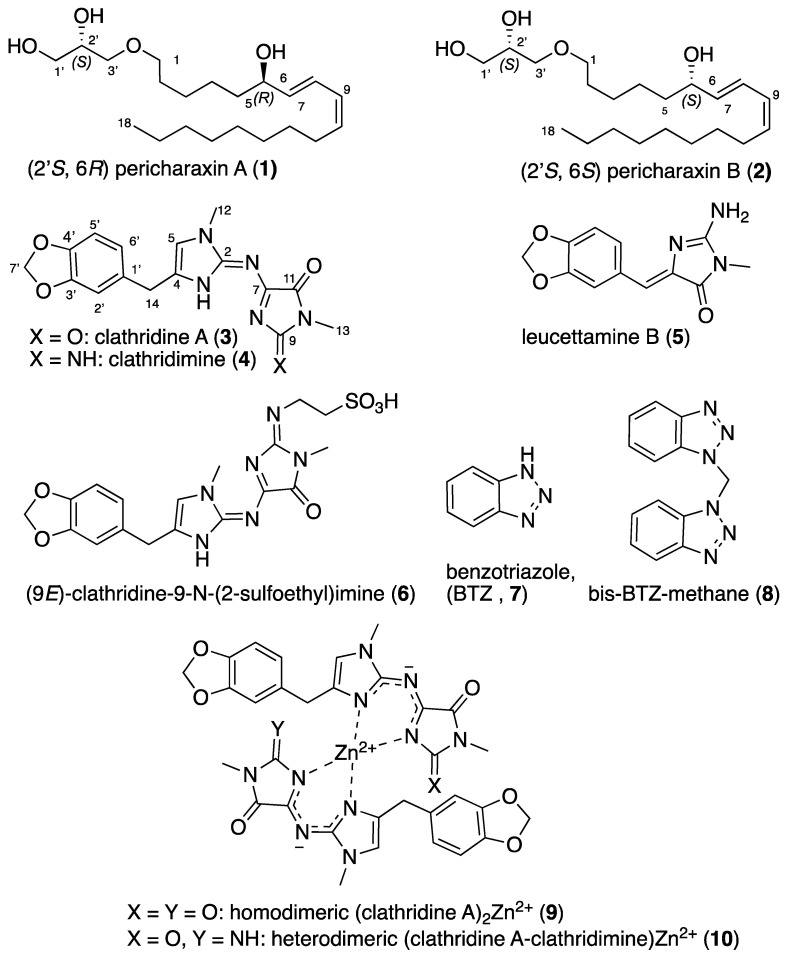
Structures of the 2-aminoimidazolone derivatives **1**–**6**, the contaminants BTZ (**7**) and bis-BTZ (**8**) and Zn complexes **9** and **10**.

The butanolic extract (1.4 g) was submitted to C_18_ reverse-phase MPLC chromatography and eluted with successive solvents H_2_O, H_2_O/CH_3_CN gradient, CH_3_CN, CH_2_Cl_2_ and THF to afford four fractions. Stepwise repetitive purifications using various reverse-phase HPLC purification methods finally resulted in the isolation of 10 compounds (Figure 3). In addition to the glycerol ethers pericharaxins A (**1**) and B (**2**) compounds, we isolated clathridine A (**3**) [22], clathridimine (**4**) [23], leucettamine B (**5**) [24], (9*E*)-clathridine 9-N-(2-sulfoethyl)imine (**6**) [25] and the probably contaminating benzotriazoles **7** and **8** (Figure 1). We were particularly interested in the homodimeric zinc complex **9** [22] and the new heterodimeric (clathridine A–clathridimine) zinc complex (**10**). It should be noted that the isolation of clathridimine is not easy due to its instability and, consequently, its low quantity [23]. 

We will mainly focus on the zinc complexes, but first, there were some questions about the presence of benzotriazole (**7**) and its dimer (**8**). The benzotriazoles **7** and **8** are undoubtedly contaminants. Nevertheless, their presence in a sponge in such large quantities was intriguing. We verified its origin and confirmed that they were present in the extract of the sponge and not in the extraction solvents or vessels. Indeed, other sponges were treated under the same conditions, and the corresponding extracts did not contain benzotriazole. Its presence in *Pericharax heteroraphis* would depend either on the place of collection or on the species of sponge that would have the ability to concentrate such contaminants. Benzotriazole (**7**) is known as a synthetic product commonly used as an anticorrosive additive [26] or as a UV stabilizer [24]. The sponge was indeed harvested near the Port of Halalo in Wallis, which could be highly contaminated. We looked at another sponge of the genus Echinodyctium, which was collected from exactly the same place as *Pericharax heteroraphis*, and no trace of benzotriazole (**7**) was detected. The explanation for this accumulation is not obvious. It should be noted that benzotriazole (**7**) is known to complex metals like Zn(II), Cu(II), Ni(II) and Co(II) [27].

We next isolated the natural complexes and studied their reactivity. If the homodimeric complex **9** could be isolated and characterized, the minor heterodimeric clathridine A–clathridimine zinc complex **10** was not stable enough to be isolated. More generally, the LC-MS successive profiles of the crude extract and the fractions were not reproductible, which gave a certain inconstancy to the results of the purifications of the minor complexes. The co-isolation of clathridine A (**3**) and clathridimine (**4**) from the same sponge and the LC-MS indications pointing out several Zn dimers clearly raises the question of the formation of various homodimeric and heterodimeric crossed complexes of these two monomers. It should be noted that the homodimeric complex of clathridimine and the heterodimeric zinc complex of clathridine A–clathridimine have, to our knowledge, not been reported. Thus, we decided to synthetize the monomers and their Zn complexes in order to understand and examine their presence in the crude extract, their stability and, finally, to prepare sufficient quantities of material for their osteogenic activity evaluations. 

### 2.2. Synthesis of Clathridine A (***3***), Clathridimine (***4***) and Leucettamine B (***5***)

**Scheme 1:**
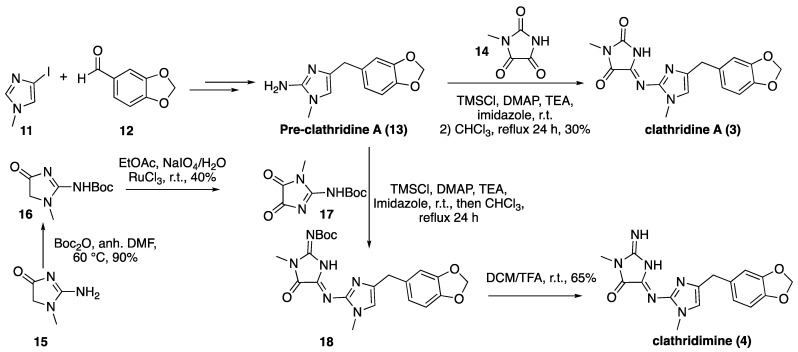
Synthesis steps from preclathridine A (**13**) to clathridine A (**3**) and clathridimine (**4**).

The synthesis of clathridine A (**3**) was achieved following the protocols described by the group of Lovely [28] and references cited therein. Metallation of the methyliodoimidazole **11** (Scheme 1), followed by treatment with piperanal (**12**), reduction and amination of imidazole position 2 through azidation delivered the preclathridine A (**13**). The piperanal fragment (**12**) was obtained from piperonyl alcohol using the method described by Cossio et al. [29]. Boc clathridine A (**3**) was obtained by the condensation of the preclathridine A (**13**) with the activated parabanic acid derivative **14** in a 30% yield (Scheme 1). The next objective was the new preparation of the clathridimine (**4**) that was never achieved before. We decided to follow a similar pathway as for the synthesis of clathridine A (**3**) by Lovely and adapted the method for the introduction of the imine function. The Boc-protected imidazodione **17** was prepared from creatinine (**15**) by adapting the method of Yamamoto et al. [30,31]. The highly toxic mercury(II) acetate was avoided. Thus, creatinine (**15**) was protected by di-*tert*-butyl-dicarbonate to quantitatively achieve the intermediate **16** (Scheme 1), which was then oxidized in the presence of ruthenium(III) chloride and an aqueous solution of sodium periodate to give the N-Boc-creatone A (**17**). The preclathridine A (**13**) was coupled with the protected compound **17** on the terminal amine using the same reaction as for the synthesis of clathridine A (**3**). The coupling crude product **18** was deprotected in the presence of TFA to give clathridimine (**4**) in a 65% yield. We have to mention that the protection of creatone A in the form of N-Boc **17** was made necessary because of its instability under the reaction conditions when used without protection. The Dimroth rearrangement [31] is the probable reason for the reaction failure, causing the simultaneous presence of two regioisomers of creatone A.

The structures of the synthetic preclathridine A (**13**), clathridine A (**3**) and clathridimine (**4**) were confirmed by HR-ESIMS and a comparison of their ^1^H-NMR and ^13^C-NMR spectral data with those of the natural preclathridine A [32], clathridine A isolated in this work and clathridimine [23].

**Scheme 2:**
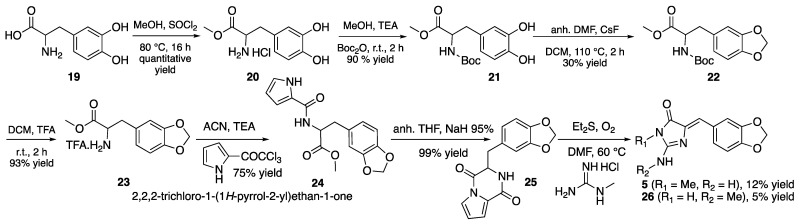
Synthesis of leucettamine B (**5**).

Leucettamine B (**5**) was prepared using our technology based on the key oxidative step involving a pyrrole moiety [33,34,35] applied to the diketopiperazine **25** (Scheme 2) in the presence of methylguanidine.

The diketopiperazine **25** was prepared by the base-induced cyclization of the pseudopeptide **24**, which was prepared by the acylation of **23** by trichloroacylpyrrole (Scheme 2). Compound **23** could be synthesized in four steps from L-DOPA **19** after quantitative esterification into **20**, N-Boc protection in a 90% yield, catechol dioxolane **22** preparation and amine deprotection with TFA. As described [36], the installation of the methylenedioxy-protecting group was not trivial and led to only a 30% yield. Finally, the addition of methylguanidine to **25** led to leucettamine B (**5**) at 60 °C in a 12% yield. Even if the observed conversion of this reaction is 50%, the purification and separation of regioisomers leucettamine B (**5**) and **26** do not allow a good isolated yield. We have not sought to improve the yield in this work, but we intend to do so in the near future due to the modularity of this reaction to prepare various analogs of leucettamine B.

### 2.3. Complexation of Clathridine A (***3***) and Clathridimine (***4***)

**Scheme 3:**
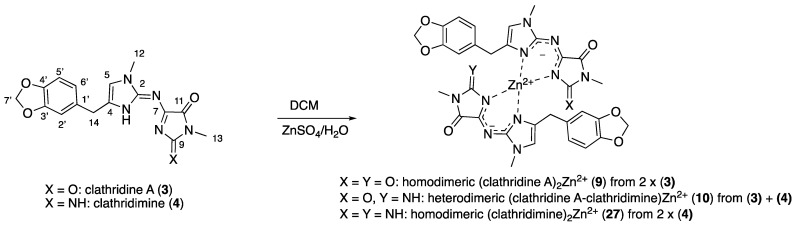
Preparation and structures of the three homo- and heterodimeric complexes **9**, **10** and **27**.

As explained in the isolation part described above, clathridine A (**3**) and clathridimine (**4**) thus synthesized were used to carry out zinc complexation tests and to observe the stability of the complexes formed. The homodimeric clathridine A zinc complex **9** was synthetized following the procedure of Ciminiello et al. [22]. The synthesis of homodimeric clathridimine zinc complex **27** (Scheme 3) was performed by the same method, mixing a dichloromethanolic solution of clathridimine with an aqueous solution of zinc sulfate. 

The X-Ray diffraction structure for our crystals (see Supplementary Materials Figure S48) is similar to the structure published in the literature [37]. 

Crystals of Zn complex **9** were obtained by slow evaporation at room temperature. X-Ray diffraction and crystallographic data were collected at room temperature using redundant ω scans on a Rigaku XtaLabPro single-crystal diffractometer using microfocus Mo Kα radiation. X-Ray analysis experimental details, the ORTEP view, crystal data and structure refinement are available in the Supplementary Materials. The crystallographic data have been deposited in the Cambridge Crystallographic Data Center as entry 2299450 and can be obtained, free of charge, upon application to the Director, CCDC, 12 Union Rd., Cambridge CB21EZ, UK [fax, + 44(0)-1233-336033; email, deposit@ccdc.cam.ac.uk].

Finally, the synthesis of the clathridine–clathridimine heterodimeric zinc complex (**10**) was achieved by mixing both clathridine A (**3**) and clathridimine (**4**) in equimolecular concentrations with a zinc sulfate aqueous solution. Three different complexes were obtained: clathridine A and clathridimine zinc complexes **9** and **27,** previously described, and the new heterodimeric clathridine A–clathridimine zinc complex **10**, as well. Mass spectra and NMR data of the complex **9** agree with the literature data and those of our isolated one. NMR data of the three complexes are in Table 1.

Although the complexes were clearly detected in the LC-MS profile (see Supplementary Materials Figure S45), the clathridine A–clathridimine heterodimeric complex (**10**) could not be isolated as a single entity. We observed an equilibrium depending on the operating conditions, particularly the concentration and the humidity. It is important to remember here that clathridimine (**4**) was localized in the sponge cells [23], and its hydrolysis into clathridine A (**3**) seems to be an easy process. The article cited previously that describes it therefore raises the question of its presence and stability in a marine environment.

The HRMS data and retention times allowed us to confirm the presence of this new clathridine A–clathridimine heterodimeric complex (**10**) in the sponge (See Supplementary Materials Figure S45). The clathridimine Zn complex (**27**) revealed itself much less stable than the others. It may be present in the sponge but not detectable because of its low concentration and instability. These observations raise the question of the existence of clathridimine in the natural aqueous medium and, consequently, the effect of complexation on its stability in marine environments.

The superposition of the NMR spectra of the complexes (see Supplementary Materials Figures S46 and S47) clearly shows the existence of the mixtures observed in HPLC.

To estimate the role of complexation in the clathridimine stability, we calculated the energy of the intermediates in the hydrolysis reaction for both complexed and free clathridimine. In the first attempt, the clathridimine structure was simplified and the hydrolysis reaction was considered as the reaction between one molecule of water and one molecule of clathridimine. With these approximations, the hydrolysis of the complexed form was shown to be easier than the free form, contrary to our assumption suggested by the experimental observations (see Supplementary Materials Figure S49).

Further, the same calculations were performed considering two molecules of water involved in the hydrolysis reaction (see Supplementary Materials Figure S50). In this case, the energy barriers were lower, in line with a reduced strain in the 6-membered ring cyclic intermediates. Furthermore, the activation barriers for the complexed and free forms of clathridimine were virtually the same. Following this trend, we may expect that systems containing more water molecules, which better reproduce reality, would be in agreement with the experimental observation, with a lower activation energy for the free clathridimine compared with the Zn-complexed form. Clathridimine hydrolyzes less quickly when it is complexed with zinc.

### 2.4. Biological Screening

**Figure 4:**
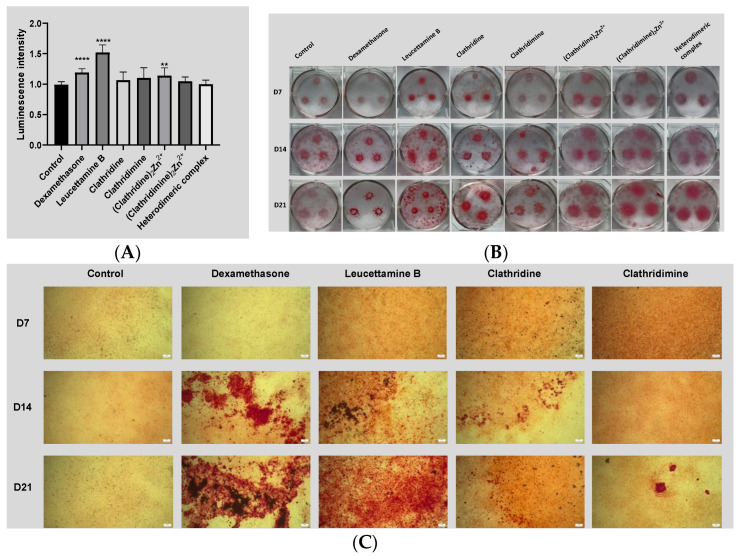
(**A**) Results of the assessment of an ATDC5-based rapid screening platform using human Col X (hCol X) proximal promoter activity for the selection of natural compounds able to stimulate endochondral differentiation. Dexamethasone can be considered in this assay as a positive control. Statistical significance is estimated by Kruskal–Wallis Dunn’s multiple comparison test (** = *p* < 0.005 and **** = *p* < 0.0001). (**B**) Effect of leucettamine B and clathridine A derivatives and complexes on the endochondral differentiation of ATDC5 cells. ATDC5 cells were treated in 6-well plates for 7, 14 and 21 days for Alizarin red staining (presence of calcification). (**C**) Enlargement by microscope of the effect of leucettamine B and clathridine A derivatives on the endochondral differentiation of ATDC5 cells. ATDC5 cells were treated for 7, 14 and 21 days for Alizarin red staining (presence of calcification) (scale bar = 200 µm). The corresponding pictures for the complexes are available in the Supplementary Materials.

Leucettamine B and the clathridine derivatives and complexes were evaluated on a screening platform for endochondral differentiation activity using human type X collagen transcription activity in ATDC5 cells (Figure 4). Leucettamine B is the compound that demonstrated significatively the better induction of the Col X promoter, even more so than Dexamethasone, considered here as a positive control. Clathridine A and clathridimine presented an activation, but not a significant one, when the (clathridine A)_2_Zn^2+^ complex demonstrated some significant activity but lower than Dexamethasone. All these compounds were then tested on the ATDC5 micromass model (Figure 4B,C). 

**Figure 5:**
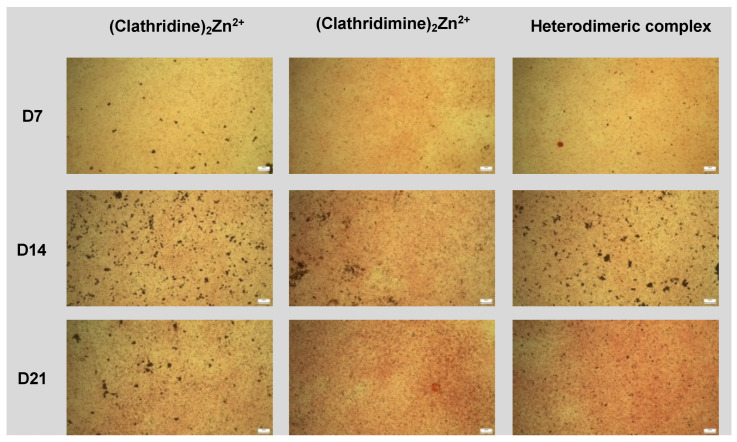
Enlargement by microscope of the effect of clathridine A/clathridimine complexes on the endochondral differentiation of ATDC5 cells. ATDC5 cells were treated for 7, 14 and 21 days for Alizarin red staining (presence of calcification) (scale bar = 200 µm).

In this model, after 7, 14 and 21 days of culture, Alizarin red staining was used to proof the matrix mineralization. The control consisted of untreated cells. Only Leucettamine B demonstrated a mineralization induction capacity as high as Dexamethasone when clathridine and clatridimine present a lower one (Figure 5). By Alizarin red quantification, we can analyze that the homo- and heterodimer complexes showed limited capacity in inducing mineralization in comparison to Leucettamine B (Supplementary Figure S50).

The most active compounds on the screening tool (ATDC5 Col X) have been confirmed to promote endochondral ossification in a micromass model. Between these compounds, Leucettamine B (**5**) is the one that induces endochondral differentiation as high as Dexamethasone. It seems that clathridimine has less of an effect than clathridine A on endochondral ossification. 

Leucettamine B (**5**) has recently demonstrated some inhibitory activity on DYRK and CLK, but it is the first time that its activity on endochondral ossification has been reported. Leucettamine B belongs to the few candidates that may be used in the future to promote bone repair where endochondral differentiation and ossification are involved.

## 3. Conclusions

The isolated 2-aminoimidazole derivatives and complexes were synthesized not only for structural confirmation and chemical understanding but to further study their bioactivity on endochondral differentiation, particularly the positively screened imidazolone derivatives. Although molecular networks offer a rapid and visual approach, their use in marine environments still require the constriction of databases. For the moment, the isolation of compounds and their characterizations remain the only privileged way to exploit and valorize them.

Compounds leucettamine B, clathridine A and clathridimine were found to increase type X collagen transcription and stimulate endochondral ossification in the ATDC5 micromass model. Clathridimine, the homodimeric zinc complex of clathridimine and the heterodimeric zinc complex of clathridine A–clathridimine were synthetized for the first time.

The co-isolated leucettamine B was synthesized through our technology using diketopiperazines oxidation and the addition of guanidines. 

Leucettamine B, the most active compound, is able to induce endochondral ossification in vitro in a better way than Clathridine A, Clathridimine individually or their homo- and heterodimeric complexes. These compounds, capable of stimulating endochondral ossification, represent potential candidates for improving bone repair. Their production would potentially be formulated from synthetic products, because sponges have, until now, been recalcitrant to cultures. Although sponge farming seems the most sustainable to develop, organic synthesis sometimes allows for more sustainable and economically competitive production processes.

## 4. Materials and Methods 

### 4.1. General Procedures

IR spectra were recorded on a Perkin Elmer BX FT-IR spectrometer (Perkin Elmer, Waltham, MA, USA). NMR spectra were recorded on a Bruker Avance 300 spectrometer or a Bruker Avance 500 spectrometer (Bruker, Billerica, MA, USA). The chemical shifts were reported in ppm relative to the residual signal solvent (MeOH-*d*_4_: *δ*_H_ 3.31, *δ*_C_ 49.15; CDCl_3_: *δ*_H_ 7.26, *δ*_C_ 77.16). High-resolution mass spectra were obtained on a LCT Premier XE spectrometer (Waters Corporation, Milford, NY, USA) in electrospray ionization mode by direct infusion of the purified compounds. Medium-Pressure Liquid Chromatography (MPLC) was performed on a Puriflash apparatus (Interchim, Montluçon, France) using a Silica gel Interchim cartridge PF-50SIHP (Interchim, Montluçon, France). The synthesis reactions were monitored by thin-layer chromatography (TLC). The TLC was performed on aluminum precoated silica gel 60 F254 plates (Merck, Darmstadt, Germany) and revealed with a solution of sulfuric vanillin. Preparative HPLC purifications were performed on an autoprep system: Waters 600 controller and Waters 600 pump with a Waters 996 photodiode array detector (Waters Corporation, Milford, NY, USA), equipped with a Waters Sunfire C_18_ (19 × 150 mm, 5 μm) column. Organic solvents were purchased from Carlo Erba (Val de Reuil, France). All other chemicals were purchased from Sigma-Aldrich (St. Louis, MO, USA) or Fluorochem (Hadfield, UK). 

### 4.2. Animal Material

Sponges were collected by hand using SCUBA in Wallis and Futuna during the sampling cruise Wallis 2018 aboard the R/V Alis off the coast of Wallis Island (18 July 2018–31, 13°16.367′ S; 176°12.283′ W) between 6 and 50 m deep [19]. Voucher samples are deposited at the Queensland Museum (Brisbane, Australia) and were identified by Dr. Merrick Ekins, accession numbers available in the Supplementary Materials (Table S1). Sponges were deep-frozen on board until workup. They were then freeze-dried and grounded before extraction.

The sponge *Pericharax heteroraphis* Poléjaeff 1883 was collected in the lagoon of Wallis Island (13°16.177’ S, 176°08.120’ W) at 15 m deep in July 2018. The corresponding voucher specimen is available under accessing number G339027.

### 4.3. HPLC-QTOF-ESI-MS/MS Analysis

LC-MS/MS analyses were performed using the methods described in the work of Schilling et al. [38].

### 4.4. Network Metabolomic Analyses

The molecular network of the *Pericharax* samples was constructed according to the procedure described by Schilling et al. [38].

### 4.5. Extraction and Isolation of Compounds ***3***, ***5***, ***6*** and ***9***

The 47 sponge extracts preliminary tested were obtained by a pressurized liquid extraction process with a mixture of CH_2_Cl_2_/MeOH (1:1), as previously described [39]. 

The freeze-dried sponge sample of *Pericharax heteroraphis* (93 g) was extracted by ASE at 40 °C under pressure (100 bar) three times with a mixture of CH_2_Cl_2_/MeOH (1:1). The extracts were combined and dried under reduced pressure to afford a brown residue (6 g), and 5.5 g of this residue was partitioned between *n*-BuOH and H_2_O. The butanolic extract (1.4 g) was fractioned by C_18_ reverse-phase MPLC eluted with successive solvents (H_2_O, H_2_O/CH_3_CN gradient, CH_3_CN, CH_2_Cl_2_ and THF) to afford fractions F1 to F4.

Fraction F2 (H_2_O/CH_3_CN gradient) was purified by preparative reverse-phase HPLC (column: Waters Sunfire C_18_, 19 mm × 150 mm, 5 μm, H_2_O + 0.1% formic acid/CH_3_CN + 0.1% formic acid) to obtained four 2-aminoimidazoles: clathridine A (**3**) (1.9 mg), leucettamine B (**5**) (1.2 mg), (9*E*)-clathridine-9-N-(2-sulfoethyl)imine (**6**) (1.2 mg) and homodimeric (clathridine A)_2_ Zn^2+^ (**9**) (2.4 mg). 

*Clathridine A* (**3**): yellow solid (1.9 mg); UV (MeOH) λ_max_ (log *ε*) 203 (0.18), 215 (0.18), 283 (0.01), 388 (0.03) nm; ^1^H NMR (500 MHz, CDCl_3_): *δ* (ppm) 6.75 (1H, d, *J* = 7.8 Hz, H-5′), 6.73 (1H, s, H-2′), 6.71 (1H, d, *J* = 7.8 Hz, H-6′), 6.53 (1H, s, H-5), 5.94 (2H, s, H-7′), 3.81 (2H, s, H-14), 3.72 (3H, s, H-12), 3.19 (3H, s, H-13); ^13^C NMR (125 MHz, CDCl_3_): *δ* (ppm) 161.8 (C-9), 154.6 (C-11), 147.6 (C-5′), 146.5 (C-2), 146.3 (C-3′), 139.5 (C-4), 132.6 (C-1′), 121.6 (C-6′), 117.5 (C-5), 109.2 (C-2′), 108.2 (C-5′), 100.8 (C-7′), 34.4 (C-14), 32.0 (C-12), 24.6 (C-13) (C-7 non detected); HRESIMS *m*/*z* 342.1172 [M + H]^+^ (calcd for C_16_H_16_N_5_O_4_, 342.1202).

*Leucettamine B* (**5**): clear oil (1.2 mg); UV (MeOH) λ_max_ (log *ε*) 234 (0.38), 367 (0.72) nm; ^1^H NMR (500 MHz, CDCl_3_): *δ* (ppm) 7.41 (1H, s, H-1 ar), 7.21 (1H, d, *J* = 8.0 Hz, H-2 ar), 6.65 (1H, d, *J* = 8.0 Hz, H-3 ar), 6.83 (1H, s, CH), 6.02 (2H, s, CH2 benzodioxol), 3.30 (3H, s, CH3); ^13^C NMR (125 MHz, CDCl_3_): *δ* (ppm) 165.2 (CO), 157.4 (CNH_2_), 149.6 (C-ar), 149.2 (C-ar), 126.6 (C-2), 119.1 (CH) 109.8 (C-1), 109.0 (C-3), 101.8 (CH_2_), 26.0 (CH3); HRESIMS *m*/*z* 246.0846 [M + H]^+^ (calcd for C_12_H_12_N_3_O_3_, 246.0879).

*(9E)-Clathridine-9-N-(2-sulfoethyl)imine B* (**6**): clear oil (1.2 mg); UV (MeOH) λ_max_ (log *ε*) 217 (0.38), 261 (0.25), 385 (0.38) nm; ^1^H NMR (500 MHz, CDCl_3_): *δ* (ppm) 6.84 (1H, s, H-2′), 6.77 (2H, s, H-5′ and H-6′), 6.35 (1H, s, H-5), 5.95 (2H, s, H-7′), 4.25 (2H, m, H-16), 4.09 (2H, s, H-14), 3.71 (3H, S, H-12), 3.35 (3H, S, H-13), 3.33 (2H, m, H-17); ^13^C NMR (125 MHz, CDCl_3_): *δ* (ppm) 165.2 (CO), 157.4 (CNH_2_), 149.6 (C-ar), 149.2 (C-ar), 126.6 (C-2), 119.1 (CH) 109.8 (C-1), 109.0 (C-3), 101.8 (CH_2_), 26.0 (CH3); HRESIMS *m*/*z* 449.1257 [M + H]^+^ (calcd for C_18_H_21_N_6_O_6_S, 449.1243).

*Homodimeric (clathridine A)_2_ Zn^2+^* (**9**): yellow solid (2.4 mg); UV (MeOH) λ_max_ (log *ε*) 220 (0.33), 286 (0.03), 368 (0.09) nm; ^1^H NMR (500 MHz, CDCl_3_): *δ* (ppm) 6.63 (2H, s, H-5), 6.50 (2H, d, *J* = 7.9 Hz, H-5′), 6.26 (2H, t, *J* = 7.9 Hz, H-6′), 6.25 (2H, s, H-2′), 5.88 (4H, dd, *J* = 13.6, 1.1 Hz, H-7′), 3.80 (6H, s, H-12), 3.51 (2H, d, *J* = 16.3 Hz, H-14a), 3.37 (2H, d, *J* = 16.3 Hz, H-14b), 3.04 (2H, s, H-13); ^13^C NMR (125 MHz, CDCl_3_): *δ* (ppm) 164.9 (C-9), 161.4 (C-11), 149.0 (C-2), 147.7 (C-3′), 146.5 (C-4′), 136.3 (C-4), 131.1 (C-1′), 121.7 (C-2′), 117.7 (C-5), 108.6 (C-6′), 107.9 (C-5′), 101.2 (C-7′), 33.3 (C-14), 32.7 (C-12), 24.7 (C-13); HRESIMS *m*/*z* 745.1479 [M + H]^+^ (calcd for C_32_H_29_N_10_O_8_Zn, 745.1461).

### 4.6. Total Synthesis of Clathridine A (***3***) and Clathridimine (***4***)

*Piperonal* (**12**): To a suspension of pyridinium dichromate (18.55 g, 49 mmol, 1.5 eq.) in dry DCM (230 mL), trichloromethylsilane (14.6 mL, 115 mmol, 3.5 eq.) was added dropwise at 0 °C. After 15 min, piperonyl alcohol (5 g, 33 mmol, 1 eq.) was added, and the reaction mixture was stirred for 1 h at room temperature. Moist silica gel was then added, and the suspension was filtered through a pad of silica gel. The solvent was evaporated under reduced pressure, and the crude product was purified by flash column chromatography on silica gel (gradient 100% Heptane to70:30 Heptane/EtOAc) to give **12** as an oil (3.76 g, 75% yield). ^1^H NMR (300 MHz, CDCl3): *δ* (ppm) 9.78 (1H, s, H-14), 7.38 (1H, dd, *J* = 7.8, 1.6 Hz, H-6′), 7.26 (1H, d, *J* = 1.6 Hz, H-2′), 6.86 (1H, d, *J* = 7.8 Hz, H-5′), 6.00 (2H, s, H-7′); ^13^C NMR (75 MHz, CDCl3): *δ* (ppm) 190.2 (C-14), 153.1 (C-4′), 148.7 (C-3′), 132.0 (C-1′), 128.6 (C-6′), 108.3 (C-5′), 106.8 (C-2′), 102.1 (C-7′); ESIMS *m*/*z* 151.03 [M+H]^+^.

*Preclathridine A* (**13**): Prepared in four steps according to the procedure described by Koswatta et al. [28,40]. 4-iodo-1-methyl-1*H*-imidazole **11** (5.5 g, 27 mmol, 1 eq.) in anhydrous THF (96 mL), EtMgBr (3.0 M solution in ether, 9.4 mL, 28 mmol, 1.05 eq.) and piperonal **12** (4.2 g, 28 mmol, 1.05 eq.) gave intermediate *4-(Benzo[1,3]dioxol-5-yl)hydroxymethyl-1-methyl-1H-imidazole* (4.31 g, 70% yield) as an orange solid. (^1^H NMR (300 MHz, CDCl3): *δ* (ppm) 7.41 (1H, br s, H-2), 6.94 (1H, br s, H-2′), 6.91 (1H, dd, *J* = 7.9, 1.6 Hz, H-6′), 6.76 (1H, d, *J* = 7.9 Hz, H-5′), 5.93 (2H, s, H-7′), 5.70 (1H, s, H-14), 3.60 (3H, s, H-12); ^13^C NMR (75 MHz, CDCl3): *δ* (ppm) 147.6 (C-4′), 146.0 (C-4), 145.9 (C-3′), 137.5 (C-2), 137.1 (C-1′), 120.1 (C-6′), 116.9 (C-5), 107.9 (C-5′), 107.3 (C-2′), 100.9 (C-7′), 70.4 (C-14), 33.4 (C-12); HRESIMS *m*/*z* 233.0926 [M+H]^+^ (calcd for C_12_H_13_N_2_O_3_, 233.0926)). Then, this coupling product (4.3 g, 18.5 mmol, 1 eq.) in anhydrous DCM (140 mL), TFA (5.7 mL, 74 mmol, 4 eq.) and Et_3_SiH (13.6 mL, 85 mmol, 4.5 eq.) gave *4-(Benzo[1,3]dioxol-5-yl)methyl-1-methyl-1H-imidazole* (2.3 g, 60% yield) as a yellow solid. (^1^H NMR (300 MHz, CDCl3): *δ* (ppm) 7.34 (1H, br s, H-2), 6.76 (1H, br s, H-2′), 6.72 (2H, s, H-5′, H-6′), 6.51 (1H, s, H-5), 5.89 (2H, s, H-7′), 3.81 (2H, s, H-14), 3.58 (3H, s, H-12); ^13^C NMR (75 MHz, CDCl3): *δ* (ppm) 147.5 (C-4′), 145.7 (C-3′), 142.6 (C-4), 137.3 (C-2), 134.2 (C-1′), 121.5 (C-6′), 116.9 (C-5), 109.3 (C-5′), 108.0 (C-2′), 100.7 (C-7′), 34.6 (C-14), 33.1 (C-12); HRESIMS *m*/*z* 217.0931 [M+H]^+^ (calcd for C_12_H_13_N_2_O_2_, 217.0977)). Then, the previous product (2.2 g, 10.2 mmol, 1 eq.) in anhydrous THF (40 mL), *n*-BuLi (1.6 M in hexane, 7.0 mL, 11.2 mmol, 1.1 eq.) and tosyl azide (30% *w/w* in toluene, 9.0 mL, 12.2 mmol, 1.2 eq.) gave the azide intermediate (1.9 g, 71% yield) as a brownish oil. (^1^H NMR (300 MHz, CDCl3): *δ* (ppm) 6.76 (1H, br s, H-2′), 6.72 (2H, s, H-5′, H-6′), 6.25 (1H, s, H-5), 5.90 (2H, s, H-7′), 3.75 (2H, s, H-14), 3.32 (3H, s, H-12); ^13^C NMR (75 MHz, CDCl3): *δ* (ppm) 147.6 (C-4′), 145.9 (C-3′), 140.1 (C-2), 139.9 (C-4), 133.5 (C-1′), 121.7 (C-6′), 116.0 (C-5), 109.4 (C-5′), 108.1 (C-2′), 100.8 (C-7′), 34.7 (C-14), 31.4 (C-12); HRESIMS *m*/*z* 258.0991 [M+H]^+^ (calcd for C_12_H_12_N_5_O_2_, 258.0991)). This azide intermediate (1.85 g, 7.2 mmol, 1 eq.) was dissolved in EtOH (58 mL) in the presence of 10% Pd/C on charcoal (0.38 g, 0.36 mmol, 5 mol%) to give **13** (1.57 g, 95% yield) as a yellow solid. ^1^H NMR (300 MHz, CDCl3): *δ* (ppm) 6.72 (1H, br s, H-1), 6.70 (2H, s, H-5′, H-6′), 6.05 (1H, s, H-5), 5.89 (2H, s, H-7′), 3.63 (2H, s, H-14), 3.34 (3H, s, H-12); ^13^C NMR (75 MHz, CDCl3): *δ* (ppm) 147.8 (C-2), 147.7 (C-4′), 146.1 (C-3′), 134.5 (C-4), 133.3 (C-1′), 121.8 (C-6′), 112.9 (C-5), 109.5 (C-5′), 108.3 (C-2′), 101.0 (C-7′), 33.7 (C-14), 31.8 (C-12); HRESIMS *m*/*z* 232.1100 [M+H]^+^ (calcd for C_12_H_14_N_3_O_2_, 232.1086).

*Clathridine A* (**3**): Prepared using the method described by Ohta [41]. To a solution of 1-methylparabanic acid (**14**) (0.3 g, 2.4 mmol, 1 eq.) and DMAP (cat.) in anhydrous CHCl_3_ (2.4 mL), triethylamine (0.7 mL, 5 mmol, 2.1 eq.) and then TMS-Cl (0.63 mL, 5 mmol, 2.1 eq.) were added, and the resulting mixture was stirred at room temperature for 2 h under Ar. Then, a solution of preclathridine A (**13**) (0.55 g, 2.4 mmol, 1 eq.) in anhydrous CHCl_3_ (2 mL) was added, and the reaction mixture was stirred at reflux for 24 h in a sealed tube. After returning to room temperature, the reaction was quenched with water (2 mL), and the layers separated. The aqueous layer was extracted with CHCl_3_ (3 × 10 mL), and the combined organic phases were dried over MgSO_4_, filtered and concentrated. The crude product was purified by flash column chromatography on silica gel (gradient 100% DCM to 70:30 DCM/EtOAc) to give **3** (0.250 g, 30% yield) as a yellow solid. ^1^H NMR (300 MHz, CDCl3): *δ* (ppm) 6.74 (1H, br s, H-2′), 6.72 (2H, s, H-5′, H-6′), 6.52 (1H, s, H-5), 5.93 (2H, s, H-7′), 3.82 (2H, s, H-14), 3.71 (3H, s, H-12), 3.18 (3H, s, H-13); ^13^C NMR (75 MHz, CDCl3): *δ* (ppm) 161.8 (C-11), 155.1 (C-9), 152.3 (C-7), 147.6 (C-3′), 146.5 (C-2), 146.3 (C-4′), 138.9 (C-4), 132.3 (C-1′), 121.7 (C-6′), 117.5 (C-5), 109.2 (C-5′), 108.3 (C-2′), 100.9 (C-7′), 34.0 (C-14), 32.2 (C-12), 24.8 (C-13); HRESIMS *m*/*z* 342.1167 [M+H]^+^ (calcd for C_16_H_16_N_5_O_4_, 342.1202).

*tert-butyl-(1-methyl-4-oxo-4,5-dihydro-1H-imidazol-2-yl)carbamate* (**16**): To a solution of creatinine (**15**) (2.0 g, 17.7 mmol, 1 eq.) in anhydrous DMF (7 mL) was added Boc_2_O (4.2 g, 19.4 mmol, 1.1 eq.). The resulting mixture was then stirred at 60 °C overnight. After returning to room temperature, the reaction was quenched with water (10 mL), and the layers separated. The aqueous layer was extracted with EtOAc (3 × 15 mL), and the combined organic layers were dried over MgSO_4_, filtered and concentrated to give **16** (3.5 g, 93% yield) as a pale yellow oil. The crude product was used for the next step without any further purification. ^1^H NMR (300 MHz, CDCl3): *δ* (ppm) 10.44 (1H, br s, NH-Boc), 3.90 (2H, s, H-11), 3.10 (3H, s, N-CH_3_), 1.51 (9H, s, CH_3_ *t*-Bu); ^13^C NMR (75 MHz, CDCl3): *δ* (ppm) 169.5 (C-7), 162.4 (C=O Boc), 159.0 (C-9), 80.1 (Cq *t*-Bu), 51.4 (C-11), 30.7 (C-13), 28.1 (CH_3_ *t*-Bu); ESIMS *m*/*z* 228.15 [M+H]^+^ (calcd for C_9_H_14_N_3_O_3_, 228.10).

*tert-butyl-(1-methyl-4,5-dioxo-4,5-dihydro-1H-imidazol-2-yl)carbamate* (**17**): To a solution of **16** (1.15 g, 5.4 mmol, 1 eq.) in EtOAc (30 mL), a solution of sodium periodate NaIO_4_ (3.5 g, 16.3 mmol, 3 eq.) in water (30 mL) was added, followed by the introduction of ruthenium(III) chloride (1.17 g, 0.80 mmol, 0.15 eq.). The reaction mixture was stirred vigorously at room temperature for 2 h. The layers were then separated, and the aqueous layer was extracted with EtOAc (2 × 50 mL). The combined organic layers were washed with brine, dried over MgSO_4_, filtered and concentrated. The crude product was purified by flash column chromatography on silica gel (gradient 99:1 Heptane/EtOAc to 60:40 Heptane/EtOAc) to give **17** (0.480 g, 40% yield) as a pale yellow solid. ^1^H NMR (300 MHz, CDCl3): *δ* (ppm) 10.38 (1H, br s, NH-Boc), 3.26 (3H, s, N-CH_3_), 1.55 (9H, s, CH_3_ *t*-Bu); ^13^C NMR (75 MHz, CDCl3): *δ* (ppm) 160.5 (C=O Boc), 156.0 (C-9), 155.1 (C-7), 154.4 (C-11), 83.3 (Cq *t*-Bu), 26.3 (CH_3_ *t*-Bu), 28.1 (C-13); HRESIMS *m*/*z* 226.0846 [M-H]^-^ (calcd for C_9_H_12_N_3_O_4_, 226.0828).

*Clathridimine* (**4**): (a) To a solution of compound **17** (0.295 g, 1.3 mmol, 1 eq.) and DMAP (cat.) in anhydrous CHCl_3_ (1.3 mL), triethylamine (0.380 mL, 2.7 mmol, 2.1 eq.) and then TMS-Cl (0.346 mL, 2.7 mmol, 2.1 eq.) were added, and the resulting mixture was stirred at room temperature for 2 h under Ar. Then, a solution of preclathridine A (**13**) (0.300 g, 1.3 mmol, 1 eq.) in anhydrous CHCl_3_ (1.3 mL) was added, and the reaction mixture was stirred at reflux for 24 h in a sealed tube. After returning to room temperature, the reaction was quenched with water (2 mL), and the layers separated. The aqueous layer was extracted with CHCl_3_ (3 × 10 mL), and the combined organic phases were dried over MgSO_4_, filtered and concentrated to give 0.550 g of the crude product **18** as a pale yellow solid, which was used in the next step without any further purification. ^1^H NMR (300 MHz, CDCl3): *δ* (ppm) 6.89 (1H, d, *J* = 1.6 Hz, H-2′), 6.85 (1H, dd, *J* = 7.9, 1.6 Hz, H-6′), 6.73 (1H, d, *J* = 7.9 Hz, H-5′), 6.60 (1H, s, H-5), 5.90 (2H, s, H-7′), 3.82 (2H, s, H-14), 3.70 (3H, s, H-12), 3.25 (3H, s, H-13), 1.59 (9H, s, CH_3_ *t*-Bu); ^13^C NMR (75 MHz, CDCl3): *δ* (ppm) 161.6 (C=O Boc), 160.3 (C-11), 155.0 (C-9), 147.7 (C-3′), 146.9 (C-2), 146.1 (C-4′), 141.1 (C-7), 140.8 (C-4), 133.3 (C-1′), 121.8 (C-6′), 117.6 (C-5), 109.5 (C-2′), 108.2 (C-5′), 100.8 (C-7′), 81.5 (Cq *t*-Bu Boc), 34.8 (C-14), 32.1 (C-12), 28.8 (CH_3_ *t*-Bu BOC), 25.8 (C-13); HRESIMS *m*/*z* 441.1930 [M+H]^+^ (calcd for C_21_H_25_N_6_O_5_, 441.1886).

(b) The crude product **18** was dissolved in DCM (4.7 mL), trifluoroacetic acid (4.7 mL) was added and the resulting mixture was stirred at room temperature for 2 h. Then, the solvent was evaporated under reduced pressure, and the resulting product was dissolved in EtOAc and washed with a 10% aqueous solution of NaHCO_3_. The aqueous layer was extracted with EtOAc (3 × 10 mL), and the combined organic phases were washed with water and brine and then dried over MgSO_4_, filtered and concentrated. The crude product was purified by flash column chromatography on silica gel (gradient 100% DCM to 100% EtOAc) to give **4** (0.280 g, 64% yield) as a pale orange solid. ^1^H NMR (300 MHz, CDCl3): *δ* (ppm) 6.77 (1H, d, *J* = 7.9 Hz, H-5′), 6.74 (1H, d, *J* = 1.7 Hz, H-2′), 6.71 (1H, dd, *J* = 7.9, 1.7 Hz, H-6′), 6.41 (1H, s, H-5), 5.95 (2H, s, H-7′), 3.87 (2H, s, H-14), 3.69 (3H, s, H-12), 3.26 (3H, s, H-13); ^13^C NMR (75 MHz, CDCl3): *δ* (ppm) 163.2 (C-11), 157.2 (C-9), 147.7 (C-3′), 147.7 (C-2), 146.5 (C-4′), 139.7 (C-7), 134.7 (C-4), 131.6 (C-1′), 121.8 (C-6′), 115.9 (C-5), 109.2 (C-2′), 108.5 (C-5′), 101.0 (C-7′), 33.1 (C-14), 32.3 (C-12), 25.5 (C-13); HRESIMS *m*/*z* 341.1363 [M+H]^+^ (calcd for C_16_H_17_N_6_O_3_, 341.1362).

### 4.7. Complexation of Clathridine A (***3***) and Clathridimine (***4***)

Homodimeric (clathridine A)_2_ Zn^2+^ (**9**): Prepared according to the literature [22]. To a stirred solution of clathridine A (**3**) (5.3 mg, 0.015 mmol) in CH_2_Cl_2_ (10 mL) was added a 0.1 M aqueous ZnSO_4_ solution (10 mL). The reaction mixture was kept under stirring at room temperature for 1 h. The organic layer was separated, dried over anhydrous MgSO_4_ and concentrated to obtain **5** (5.6 mg) as a yellow solid, which was identical to natural **9** by comparison of their spectral and chromatographic data.

*Homodimeric (clathridimine)_2_ Zn^2+^* (**27**): To a stirred solution of clathridimine (**4**) (5.1 mg, 0.015 mmol) in CH_2_Cl_2_ (10 mL) was added a 0.1 M aqueous ZnSO_4_ solution (10 mL). The reaction mixture was kept under stirring at room temperature for 1 h. The organic layer was separated, dried over anhydrous MgSO_4_ and concentrated to give **27** (5.6 mg) as a yellow solid. ^1^H NMR (500 MHz, CDCl3): *δ* (ppm) 6.63 (2H, s, H-5), 6.49 (2H, d, *J* = 8.3 Hz, H-5′), 6.23 (2H, d, *J* = 3.9 Hz, H-6′), 6.22 (2H, s, H-2′), 5.90 (4H, dd, *J* = 15.4, 0.9 Hz, H-7′), 3.81 (6H, s, H-12), 3.51 (2H, d, *J* = 16.2 Hz, H-14a), 3.34 (2H, d, *J* = 16.2 Hz, H-14b), 3.16 (2H, s, H-13); ^13^C NMR (125 MHz, CDCl_3_): *δ* (ppm) 164.0 (C-9), 158.7 (C-11), 149.5 (C-2), 147.8 (C-3′), 146.3 (C-4′), 135.4 (C-4), 131.1 (C-1′), 121.2 (C-6′), 117.2 (C-5), 108.4 (C-6′), 107.9 (C-5′), 101.3 (C-7′), 33.3 (C-14), 32.7 (C-12), 25.6 (C-13); HRESIMS *m*/*z* 743.1780 [M + H]^+^ (calcd for C_32_H_31_N_12_O_6_Zn, 743.1781).

*Heterodimeric (clathridine A-clathridimine) Zn^2+^* (**10**): To a stirred solution of clathridine A (**3**) (5.3 mg, 0.015 mmol) and clathridimine (**4**) (5.1 mg, 0.015 mmol) in CH_2_Cl_2_ (20 mL) was added a 0.1 M aqueous ZnSO_4_ solution (20 mL). The reaction mixture was kept under stirring at room temperature for 1 h. The organic layer was separated, dried over anhydrous MgSO_4_ and concentrated to give a mixture of **9**, **10** and **27** (7.0 mg) as a yellow solid. NMR data: see Table 1; HRESIMS *m*/*z* 744.1639 [M+H]^+^ (calcd for C_32_H_30_N_11_O_7_Zn, 744.1621).

### 4.8. Total Synthesis of Leucettamine B (***5***)

*L-DOPA methyl ester* (**20**): To a solution of *L*-DOPA (5.0 g, 25 mmol, 1 eq.) in MeOH (30 mL) at 0 °C, SOCl_2_ (2.0 mL, 28 mmol, 1.1 eq.) was added dropwise. The reaction mixture was allowed to warm to room temperature and was then refluxed for 2 h. After completion of the reaction, the mixture was co-evaporated with toluene three times to give **20** as a white solid (6.8 g, 99%). ^1^H NMR (300 MHz, MeOD): *δ* (ppm) 6.77 (1H, d, *J* = 8.0 Hz, H-8), 6.69 (1H, d, *J* = 2.0 Hz, H-5), 6.57 (1H, dd, *J* = 8.0, 2.0 Hz, H-9), 4.22 (1H, t, *J* = 13.0, 6.8 Hz, H-2), 3.82 (3H, s, O-CH_3_), 3.08 (2H, ddd, *J* = 23.0, 13.0, 7.0, 5.9 Hz, H-3); ^13^C NMR (75 MHz, MeOD): *δ* (ppm) 170.6 (C-1), 146.9 (C-6), 146.3 (C-7), 126.4 (C-4), 122.0 (C-9), 117.5 (C-5), 117.0 (C-8), 55.5 (C-2), 53.7 (O-CH_3_), 36.9 (C-3); HRESIMS *m*/*z* 212.0910 [M+H]^+^ (calcd for C_10_H_14_NO_4_, 212.0923).

*N-Boc-L-DOPA methyl ester* (**21**): To a solution of *L*-DOPA methyl ester (6.3 g, 25.5 mmol, 1 eq.) in MeOH (130 mL), triethylamine (7.0 mL, 51 mmol, 2 eq.) was added, followed by the introduction of BOC_2_O (6.12 g, 28 mmol, 1.1 eq). The reaction mixture was stirred at room temperature for 3 h. Then, the solvent was evaporated under reduced pressure, and the resulting residue was neutralized with a solution of 1 M HCl (5 mL). The aqueous solution was extracted with EtOAc (3 × 10 mL), and the combined organic layers were dried over MgSO_4_, filtered and concentrated to give **21** as a white solid (7.4 g, 94%), which was used in the next step without further purification. ^1^H NMR (300 MHz, MeOD): *δ* (ppm) 6.68 (1H, d, *J* = 8.0 Hz, H-8), 6.63 (1H, d, *J* = 2.0 Hz, H-5), 6.50 (1H, dd, *J* = 8.0, 2.0 Hz, H-9), 4.28 (1H, dd, *J* = 13.6, 8.1, 6.2 Hz, H-2), 3.67 (3H, s, O-CH_3_), 2.92 (1H, dd, *J* = 19.8, 13.6, 6.2 Hz, H-3a), 2.77 (1H, dd, *J* = 21.7, 13.6, 8.1 Hz, H-3b), 1.39 (9H, s, CH_3_ BOC); ^13^C NMR (75 MHz, MeOD): *δ* (ppm) 174.5 (C-1), 157.8 (C=O BOC), 146.3 (C-6), 145.3 (C-7), 129.6 (C-4), 121.7 (C-9), 117.4 (C-5), 116.4 (C-8), 80.8 (Cq BOC), 56.8 (C-2), 52.7 (O-CH_3_), 38.3 (C-3), 28.7 (CH_3_ BOC); HRESIMS *m*/*z* 312.1462 [M+H]^+^ (calcd for C_15_H_22_NO_6_, 312.1447).

*Methyl-(S)-3-(benzo[d][1,3]dioxol-5-yl)-2-((tert-butoxycarbonyl)amino)propanoate* (**22**): To a solution of **21** (3.5 g, 11.5 mmol, 1 eq.) in anhydrous DMF (7 mL), cesium fluoride (8.7 g, 57 mmol, 5 eq.) was added, and the reaction mixture was stirred at room temperature for 1 h. Then, dichloromethane (3.5 mL, 52 mmol, 4.5 eq.) was added, and the mixture was stirred at 110 °C for 2 h. After returning to room temperature, the reaction mixture was diluted with Et_2_O and washed with a cold saturated solution of NaHCO_3_ (3 × 50 mL). The organic layers were dried over MgSO_4_, filtered and concentrated. The crude product was purified by flash column chromatography on silica gel (gradient 100% Heptane to 50% EtOAc) to give **22** (1.1 g, 30% yield) as a colorless oil. ^1^H NMR (300 MHz, CDCl_3_): *δ* (ppm) 6.72 (1H, d, *J* = 8.0 Hz, H-8), 6.59 (1H, d, *J* = 1.5 Hz, H-5), 6.55 (1H, dd, *J* = 8.0, 1.5 Hz, H-9), 5.92 (2H, s, H-10), 5.00 (1H, d, *J* = 7.0 Hz, NH-BOC), 4.51 (1H, dd, *J* = 13.5, 8.1, 6.0 Hz, H-2), 3.71 (3H, s, O-CH_3_), 2.98 (2H, m, H-3), 1.42 (9H, s, CH_3_ BOC); ^13^C NMR (75 MHz, CDCl_3_): *δ* (ppm) 174.5 (C-1), 157.8 (C=O BOC), 146.3 (C-6), 145.3 (C-7), 129.8 (C-4), 122.5 (C-9), 105.9 (C-5), 108.4 (C-8), 101.5 (C-10), 88.9 (Cq BOC), 53.6 (C-2, O-CH_3_), 38.1 (C-3), 28.3 (CH_3_ BOC); HRESIMS *m*/*z* 346.1255 [M+Na]^+^ (calcd for C_16_H_21_NO_6_Na, 346.1267).

*Methyl-3-(benzo[d][1,3]dioxol-5-yl)-2-((2,2,2-trifluoroacetyl)-λ4-azaneyl)propanoate* (**23**): To a solution of **22** (1.0 g, 3.3 mmol, 1 eq.) in dichloromethane (5.5 mL), trifluoroacetic acid (1.4 mL, C = 2.5 M) was added, and the reaction mixture was stirred at room temperature for 2 h. Then, the solvent was evaporated under reduced pressure, and the resulting residue was dissolved in EtOAc. After the addition of a few drops of Heptane, a precipitate appeared and was filtered. The obtained solid was dried to give **23** (0.97 g, 93% yield) as a white solid. ^1^H NMR (300 MHz, MeOD): *δ* (ppm) 6.80 (1H, d, *J* = 8.0 Hz, H-8), 6.75 (1H, d, *J* = 1.8 Hz, H-5), 6.70 (1H, dd, *J* = 8.0, 1.8 Hz, H-9), 5.95 (2H, s, H-10), 4.26 (1H, dd, *J* = 7.5, 5.8 Hz, H-2), 3.82 (3H, s, O-CH_3_), 3.19 (1H, dd, *J* = 14.5, 5.8 Hz, H-3a), 3.06 (1H, dd, *J* = 14.5, 7.5 Hz, H-3b); ^13^C NMR (75 MHz, MeOD): *δ* (ppm) 170.5 (C-1), 149.7 (C-6), 149.0 (C-7), 128.8 (C-4), 123.9 (C-9), 110.5 (C-5), 109.8 (C-8), 102.8 (C-10), 55.4 (C-2), 53.7 (O-CH_3_), 37.2 (C-3); HRESIMS *m*/*z* 224.0907 [M+H]^+^ (calcd for C_11_H_14_NO_4_, 224.0923).

*Pyr-DOPA-OMe* (**24**): To a solution of **23** (0.96 g, 3.0 mmol, 1 eq.) and trichloroacetyl pyrrole (0.7 g, 3.3 mmol, 1.1 eq.) in anhydrous acetonitrile (9 mL), triethylamine acid (1.0 mL, 6.9 mmol, 2.3 eq.) was added. The reaction mixture was stirred at room temperature for 60 h and then filtered and washed with acetonitrile. The solvent was evaporated under reduced pressure, and the resulting residue was dissolved in EtOAc (30 mL) and washed with water (30 mL). The aqueous layer was saturated with NaCl and extracted with EtOAc (3 × 30 mL). The combined organic layers were dried over MgSO_4_, filtered and concentrated. The crude product was purified by flash column chromatography on silica gel (gradient 100% Heptane to 100% EtOAc) to give **24** (0.95 g, 74% yield) as a yellow amorphous solid. ^1^H NMR (300 MHz, CDCl_3_): *δ* (ppm) 9.80 (1H, br s, NH pyrrole), 6.92 (1H, ddd, *J* = 4.0, 2.6, 1.4 Hz, H-2′), 6.72 (1H, d, *J* = 8.0 Hz, H-8), 6.62 (1H, d, *J* = 1.7 Hz, H-5), 6.58 (2H, m, H-9, H-4′), 6.41 (1H, d, *J* = 8.0 Hz, NH amide), 6.21 (1H, dt, *J* = 5.2, 2.6 Hz, H-3′), 5.92 (2H, s, H-10), 4.99 (1H, dt, *J* = 11.3, 5.6 Hz, H-2), 3.75 (3H, s, O-CH_3_), 3.12 (2H, ddd, *J* = 14.0, 5.6 Hz, H-3); ^13^C NMR (75 MHz, CDCl_3_): *δ* (ppm) 172.3 (C-1), 160.7 (C-6′), 148.1 (C-6), 147.0 (C-7), 129.6 (C-4), 125.5 (C-5′), 122.6 (C-9), 122.2 (C-2′), 110.1 (C-3′), 110.0 (C-4′), 109.7 (C-5), 108.5 (C-8), 101.2 (C-10), 53.3 (C-2), 52.6 (O-CH_3_), 38.1 (C-3); HRESIMS *m*/*z* 317.1132 [M+H]^+^ (calcd for C_16_H_17_N_2_O_5_, 317.1137).

*Diketopiperazine Pyr-DOPA* (**25**): Product **24** (0.300 g, 0.95 mmol, 1 eq.) was dissolved in degassed anhydrous THF (20 mL, C = 0.05 M), and the solution was cooled to 0 °C. NaH (95%, 0.034 g, 1.3 mmol, 1.4 eq.) was added, and the reaction mixture was stirred at this temperature for 5 min. Then, it was allowed to warm to room temperature and stirred for 1 h. After completion of the reaction, the reaction mixture was poured slowly into an acetate buffer (pH = 3.8, 30 mL), and the aqueous layer was gently washed with EtOAc (3 × 40 mL). The combined organic layers were dried over MgSO_4_, filtered and concentrated to give **25** (0.270 g, 98% yield) as a yellow amorphous solid, which was used in the next step without further purification. ^1^H NMR (300 MHz, CDCl_3_): *δ* (ppm) 7.52 (1H, dd, *J* = 1.52 Hz, H-2′), 7.06 (1H, dd, *J* = 1.52 Hz, H-4′), 6.74 (1H, d, *J* = 7.8 Hz, H-8), 6.69 (1H, d, *J* = 1.5 Hz, H-5), 6.66 (1H, dd, *J* = 7.8, 1.5 Hz, H-9), 6.50 (1H, t, *J* = 3.35 Hz, H-3′), 6.30 (1H, br s, NH amide), 5.92 (2H, dd, *J* = 2.6, 1.3 Hz, H-10), 4.56 (1H, ddd, *J* = 8.8, 3.8, 1.9 Hz, H-2), 3.40 (1H, dd, *J* = 13.8, 3.8 Hz, H-3a), 3.00 (1H, dd, *J* = 13.8, 8.8 Hz, H-3b); ^13^C NMR (75 MHz, CDCl_3_): *δ* (ppm) ND (C-1), ND (C-6′), 148.4 (C-6), 147.5 (C-7), 127.9 (C-4), 125.2 (C-5′), 122.6 (C-9), 119.9 (C-2′), 119.1 (C-4′), 115.8 (C-3′), 109.7 (C-5), 109.0 (C-8), 101.4 (C-10), 58.9 (C-2), 41.0 (C-3); HRESIMS *m*/*z* 285.0851 [M+H]^+^ (calcd for C_15_H_13_N_2_O_4_, 285.0875).

*Leucettamine B* (**5**) *and its regioisomer* **26**: To a solution of diketopiperazine **25** (0.206 g, 0.73 mmol, 1 eq.) in anhydrous DMF (10 mL, C = 0.08 M), diethyl sulfide (0.31 mL, 2.9 mmol, 4 eq.) was added, and the reaction mixture was stirred under O_2_ atmosphere at room temperature for 48 h. After the formation of diketopiperazine-OH, methylguanidine hydrochloride (0.090 g, 0.8 mmol, 1.1 eq.) and potassium *tert*-butanolate (0.100 g, 0.87 mmol, 1.2 eq.) were added, and the reaction mixture was stirred at room temperature for 30 min before heating to 60 °C for 24 h. After returning to room temperature, the mixture was co-evaporated with toluene three times. The crude product was purified by flash column chromatography on silica gel (gradient 100% DCM to 8:20 DCM/MeOH) to give 4 fractions, which were further purified by reverse-phase HPLC (column: Waters Sunfire C_18_, 10 mm × 150 mm, 5 μm; H_2_O + 0.1% formic acid/CH_3_CN + 0.1% formic acid, 90:10 to 60:40) to give leucettamine B (28 mg, 12% yield) and its regioisomer **26** (11 mg, 5%).

*Leucettamine B* (**5**): ^1^H NMR (300 MHz, CDCl_3_): *δ* (ppm) 8.04 (d, *J* = 1.5 Hz, 1H, H-9), 7.33 (dd, *J* = 8.1, 1.5 Hz, 1H, H-13), 6.82 (d, *J* = 8.1 Hz, 1H, H-12), 6.43 (s, 1H, H-7), 6.01 (s, 2H, H-14), 3.15 (s, 3H, 3- N-CH_3_); ^13^C NMR (75 MHz, CDCl_3_): *δ* (ppm) 170.6 (C-4), 160.4 (C-2), 148.9 (C-10), 147.9 (C-11), 132.0 (C-6), 126.4 (C-13), 126.1 (C-5), 114.8 (C-7), 110.9 (C-9), 108.9 (C-12), 102.2 (C-14), 25.8 (3-N-CH_3_); HRESIMS *m*/*z* 246.0870 [M+H]^+^ (calcd for C_12_H_12_N_3_O_3_, 246.0879).

*Regioisomer* **26**: ^1^H NMR (300 MHz, CDCl_3_): *δ* (ppm) 8.04 (d, *J* = 1.5 Hz, 1H, H-9), 7.33 (dd, *J* = 8.1, 1.5 Hz, 1H, H-13), 6.82 (d, *J* = 8.1 Hz, 1H, H-12), 6.34 (s, 1H, H-7), 6.01 (s, 2H, H-14), 3.05 (s, 3H, 3- N-CH_3_); HRESIMS *m*/*z* 246.0858 [M+H]^+^ (calcd for C_12_H_2_N_3_O_3_, 246.0879).

### 4.9. Biological Screening

hCol X–promoter activity in a murine ATDC5 pre-chondrocyte line cultured under endochondral differentiation conditions was assessed by the measurement of luminometric luciferase signals. The human Col X promoter–luciferase reporter plasmid (Col X–Luc) was constructed by cloning the full promoter of the human type X collagen gene into pMet-Luc2. A stably transfected monoclonal cell line resistant to neomycin (G418) was created by transfecting Col X–Luc into the mouse chondrogenic cell line (ATDC5).

The expression of type X collagen is confined within hypertrophic chondrocytes and precedes the embark of endochondral bone formation [17]. Type X collagen facilitates endochondral ossification by regulating matrix mineralization and compartmentalizing matrix components.

The stimulation of Col X transcription means the stimulation of endochondral ossification, involved in bone repair. Conversely, the reduction and the inhibition of Col X transcription can be of interest to stop the final steps of endochondral ossification during the chondrogenic differentiation of mesenchymal stem cells [42].

### 4.10. ATDC5 Micromass Model

Pre-chondrogenic ATDC5 cells derived from mouse tetracarcinoma (AT805) were cultured in growth medium (1:1 Dulbecco’s modified Eagle’s medium (DMEM):Ham’s F-12 mix (Gibco)) containing 1% (*vol*/*vol*) antibiotic–antimycotic (Gibco), 5% fetal bovine serum (FBS) (Gibco), 10 μg/mL human transferrin (Sigma-Aldrich products, France) and 30 nM sodium selenite (Sigma). Cells were maintained in a humidified atmosphere of 5% CO_2_ at 37 °C.

High-density micromass cultures of ATDC5 cells were grown to study endochondral differentiation. Cells were trypsinised, washed and resuspended at 2 × 10^7^ cells/mL in a chondrogenic medium made of DMEM-F12 enriched by 1% (*vol*/*vol*) antibiotic–antimycotic, 5% FBS and 1× of ITS (insuline, transferin and selenite) premix (resulting in 10 μg/mL insulin, 5 μg/mL human transferrin and 30 nM sodium selenite) (Life Technologies). Three droplets of 10^6^ cells (10 μL each) were carefully placed in the center of each well of a 6-well plate. Cells were allowed to adhere for 2 h at 37 °C, followed by the addition of 500 μL chondrogenic medium. Hypertrophic differentiation and mineralization were induced by a mineralization medium made of α-minimum essential medium Eagle (Gibco) containing 1% (*vol*/*vol*) antibiotic–antimycotic, 5% FBS, 1× of ITS premix, 50 μg/mL ascorbic acid-2-phosphate (Sigma) and 10 mM β-glycerophosphate (Sigma). Micromasses were collected at time points 7, 14 and 21 days. Each time point was processed with three technical replicates.

Matrix mineralization was assessed by Alizarin red staining. Micromasses were treated with 100 μg/mL of compound and cultured in osteogenic differentiation medium for 7, 14 and 21 days. Cells were then fixed with 4% paraformaldehyde for 30 min at 4 °C and incubated with 1% (*w*/*v*) Alizarin red solution (Sigma-Aldrich products, France, A5533) for 5 min. Staining was observed under a light microscope (ZEISS axiovert).

For Alizarin red quantification, 10% acetic acid was added to the cells and incubated for 30 min at room temperature. Cells were gently scraped. Lysates were heated at 85 °C for 10 min and centrifuged for 15 min at 4 °C. The supernatant was recovered and treated with 10% ammonium hydroxide, and the absorbance representative of a mineralized nodule formation was measured at 425 nm.

### 4.11. DFT Calculations

The geometries of all intermediate structures involved in the hydrolysis mechanism of clathridimine were optimized in the gas phase using the Gaussian 09 package [43] with Becke’s three-parameter hybrid exchange functional (B3LYP) [44,45] and the 6–31G(d,p) basis set. Subsequent vibrational frequency calculations confirmed that these conformations were local minima. The complete characterization of these structures can be found in the Supplementary Materials (Figures S49 and S50).

## Figures and Tables

**Table 1:** ^1^H and ^13^C NMR (500 MHz, CDCl_3_) shifts of the three complexes **9**, **10** and **27**.

	Clathridine Zinc Complex (9)		Clathridimine Zinc Complex (27)		Clathridine–Clathridimine Heterodimeric Zinc Complex (10)	
Position	*δ*_C_, Type	*δ*_H_ Mult, (*J* in Hz)	*δ*_C_, Type	*δ*_H_ Mult, (*J* in Hz)	*δ*_C_, Type	*δ*_H_ Mult, (*J* in Hz)
2	149.0, C ^1^	-	149.5, C ^1^	-	149.0 ^3^, 149.5 ^4^	-
4	136.3, C ^1^	-	135.4, C ^1^	-	136.3 ^3^, 135.4 ^4^	-
5	117.7, CH	6.63, s	117.2, CH	6.63, s	117.7 ^3^, 117.2 ^4^	6.63, s
7	nd	-	nd	-	nd	-
9	161.4, C ^1^	-	158.7, C ^1^	-	161.2 ^3^, 159.0 ^4^	-
11	164.9, C ^1^	-	164.0, C ^1^	-	164.9 ^3^, 164.0 ^4^	-
12	32.7, CH_3_	3.80, s	32.7, CH_3_	3.81, s	32.7, CH_3_	3.80, s
13	24.7, CH_3_	3.04, s	25.6, CH_3_	3.16, s	24.7, CH_3_	**3.14 ^4^, 3.05 ^3^, s ^2^**
14a	33.30, CH_2_	3.51, d (16.3)	33.30, CH_2_	3.51, d (16.2)	33.27, CH_2_	3.51, d (16.3)
14b		3.37, d (16.3)		3.34, d (16.2)		**3.35, d (16.3) ^2^**
1′	131.1, C ^1^	-	131.0, C ^1^	-	131.0, C ^1^	-
2′	108.6, CH	6.25, s	108.4, CH	6.22, s	108.5, CH	6.24, s
3′	147.7, C ^1^	-	147.8, C ^1^	-	147.7, C ^1^	-
4′	146.5, C ^1^	-	146.3, C ^1^	-	146.4, C ^1^	-
5′	107.9, CH	6.502, d (7.9)	107.9, CH	6.492, d (8.3)	107.8, CH	**6.499, d (7.9) ^2^**
6′	121.7, CH	6.26, d (7.9)	121.2, CH	6.23, d (8.3)	121.2, CH	6.24, m
7′	101.2, CH_2_	5.88, dd (13.6, 1.1)	101.3, CH_2_	5.90, dd (15.4, 0.9)	101.3, CH_2_	5.89, dd (13.8, 1.3)

^1^ Observed in HMBC NMR spectra; ^2^ difference clearly visible in the mixture; ^3^ Clathridine A; ^4^ Clathridimine.

## Data Availability

Data are contained within the article.

## Supplementary Materials

The following are available online at https://www.mdpi.com/article/10.3390/md22050196/s1: Spectroscopic data; copies of the 1H, 13C and NMR spectra; HPLC chromatograms and biological screening profiles. Figures S0–S50: Tables S1 and S2 and the single-crystal X-ray crystallography (SC-XRD) for the crystal structure determination of homodimeric (clathridine A)2 Zn2+ (**9**) [46,47,48,49,50].
