# Supplementary material for: Chemical Investigation of the Calcareous Marine Sponge Pericharax heteroraphis, Clathridine-A Related Derivatives Isolation, Synthesis and Osteogenic Activity
Source: Mar Drugs. 2024 Apr 25;22(5):196. doi: 10.3390/md22050196 (PMC11123192; doi:10.3390/md22050196)

Supporting information

Chemical Investigation of the Calcareous Marine Sponge *Pericharax heteroraphis*, Clathridine-A Related Derivatives Isolation, Synthesis and Osteogenic Activity

Capucine Jourdain de Muizon [1], Céline Moriou [1], Marceau Levasseur [1], David Touboul[1,2], Bogdan I. Iorga [1], Hristo Nedev[1], Elsa Van Elslande [1], Pascal Retailleau [1], Sylvain Petek [3], Eric Folcher [4], Arnaud Bianchi [5], Mireille Thomas[6], Solène Viallon[6], Sylvie Peyroche[6], Sarah Nahle[6], Marthe Rousseau* [6,7] and Ali Al-Mourabit*[1]

1 CNRS, Institut de Chimie des Substances Naturelles, Université Paris-Saclay, F-91190 Gif-sur-Yvette, France; capucine.jourdain@cnrs.fr (C.J.M.); celine.moriou@cnrs.fr (C.M.); marceau.levasseur (M.L.); david.touboul@cnrs.fr (D.T.); bogdan.iorga@cnrs.fr (B.I.I.); hristo.nedev@cnrs.fr (H.N.); elsa.van-elslande@cnrs.fr; pascal.retailleau@cnrs.fr (P.R.); ali.almourabit@cnrs.fr (A.A.)

2 Laboratoire de Chimie Moléculaire (LCM), CNRS, École polytechnique, Institut Polytechnique de Paris, 91120 Palaiseau, France

3 IRD, CNRS, Ifremer, Univ Brest, F-29280 Plouzane, France; sylvain.petek@ird.fr (S.P.)

4 IRD, SEOH, BPA5, F-98848 Nouméa, New Caledonia; eric.folcher@ird.fr (E.F.)

5 UMR 7365 CNRS-Université de Lorraine; arnaud.bianchi@univ-lorraine.fr (A.B.)

6 Université Jean Monnet Saint-Etienne, INSERM, Mines Saint Etienne, SAINBIOSE U1059, F-42023, Saint-Etienne, France; mireille.thomas@univ-st-etienne.fr (M.T.), sylvie.peyroche@univ-st-etienne.fr (S.P.), solene.viallon@univ-st-etienne.fr (S.V.), sarah.nahle@univ-st-etienne.fr (S.N.), marthe.rousseau@univ-st-etienne.fr (M.R.)

7 UMR5510 Mateis, CNRS, University of Lyon, INSA-Lyon, Lyon, France

* Correspondence: ali.almourabit@cnrs.fr (A.A.-M.); +33-169-824-585

Table of Contents

Table 1. Queensland Museum (QM) accession numbers, species and corresponding OTUs. 4

Figure S0: Isolated natural products. 5

Figure S1. ^{1}H NMR spectrum of natural clathridine A (**3**) in $CDCl_3$ (500 MHz). 6

Figure S2. ^{1}H NMR spectrum of synthetic preclathridine A (**15**) in $CDCl_3$ (300 MHz). 7

Figure S3. ^{13}C NMR spectrum of synthetic preclathridine A (**15**) in $CDCl_3$ (75 MHz). 8

Figure S4. ^{1}H NMR spectrum of synthetic clathridine A (**3**) in $CDCl_3$ (300 MHz). 9

Figure S5. ^{13}C NMR spectrum of synthetic clathridine A (**3**) in $CDCl_3$ (75 MHz). 10

Figure S6. ^{1}H-^{13}C HMBC NMR spectrum of synthetic clathridine A (**3**) in $CDCl_3$ (300 MHz). 11

Figure S7. ^{1}H NMR spectrum of compound **18** in $CDCl_3$ (300 MHz). 12

Figure S8. ^{13}C NMR spectrum of compound **18** in $CDCl_3$ (75 MHz). 13

Figure S9. ^{1}H NMR spectrum of compound **19** in $CDCl_3$ (500 MHz). 14

Figure S10. ^{13}C NMR spectrum of compound **19** in $CDCl_3$ (75 MHz). 15

Figure S11. ^{1}H NMR spectrum of compound **20** in $CDCl_3$ (500 MHz). 16

Figure S12. ^{13}C NMR spectrum of compound **20** in $CDCl_3$ (75 MHz). 17

Figure S13. ^{1}H NMR spectrum of synthetic clathridimine (**4**) in $CDCl_3$ (300 MHz). 18

Figure S14. ^{13}C NMR spectrum of synthetic clathridimine (**4**) in $CDCl_3$ (75 MHz). 19

Figure S15. ^{1}H-^{13}C HMBC NMR spectrum of synthetic clathridimine (**4**) in $CDCl_3$ (300 MHz). 20

Figure S16. ^{1}H NMR spectrum of compound **20** in CD_3OD (300 MHz). 21

Figure S17. ^{13}C NMR spectrum of compound **20** in CD_3OD (75 MHz). 22

Figure S18. ^{1}H NMR spectrum of compound **21** in CD_3OD (300 MHz). 23

Figure S19. ^{13}C NMR spectrum of compound **21** in CD_3OD (75 MHz). 24

Figure S20. ^{1}H NMR spectrum of compound **22** in $CDCl_3$ (300 MHz). 25

Figure S21. ^{13}C NMR spectrum of compound **22** in $CDCl_3$ (75 MHz). 26

Figure S22. ^{1}H NMR spectrum of compound **23** in CD_3OD (300 MHz). 27

Figure S23. ^{13}C NMR spectrum of compound **23** in CD_3OD (75 MHz). 28

Figure S24. ^{1}H NMR spectrum of compound **24** in $CDCl_3$ (300 MHz). 29

Figure S25. ^{13}C NMR spectrum of compound **24** in $CDCl_3$ (75 MHz). 30

Figure S26. ^{1}H NMR spectrum of compound **25** in $CDCl_3$ (300 MHz). 31

Figure S27. ^{13}C NMR spectrum of compound **25** in $CDCl_3$ (75 MHz). 32

Figure S28. ^{1}H NMR spectrum of compound **26** in Acetone-d_6 (300 MHz). 33

Figure S29. ^{1}H NMR spectrum of leucettamine B (**5**) in Acetone-d_6 (300 MHz). 34

Figure S30. ^{13}C NMR spectrum of leucettamine B (**5**) in Acetone-d_6 (75 MHz). 35

Figure S31. ^{1}H-^{13}C HMBC NMR spectrum of leucettamine B (**5**) in Acetone-d_6 (75 MHz). 36

Figure S32. ^{1}H NMR spectrum of natural homodimeric (clathridine A)$_2$ Zn^{2+} (**9**) in $CDCl_3$ (500 MHz). 37

Figure S33. ^{1}H-^{13}C HSQC NMR spectrum of natural homodimeric (clathridine A)$_2$ Zn^{2+} (**9**) in $CDCl_3$ (500 MHz). 38

Figure S34. ^{1}H-^{13}C HMBC NMR spectrum of natural homodimeric (clathridine A)$_2$ Zn^{2+} (**9**) in $CDCl_3$ (500 MHz). 39

Figure S35. ^{1}H NMR spectrum of synthetic homodimeric (clathridine A)$_2$ Zn^{2+} (**9**) in $CDCl_3$ (500 MHz). 40

Figure S36. ^{13}C NMR spectrum of synthetic homodimeric (clathridine A)$_2$ Zn^{2+} (**9**) in $CDCl_3$ (125 MHz). 41

Figure S37. HR-ESI mass spectrum of the synthetic homodimeric (clathridine A)$_2$ Zn^{2+} (**9**). 42

Figure S38. ^{1}H NMR spectrum of synthetic heterodimeric (clathridine A-clathridimine) Zn^{2+} (**10**) in $CDCl_3$ (500 MHz). 43

Figure S39. ^{13}C NMR spectrum of synthetic heterodimeric (clathridine A-clathridimine) Zn^{2+} (**10**) in $CDCl_3$ (125 MHz). 44

Figure S40. HR-ESI mass spectrum of the synthetic heterodimeric (clathridine A-clathridimine) Zn^{2+} (**10**). 45

Figure S41. ^{1}H NMR spectrum of synthetic homodimeric (clathridimine)$_2$ Zn^{2+} (**27**) in $CDCl_3$ (500 MHz). 46

Figure S42. ^{13}C NMR spectrum of synthetic homodimeric (clathridimine)$_2$ Zn^{2+} (**27**) in $CDCl_3$ (125 MHz). 47

Figure S43. ^{1}H-^{13}C HMBC NMR spectrum of synthetic homodimeric (clathridimine)$_2$ Zn^{2+} (**27**) in $CDCl_3$ (125 MHz). 48

Figure S44. HR-ESI mass spectrum of the synthetic homodimeric (clathridimine)$_2$ Zn^{2+} (**27**). 49

Figure S45: LC-MS profiles of the synthetic mixture of complexes (blue) and the sponge crude extract (red), indicating the detection of the dimeric complexes including the minor heterodimeric complex **10**. 50

Figure S46: Superposition of the 1H NMR spectra of homodimeric (clathridine A)2Zn2+ (**9**) (green), homodimeric (clathridimine)2 Zn2+ (**27**) (red) and heterodimeric (clathridine A-clathridimine) Zn2+ (**10**) (blue). 50

Figure S47: Zoom on the superposition of the 1H NMR spectra between 3 and 4 ppm, of homodimeric (clathridine A)2Zn2+ (**9**) (green), homodimeric (clathridimine)2 Zn2+ (**27**) (red) and heterodimeric (clathridine A-clathridimine) Zn2+ (**10**) (blue). 51

Single Crystal X-ray Crystallography (SC-XRD) 52

Table 2: Crystal data and structure refinement 53

Figure S48: (left) ORTEP drawing of homodimeric (clathridine A)$_2$ Zn^{2+} (**9**) with thermal ellipsoids drawn at the 50% probability level; (right) Labelling scheme of the structure. 54

Figure S49: Energy diagram of intermediates involved in the hydrolysis reaction with one water molecule. 55

Figure S50: Energy diagram of intermediates involved in the hydrolysis reaction considering two water molecules. 56

Table 1. Queensland Museum (QM) accession numbers, species and corresponding OTUs.

Reference	QM Registration	Genus species	OTU
P559	G339004	Petrosia sp.	QM2721
P560	G339005	Cinachyrella sp.	QM4680
P561	G339006	Echinodictyum asperum	QM0133
P562	G339007	Suberea laboutei	QM1511
P563	G339008	Stylissa cf. carteri	QM0336
P564	G339009	Myrmekioderma sp.	QM4997
P571	G339016	Dendrilla sp.	QM2575
P572	G339017	Dysidea lizardensis sp.	QM1519
P573	G339018	Aplysilla sp.	QM2034
P574	G339019	Haliclona (Haliclona) sp.	QM4999
P575	G339020	Haliclona (Haliclona) sp.	QM4499
P579	G339024	Stylissa massa	QM0925
P581	G339026	Hyrtios cf. erectus	QM0796
P582	G339027	Pericharax heteroraphis	QM0668
P586	G339031	Acanthodendrilla cf. 1948	QM1948
P587	G339032	Hyrtios erectus	QM0796
P588	G339033	Axinyssa sp.	QM3251
P589	G339034	Oscarella sp.	
P590	G339035	Fascaplysinopsis sp.	QM1549
P593	G339038	Leiosella sp.	QM6001
P594	G339039	Hyrtios erectus	QM0796
P595	G339040	Hyrtios erectus	QM0796
P596	G339041	Psammocina sp.	QM1944
P597	G339042	Rhaphoxya pallida	QM0465
P600	G339045	Jaspis sp.	QM4187
P601	G339046	Stylissa cf. carteri	QM0922
P602	G339047	Haliclona (Haliclona) sp.	QM1971
P603	G339048	Pseudoceratina sp.	QM1947
P605	G339050	Leucetta chagosensis	QM1402
P607	G339052	Halichondrida (Halichondrida) sp.	QM1429
P608	G339053	Ircinia sp.	QM1244
P609	G339054	Pericharax sp.	QM1361 or QM2065
P616	G339061	Astrosclera willeyana	QM0656
P618	G339063	Pericharax sp.	QM2116
P620	G339065	Petrosia sp.	QM4179
P621	G339066	Petrosia sp.	QM2035
P622	G339067	Lissodendoryx (Ectydoryx) sp.	QM1281
P625	G339070	Fascaplysinopsis sp.	QM6004
P628	G339073	Stylissa cf. carteri	QM0922
P633	G339078	Dysidea cf. pallescens sp.	QM0630
P634	G339079	Halichondrida (Halichondrida) sp.	QM1984
P636	G339081	Suberea sp.	QM2121
P640	G339085	Ircinia sp.	QM2707
P642	G339087	Cacospongia sp.	QM6009
P646	G339091	Fascaplysinopsis sp.	
P658	G339103	Chelonaplysilla delicata	QM1829
P660	G339105	Petrosia sp.	QM1895

Figure S0: Isolated natural products.

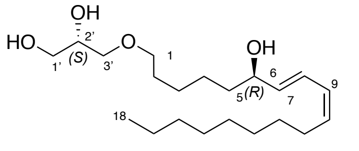

(2'*S*, 6*R*) pericharaxin A (**1**)

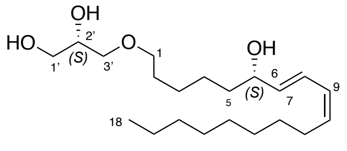

(2'*S*, 6*S*) pericharaxin B (**2**)

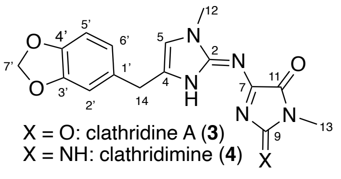

X = O: clathridine A (**3**)
X = NH: clathridimine (**4**)

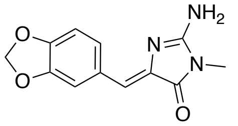

leucettamine B (**5**)

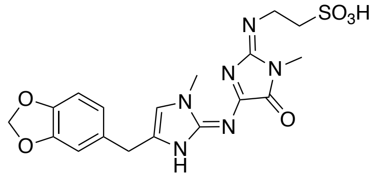

(9*E*)-clathridine-9-N-(2-sulfoethyl)imine (**6**)

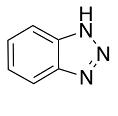

benzotriazole, (BTZ , **7**)

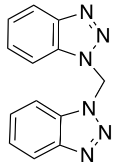

bis-BTZ-methane (**8**)

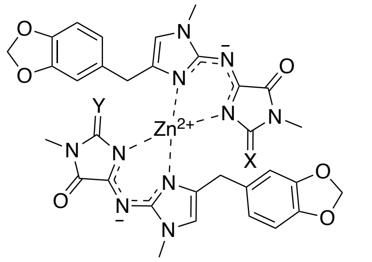

X = Y = O: homodimeric (clathridine A)$_2$Zn^{2+} (**9**)
X = O, Y = NH: heterodimeric (clathridine A-clathridimine)Zn^{2+} (**10**)

Figure S1. ^{1}H NMR spectrum of natural clathridine A (**3**) in $CDCl_3$ (500 MHz).

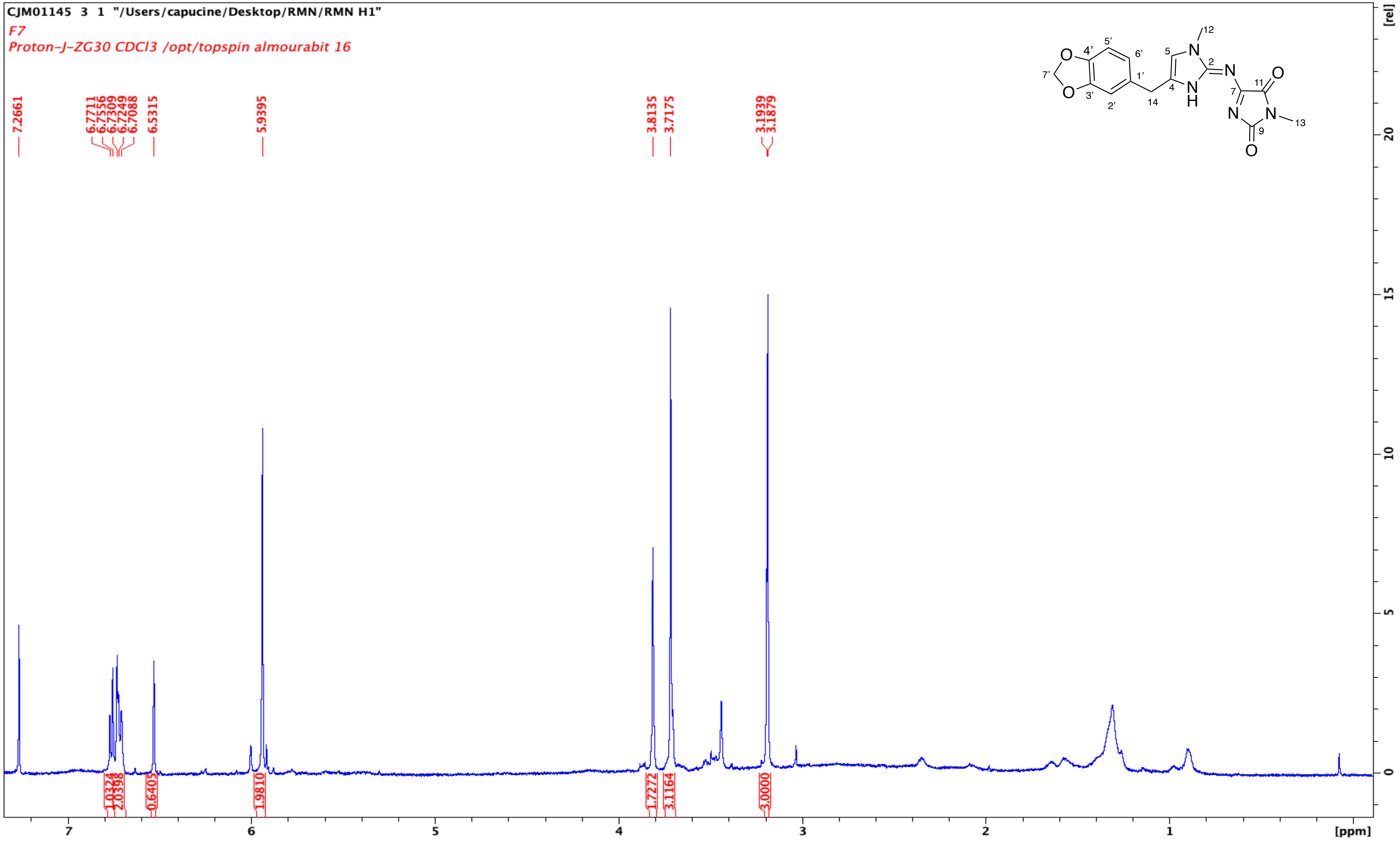

Figure S2. ^{1}H NMR spectrum of synthetic preclathridine A (**15**) in $CDCl_3$ (300 MHz).

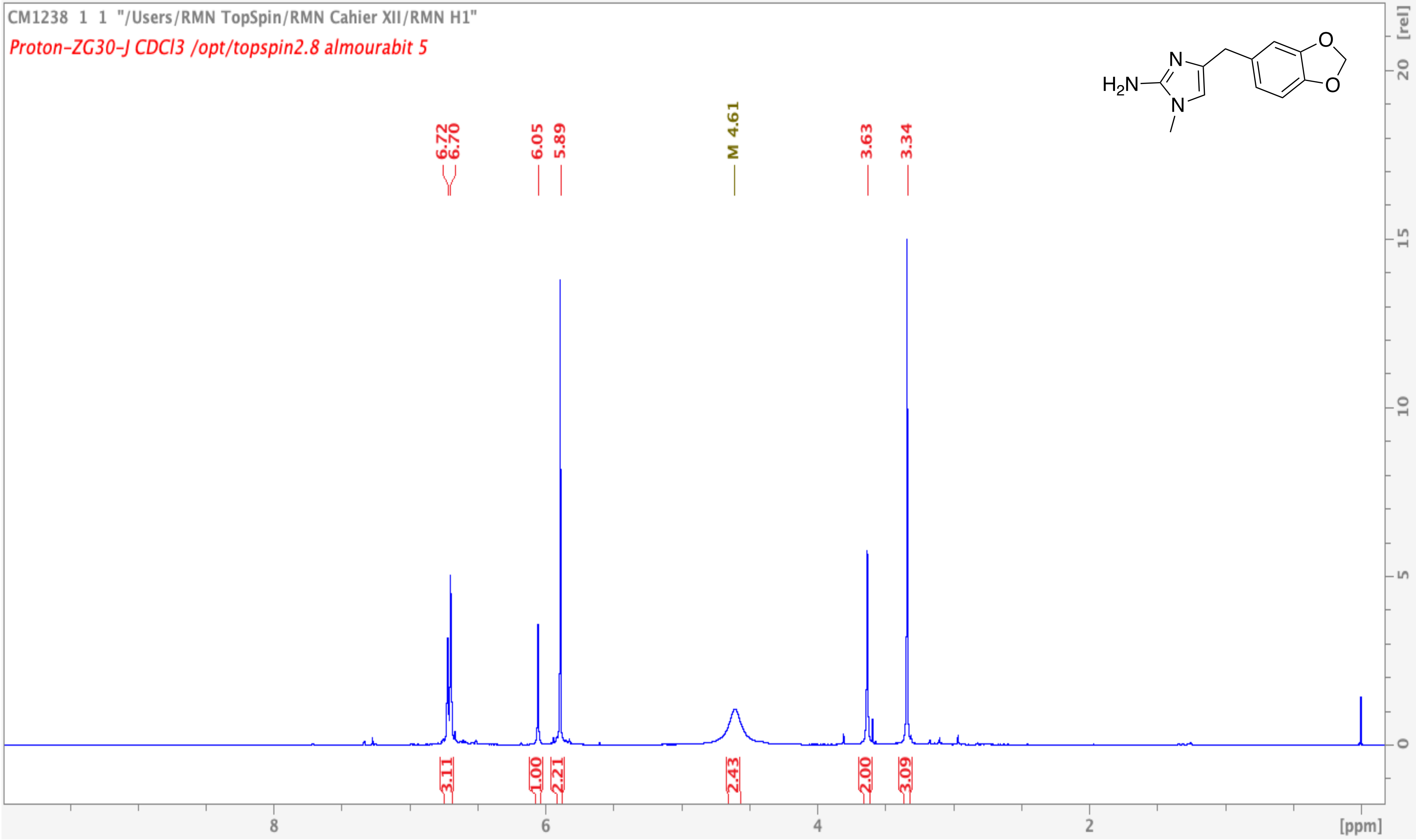

Figure S3. ^{13}C NMR spectrum of synthetic preclathridine A (**15**) in $CDCl_3$ (75 MHz).

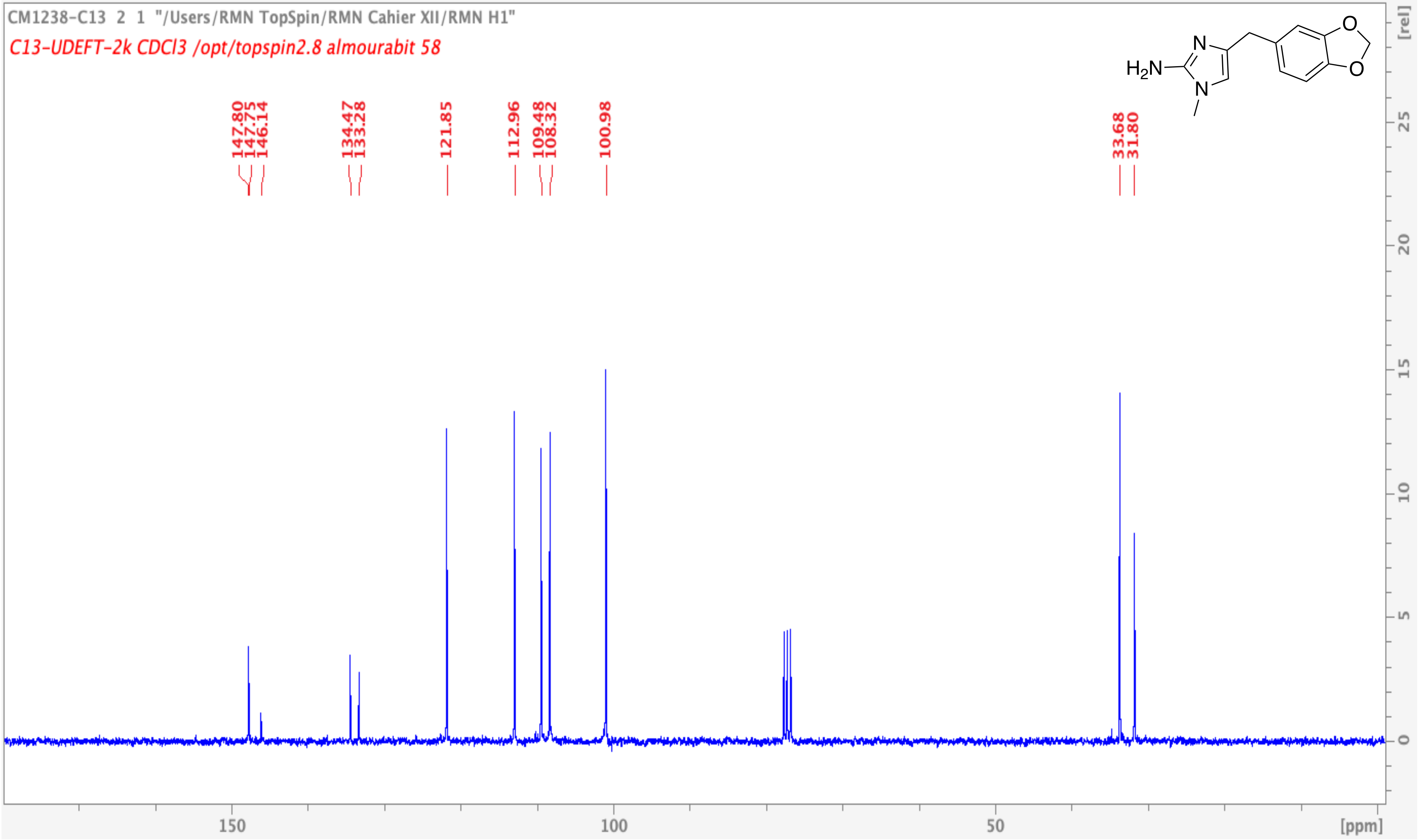

Figure S4. ^{1}H NMR spectrum of synthetic clathridine A (**3**) in $CDCl_3$ (300 MHz).

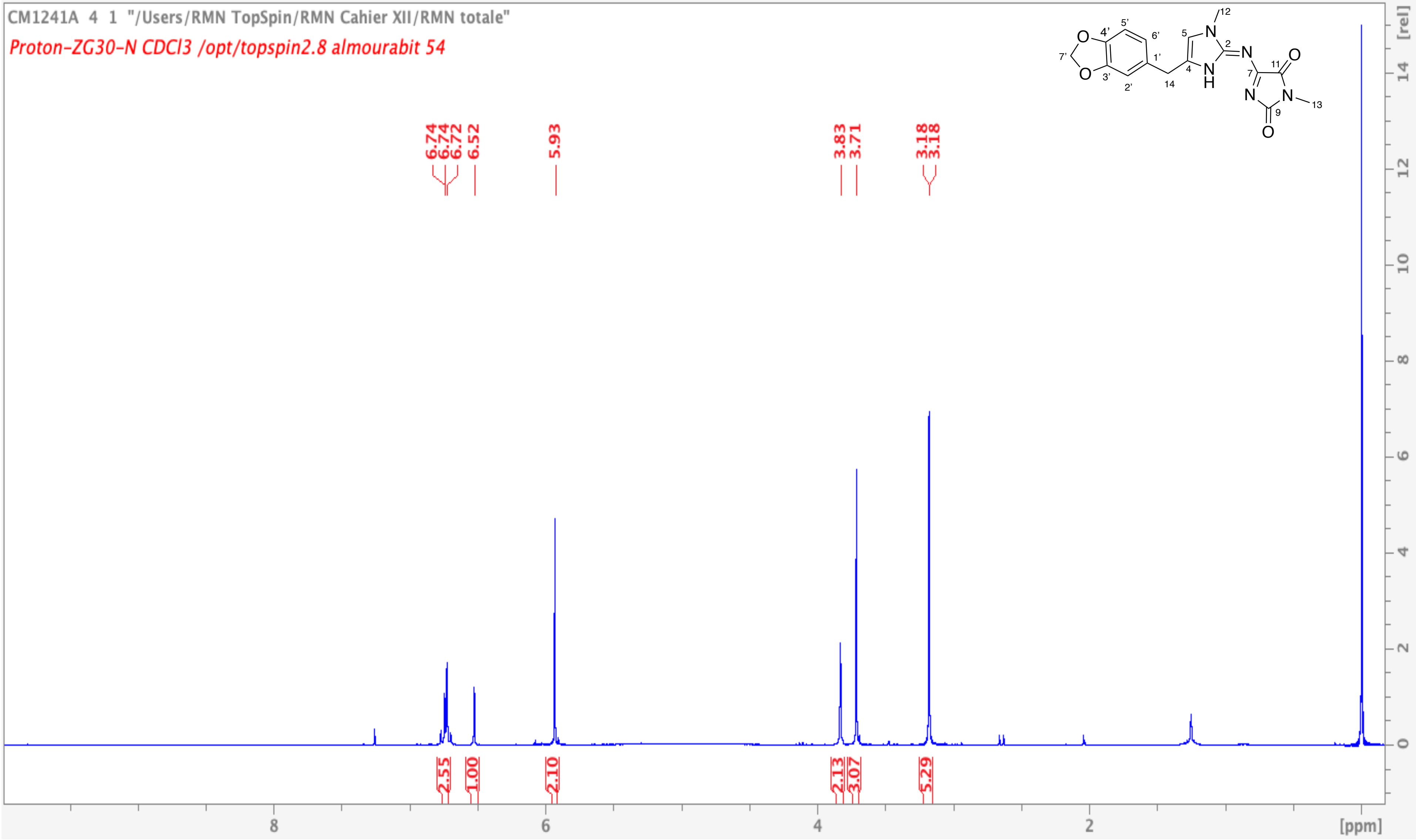

Figure S5. ^{13}C NMR spectrum of synthetic clathridine A (**3**) in $CDCl_3$ (75 MHz).

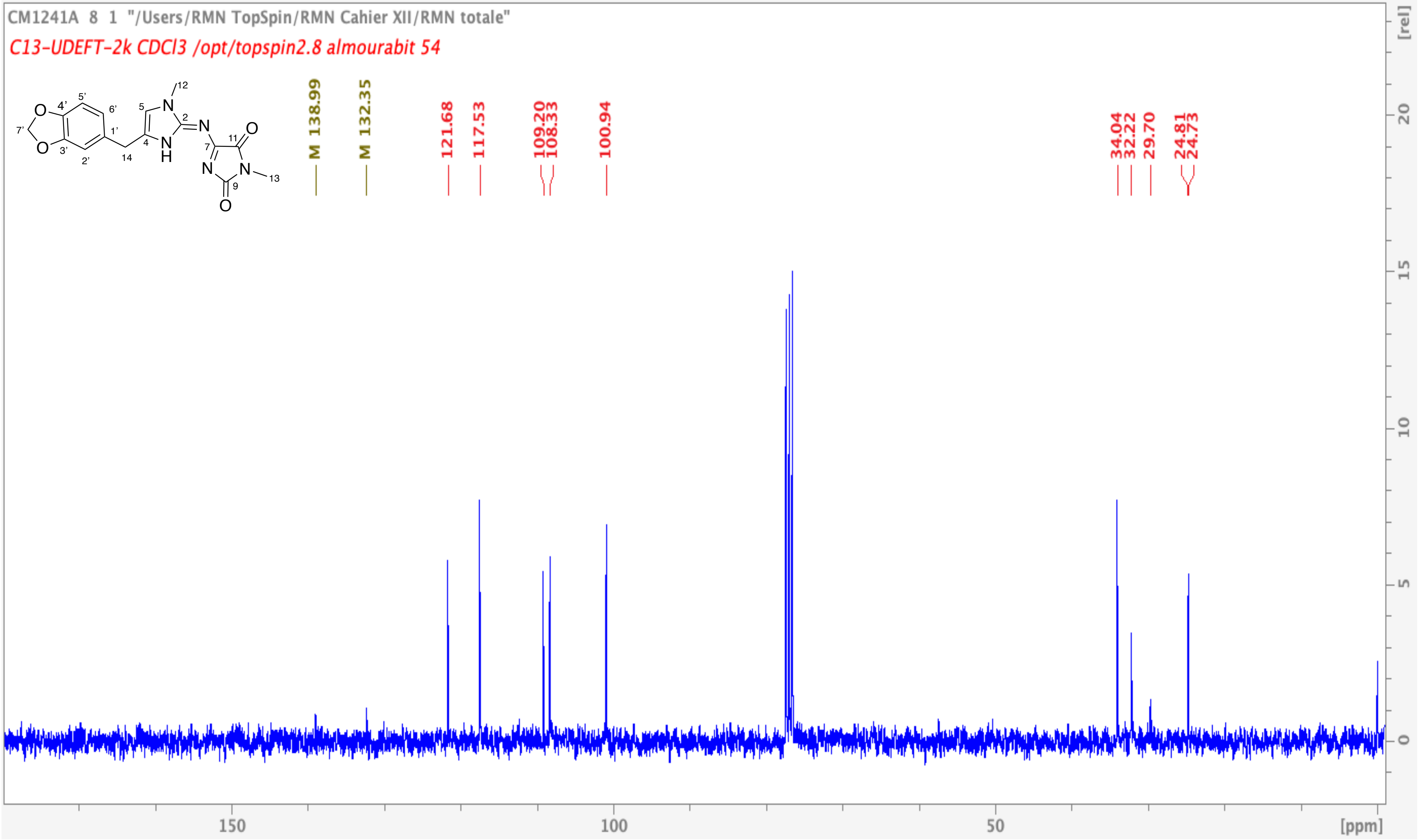

Figure S6. ^1H-^{13}C HMBC NMR spectrum of synthetic clathridine A (**3**) in $CDCl_3$ (300 MHz).

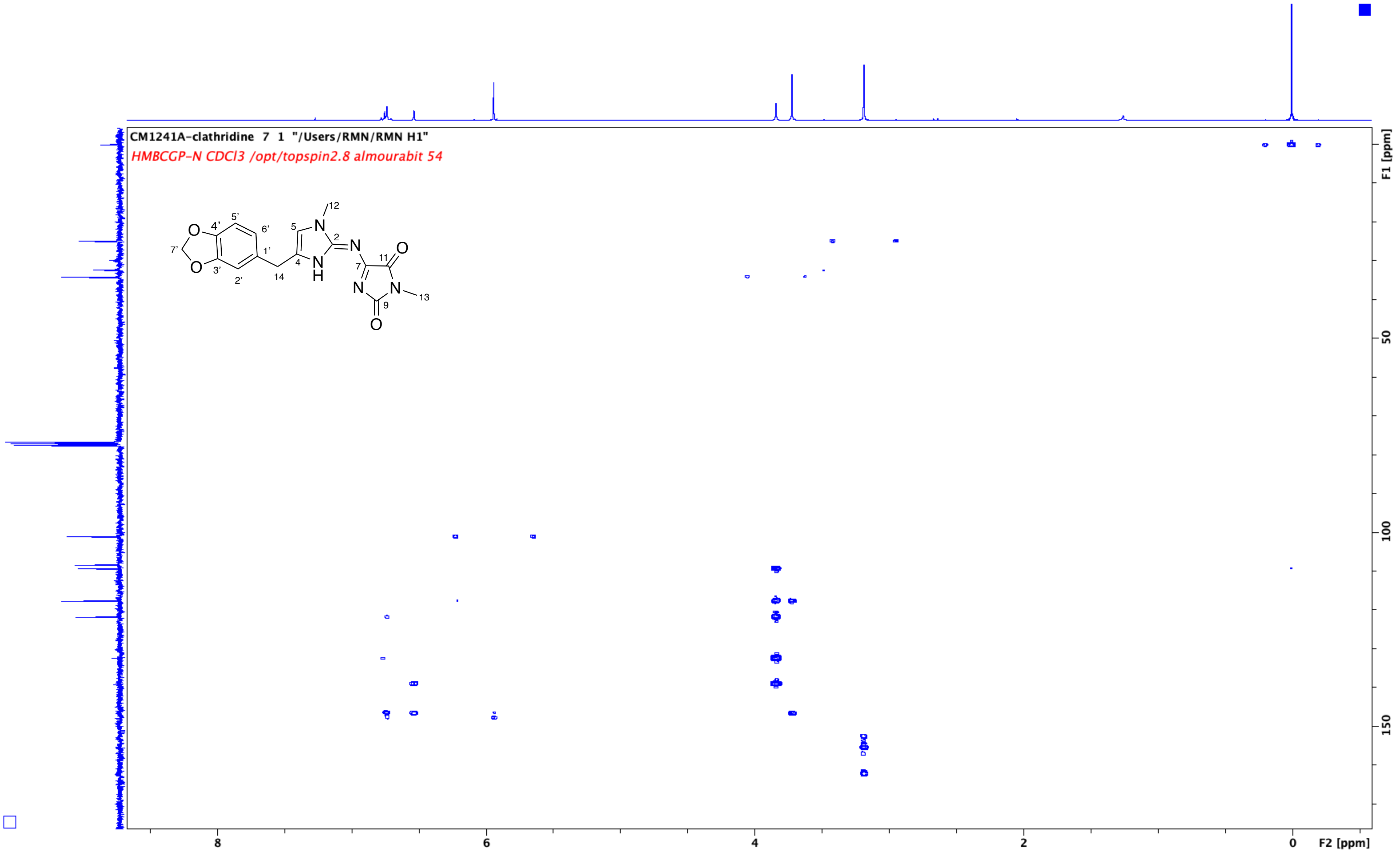

Figure S7. ^{1}H NMR spectrum of compound **18** in CDCl$_3$ (300 MHz).

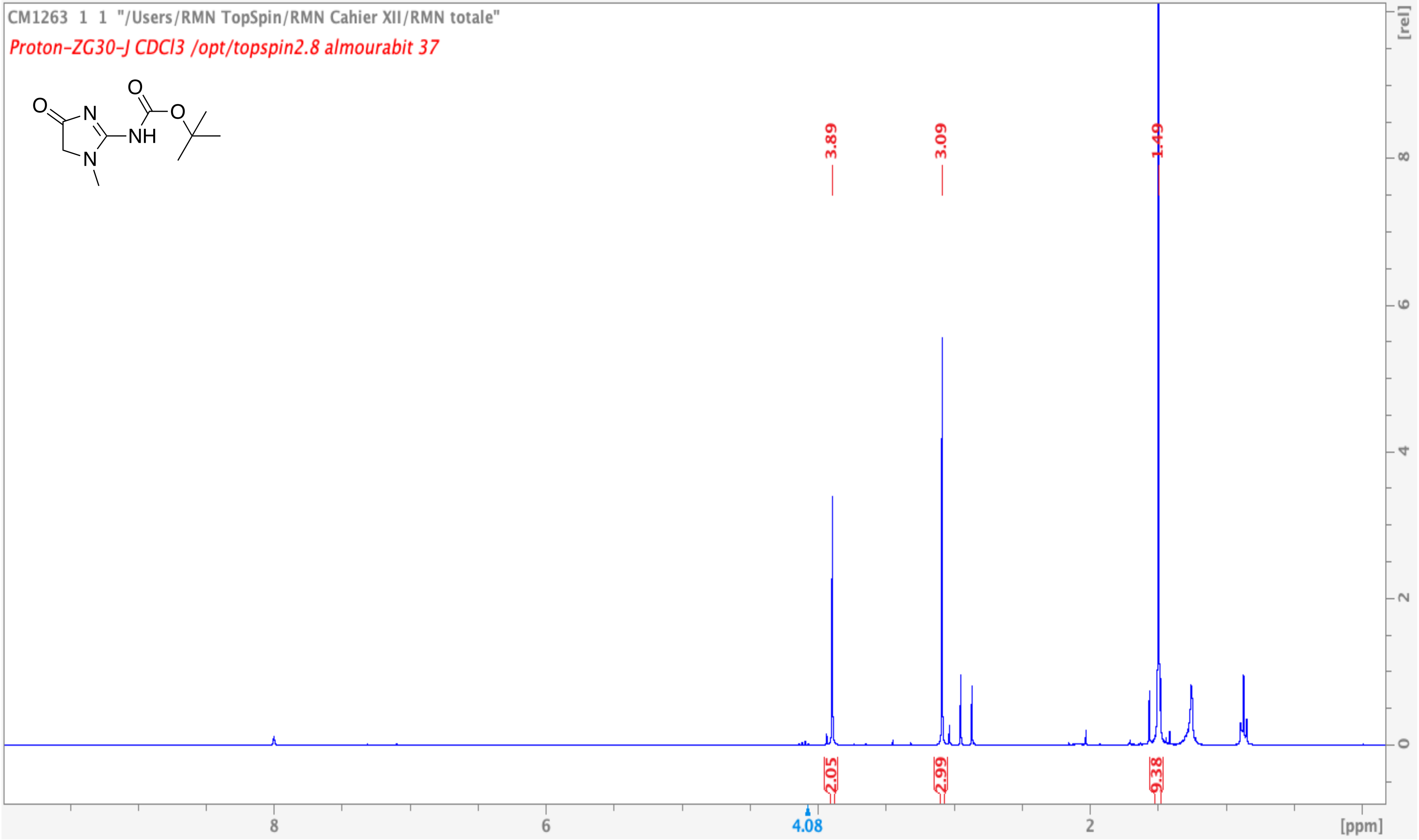

Figure S8. ^{13}C NMR spectrum of compound **18** in $CDCl_3$ (75 MHz).

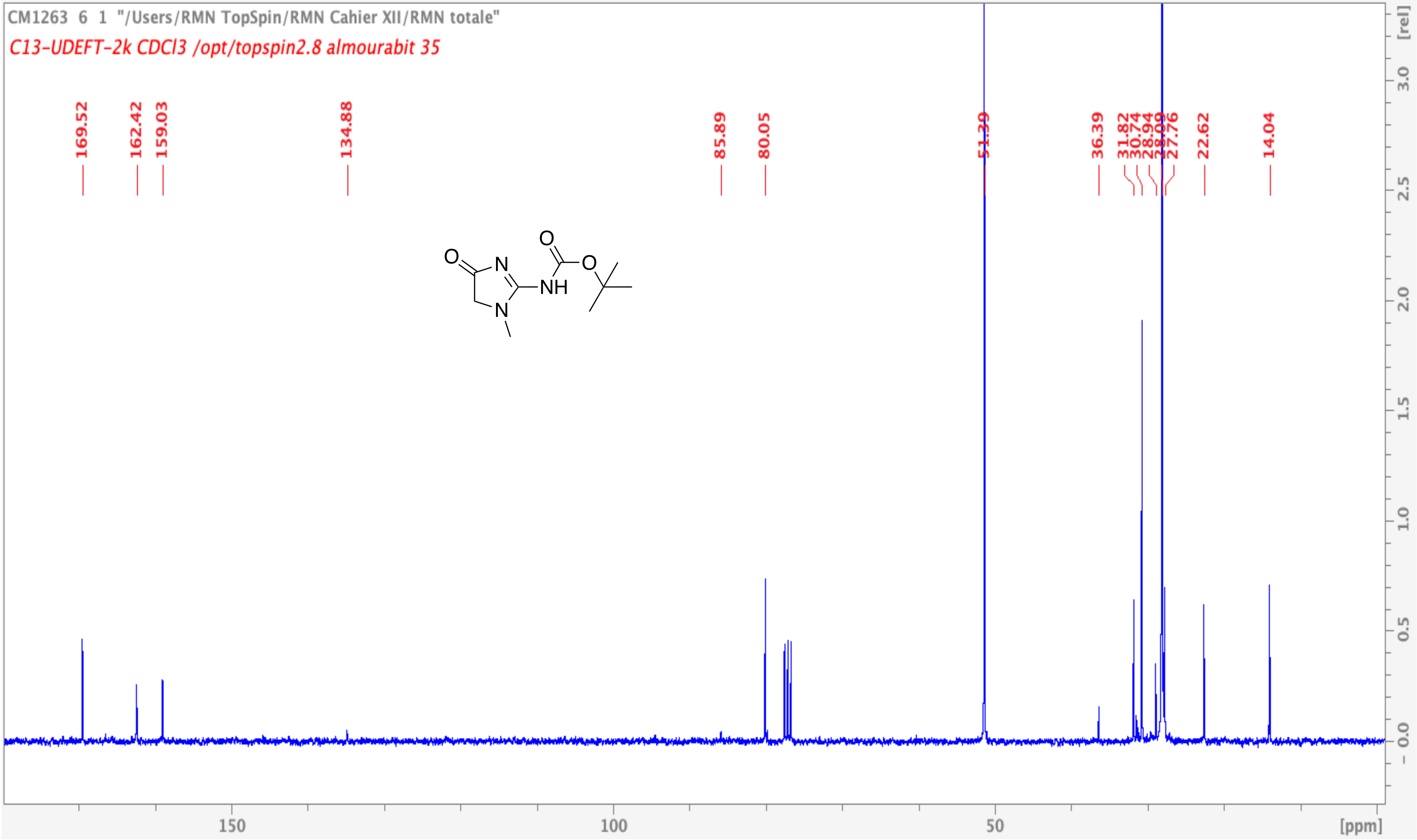

Figure S9. ^{1}H NMR spectrum of compound **19** in $CDCl_3$ (500 MHz).

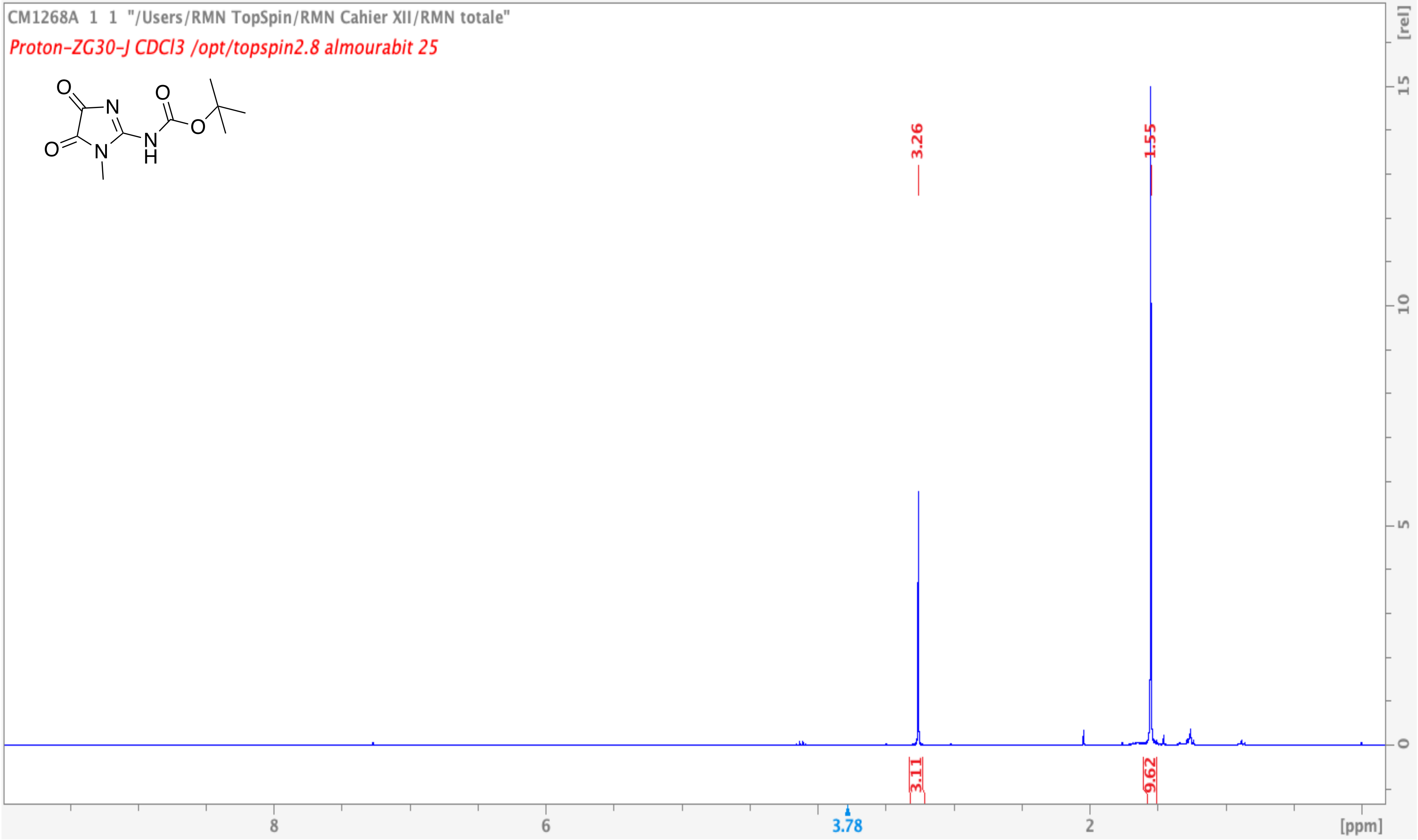

Figure S10. ^{13}C NMR spectrum of compound **19** in $CDCl_3$ (75 MHz).

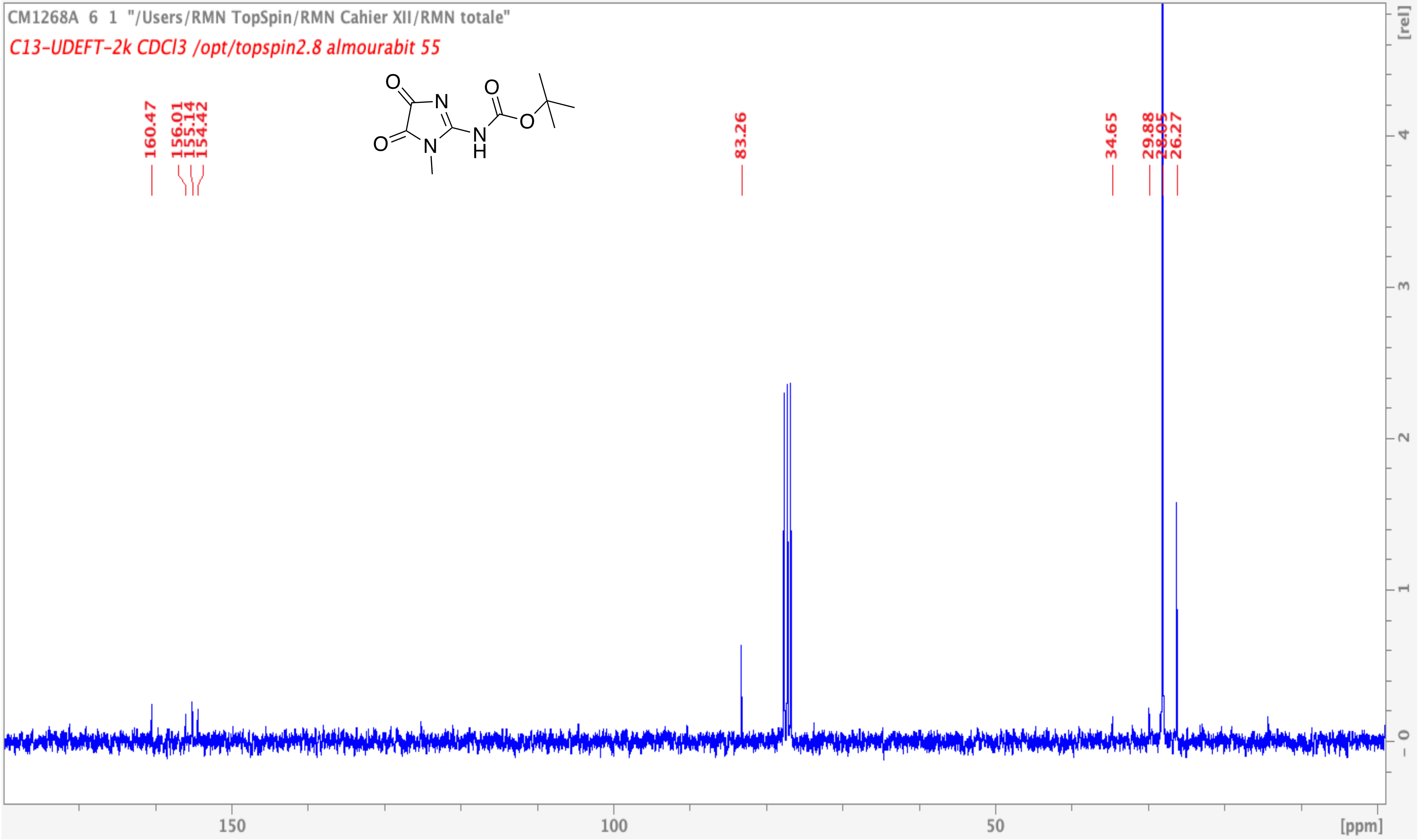

Figure S11. ^{1}H NMR spectrum of compound **20** in $CDCl_3$ (500 MHz).

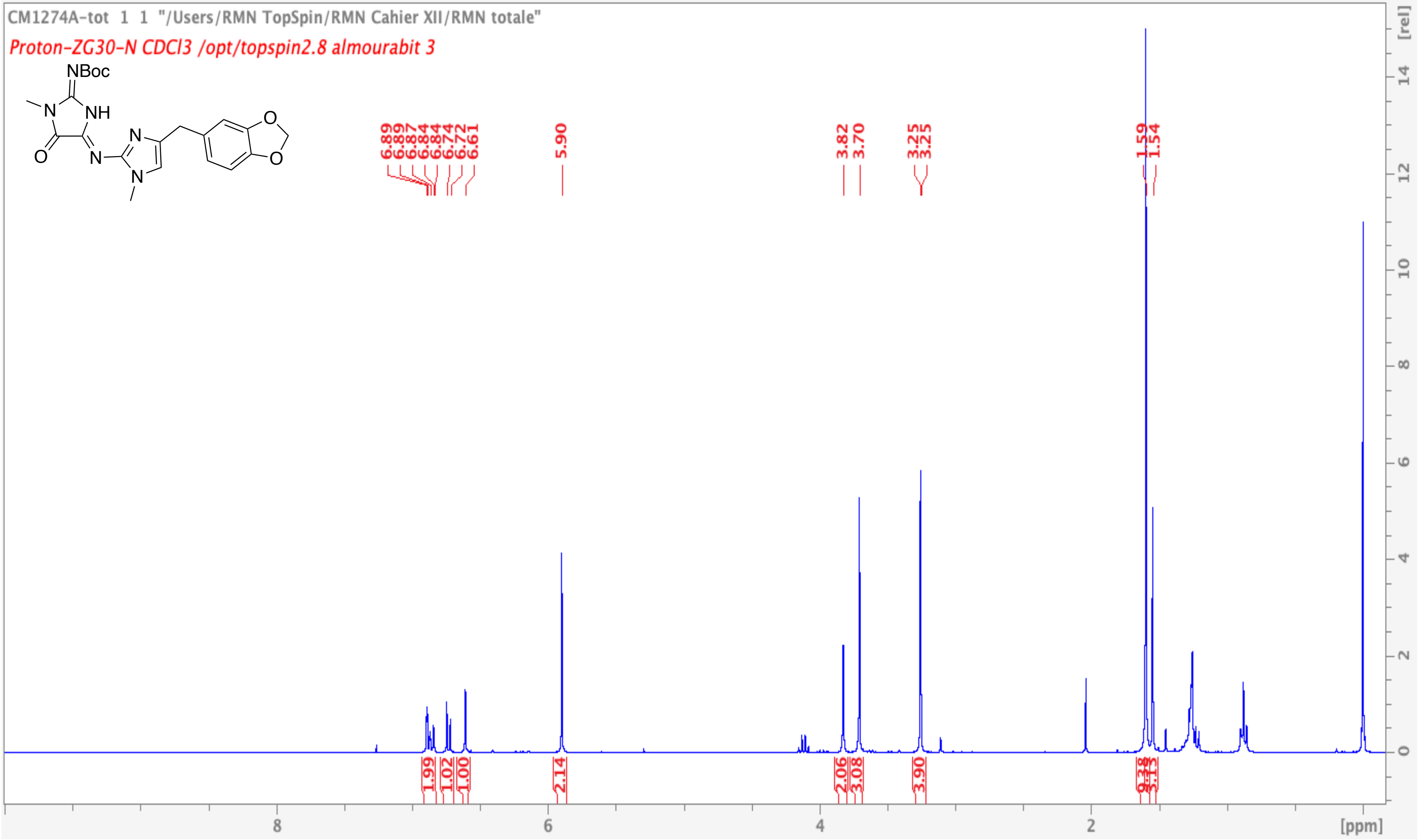

Figure S12. ^{13}C NMR spectrum of compound **20** in $CDCl_3$ (75 MHz).

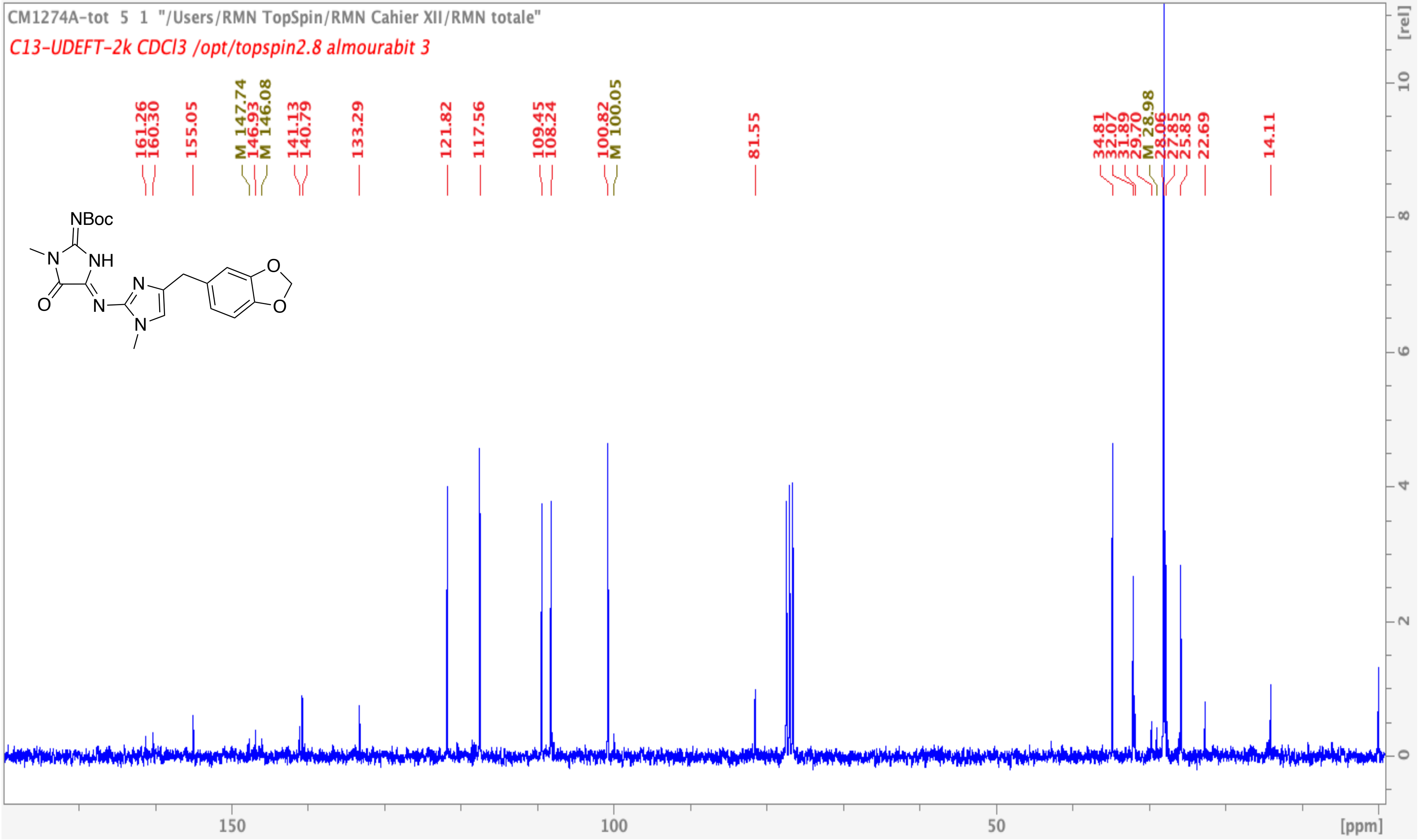

Figure S13. ^{1}H NMR spectrum of synthetic clathridimine (**4**) in $CDCl_3$ (300 MHz).

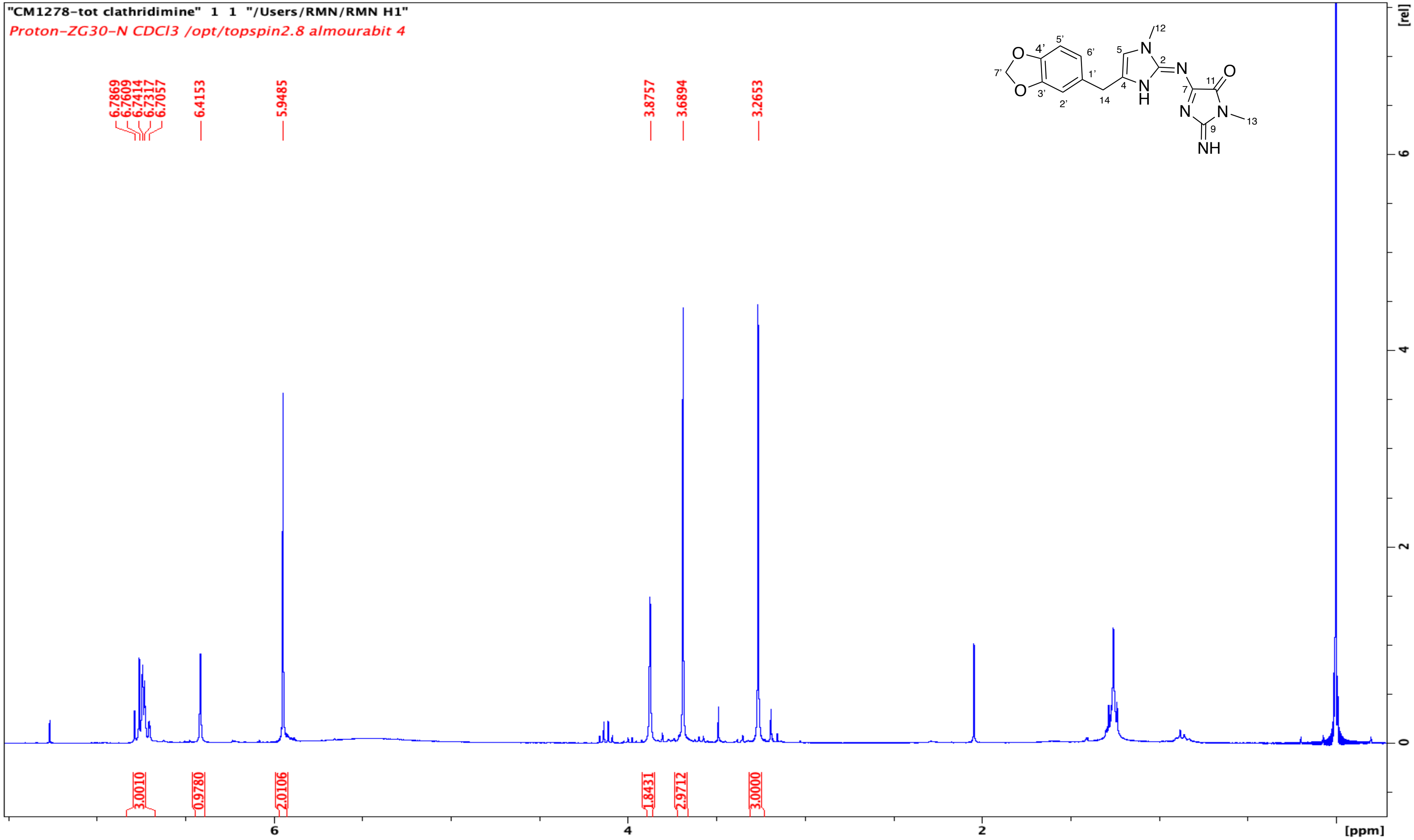

Figure S14. ^{13}C NMR spectrum of synthetic clathridimine (**4**) in $CDCl_3$ (75 MHz).

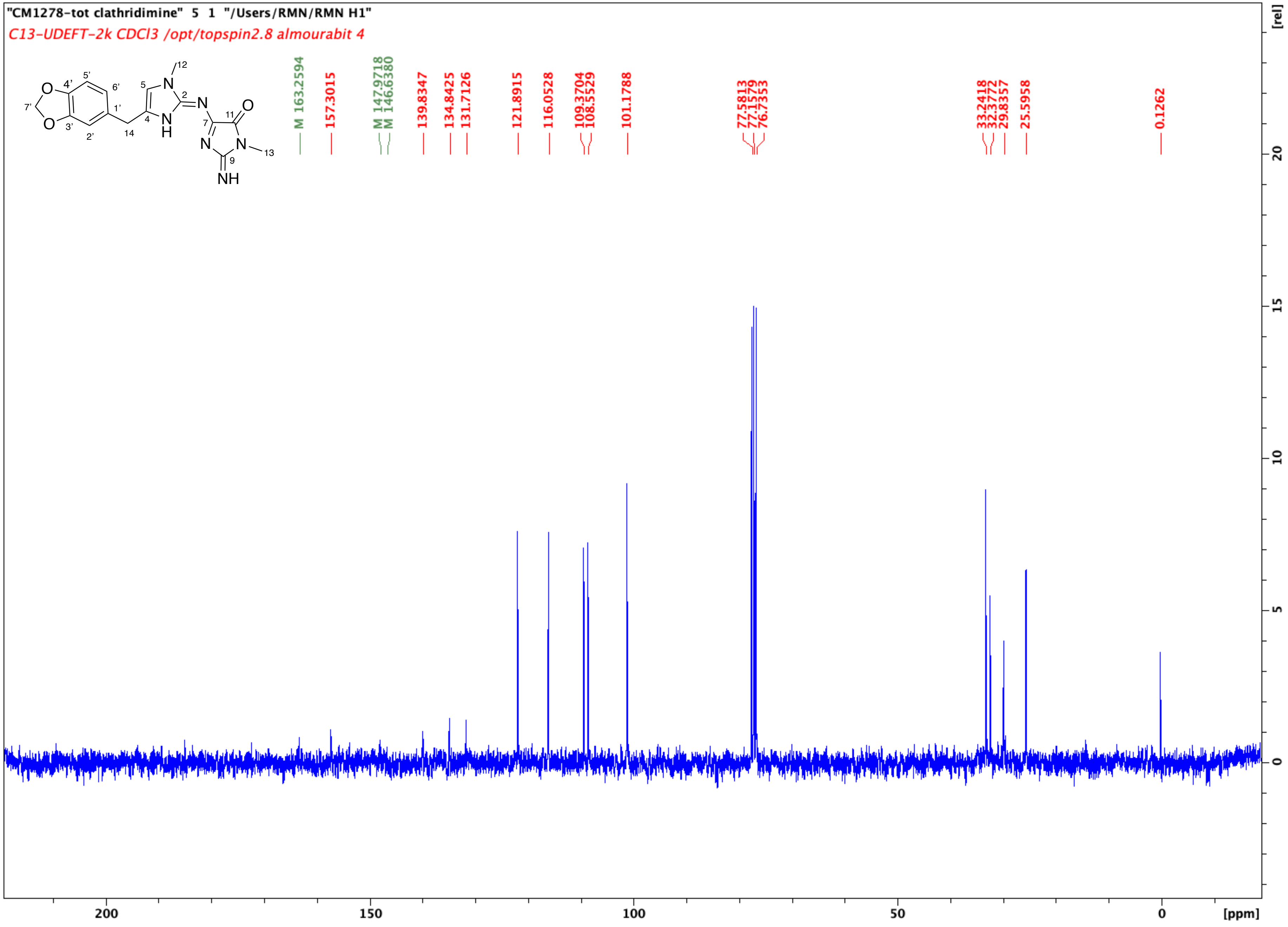

Figure S15. ^{1}H-^{13}C HMBC NMR spectrum of synthetic clathridimine (**4**) in $CDCl_3$ (300 MHz).

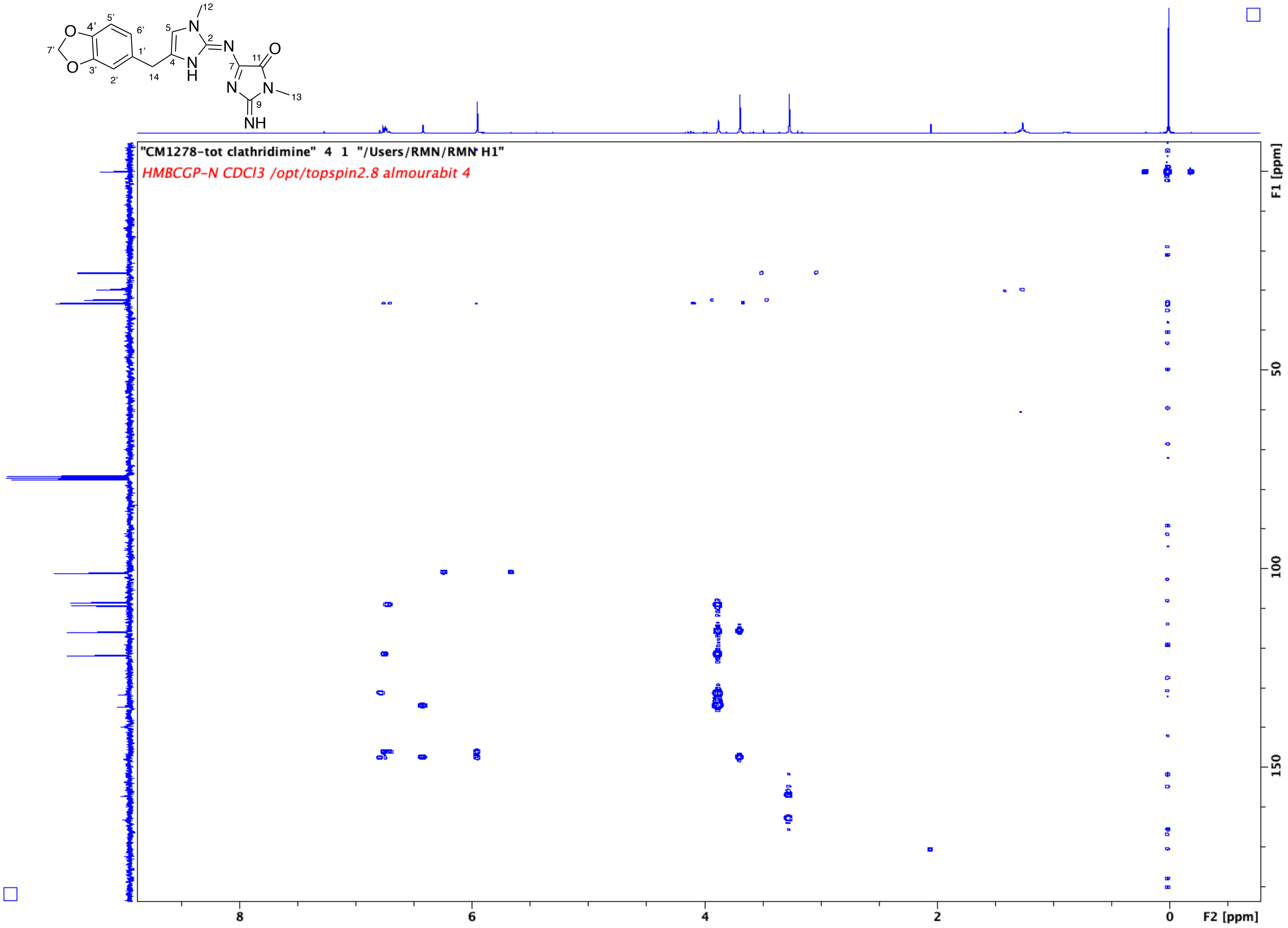

Figure S16. ^{1}H NMR spectrum of compound **20** in CD3OD (300 MHz).

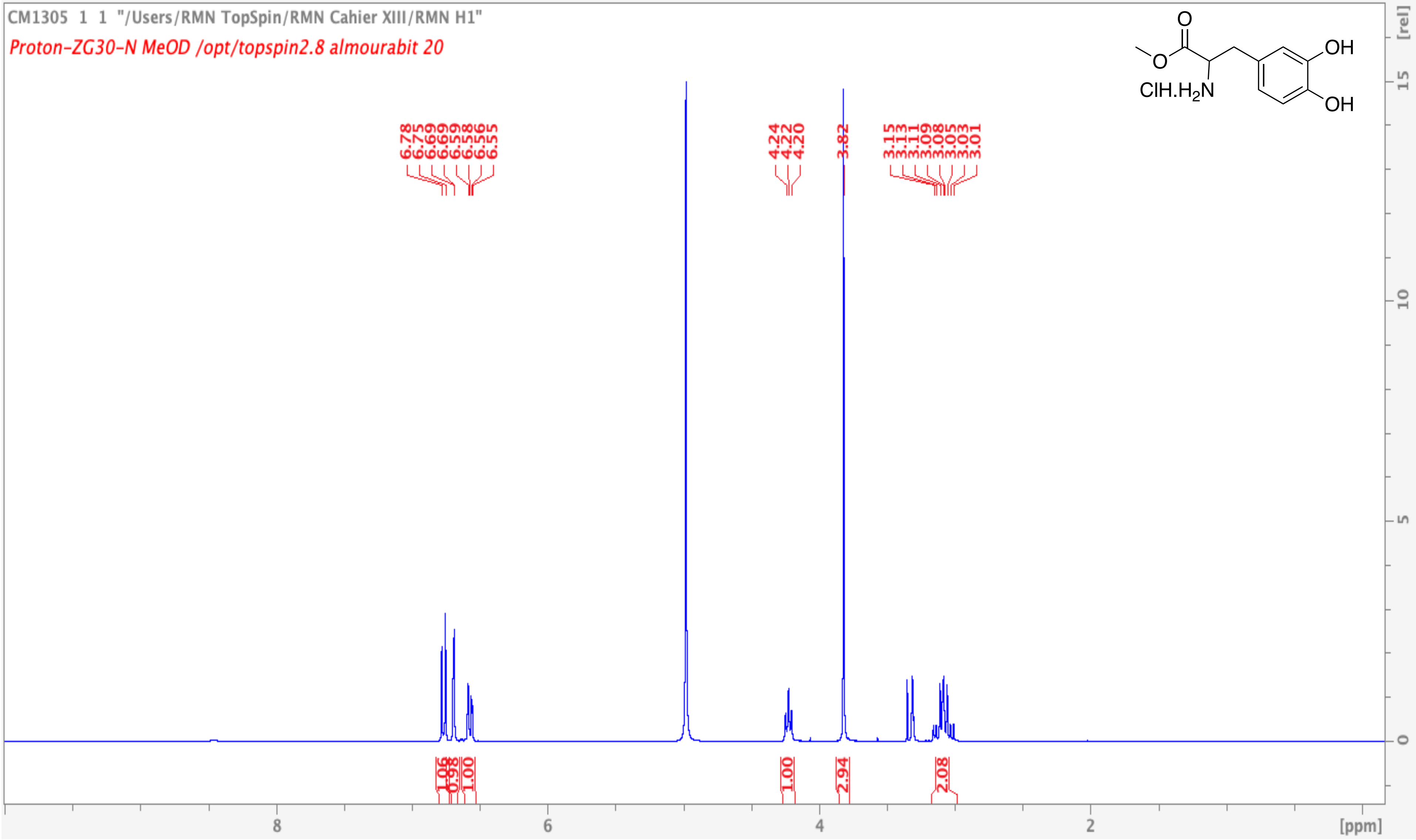

Figure S17. ^{13}C NMR spectrum of compound **20** in CD_3OD (75 MHz).

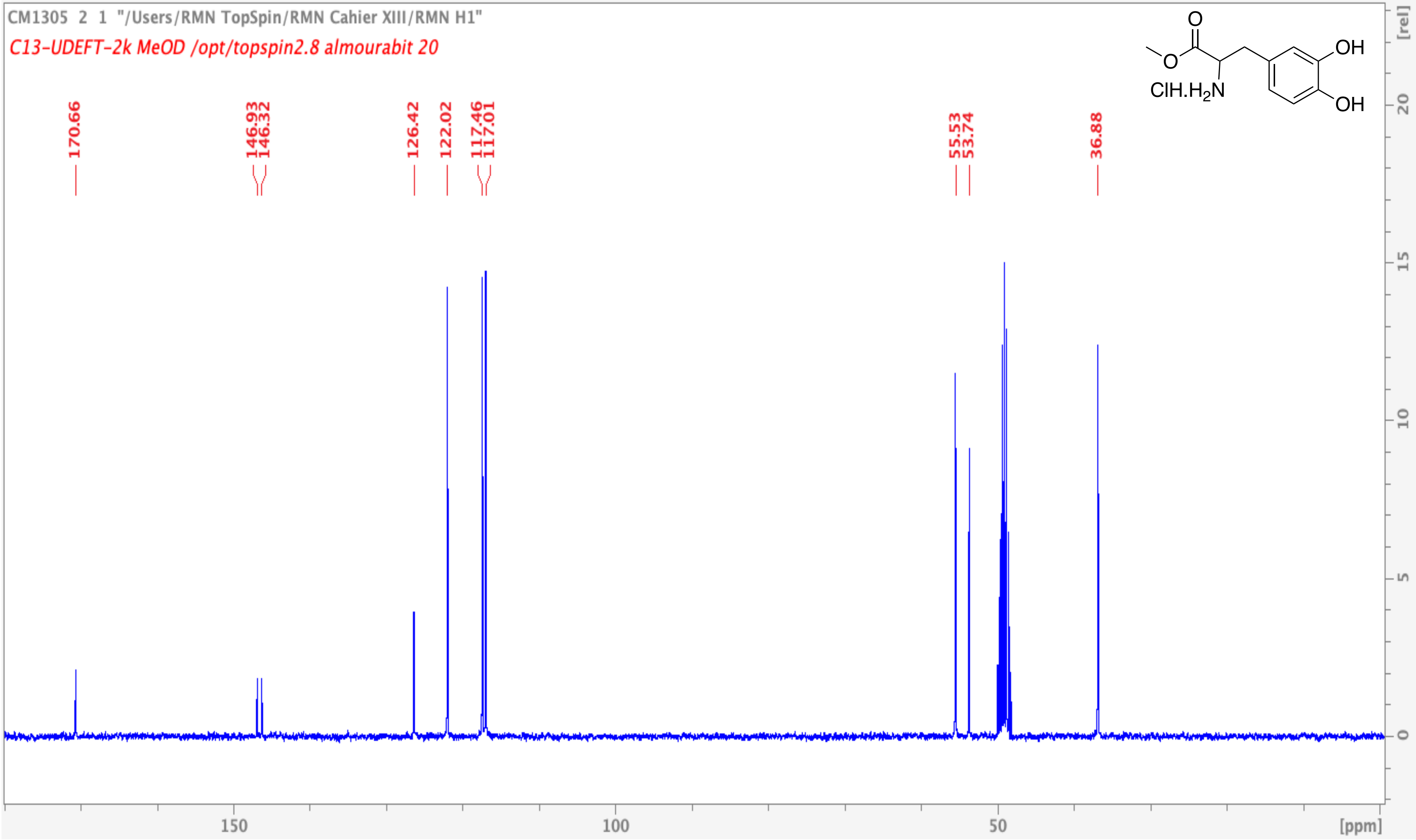

Figure S18. ^{1}H NMR spectrum of compound **21** in CD_3OD (300 MHz).

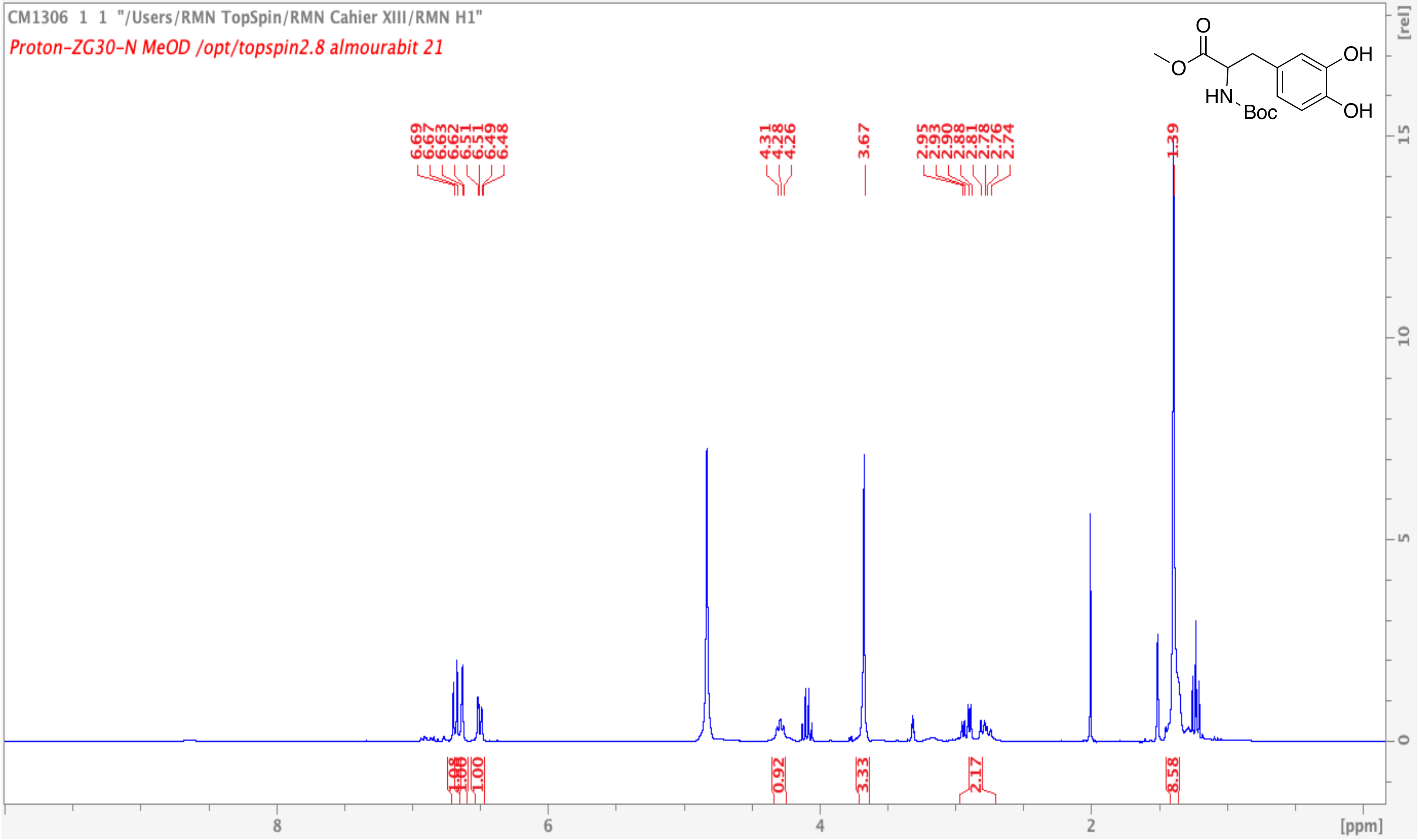

Figure S19. ^{13}C NMR spectrum of compound **21** in CD_3OD (75 MHz).

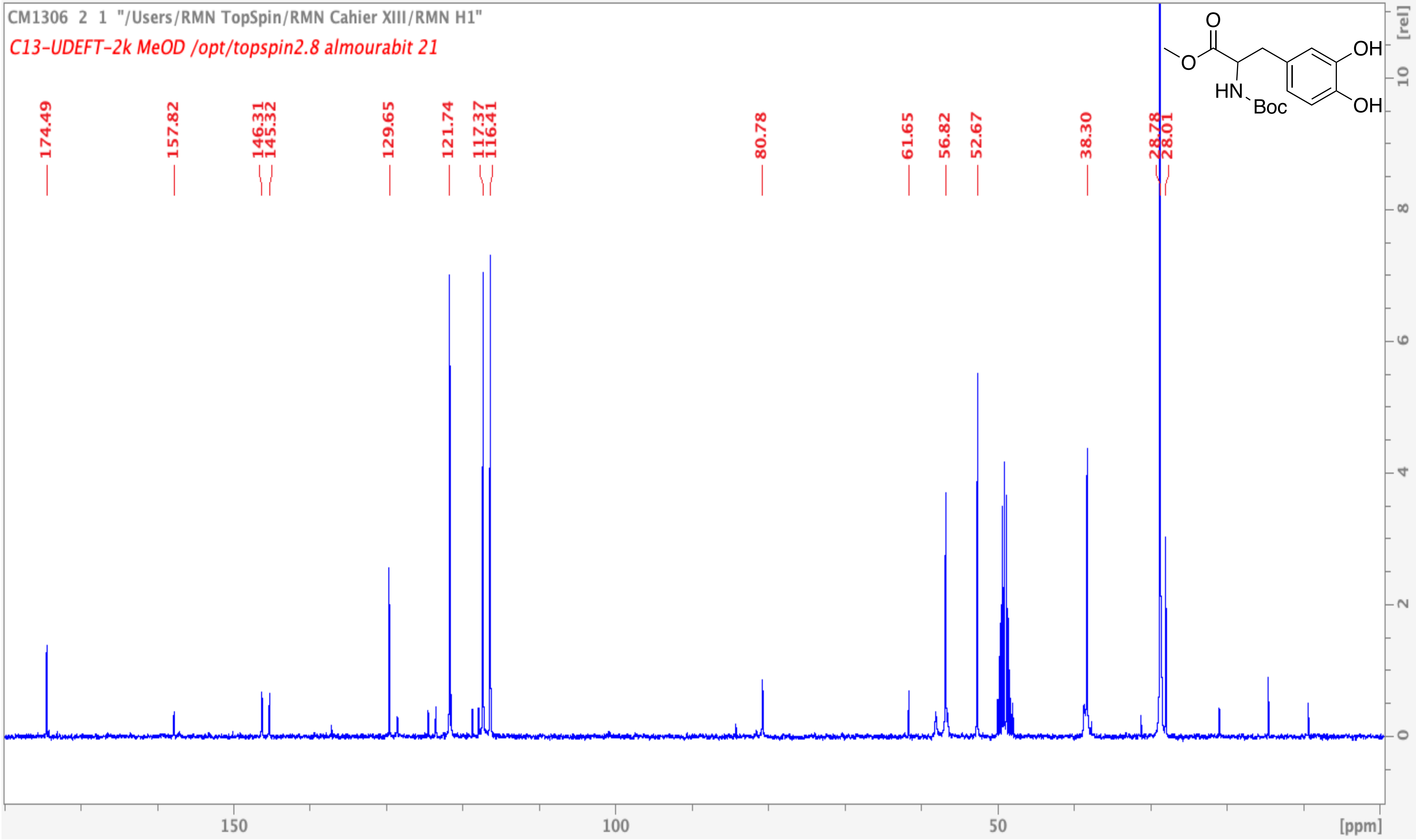

Figure S20. ^{1}H NMR spectrum of compound **22** in $CDCl_3$ (300 MHz).

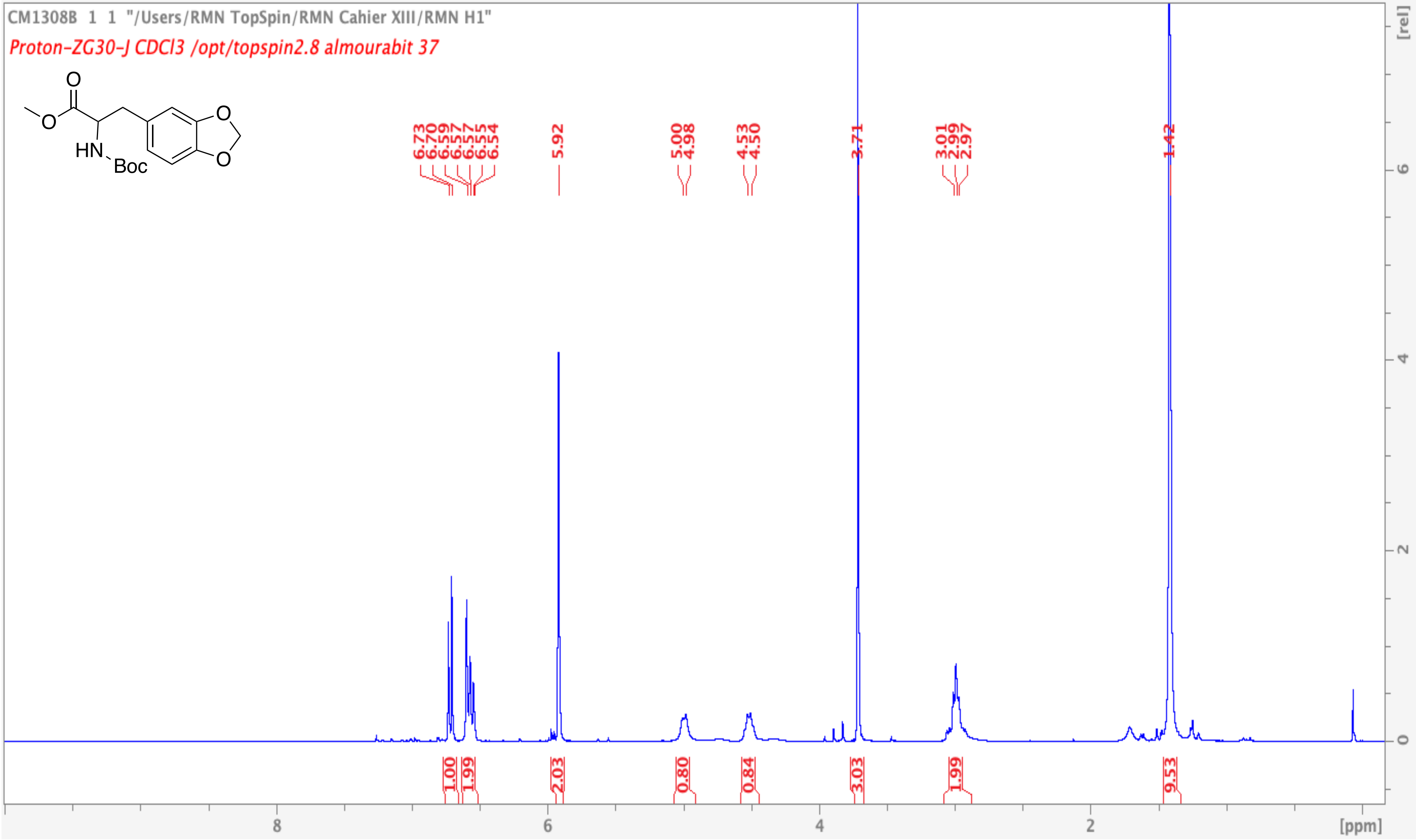

Figure S21. ^{13}C NMR spectrum of compound **22** in $CDCl_3$ (75 MHz).

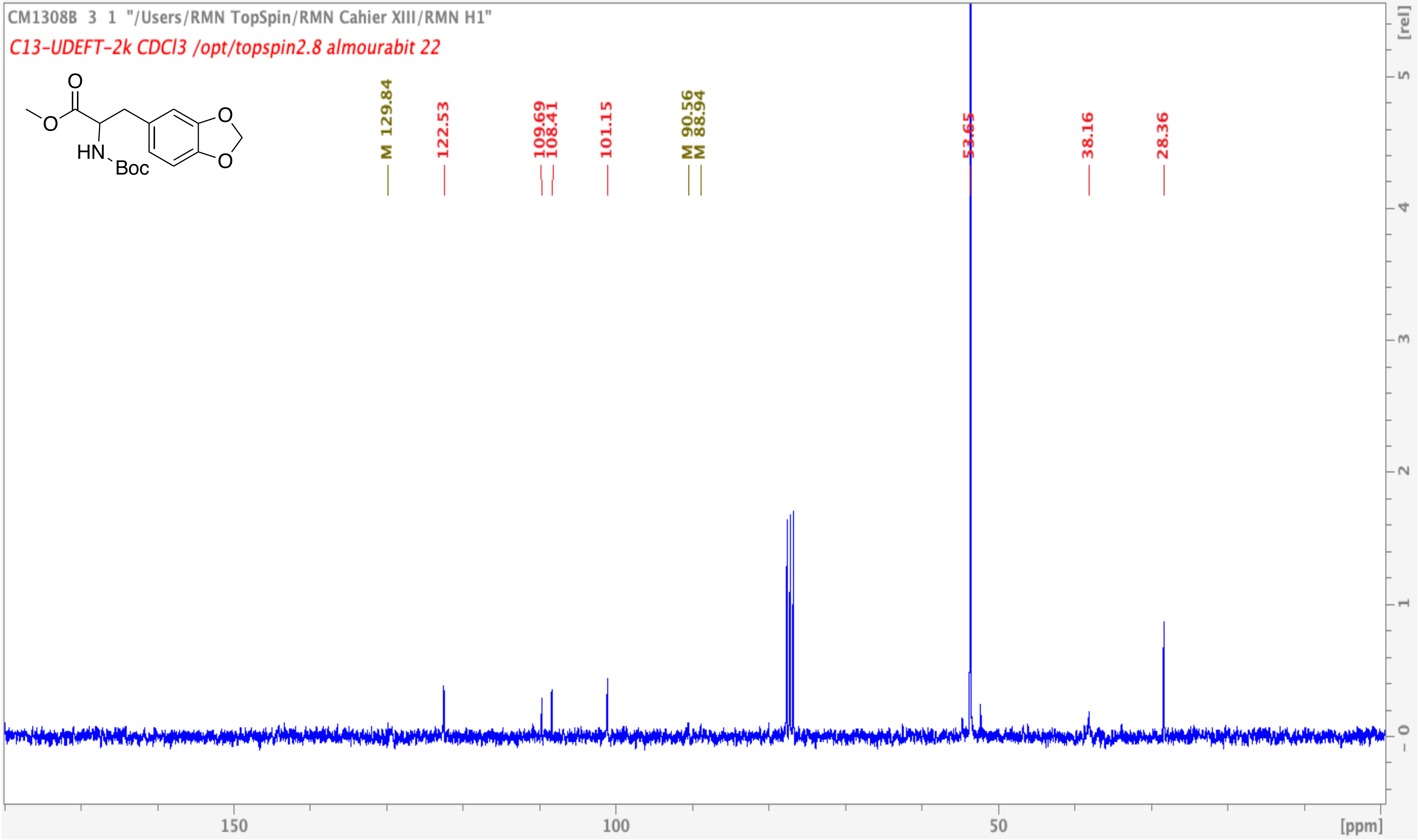

Figure S22. ^{1}H NMR spectrum of compound **23** in CD_3OD (300 MHz).

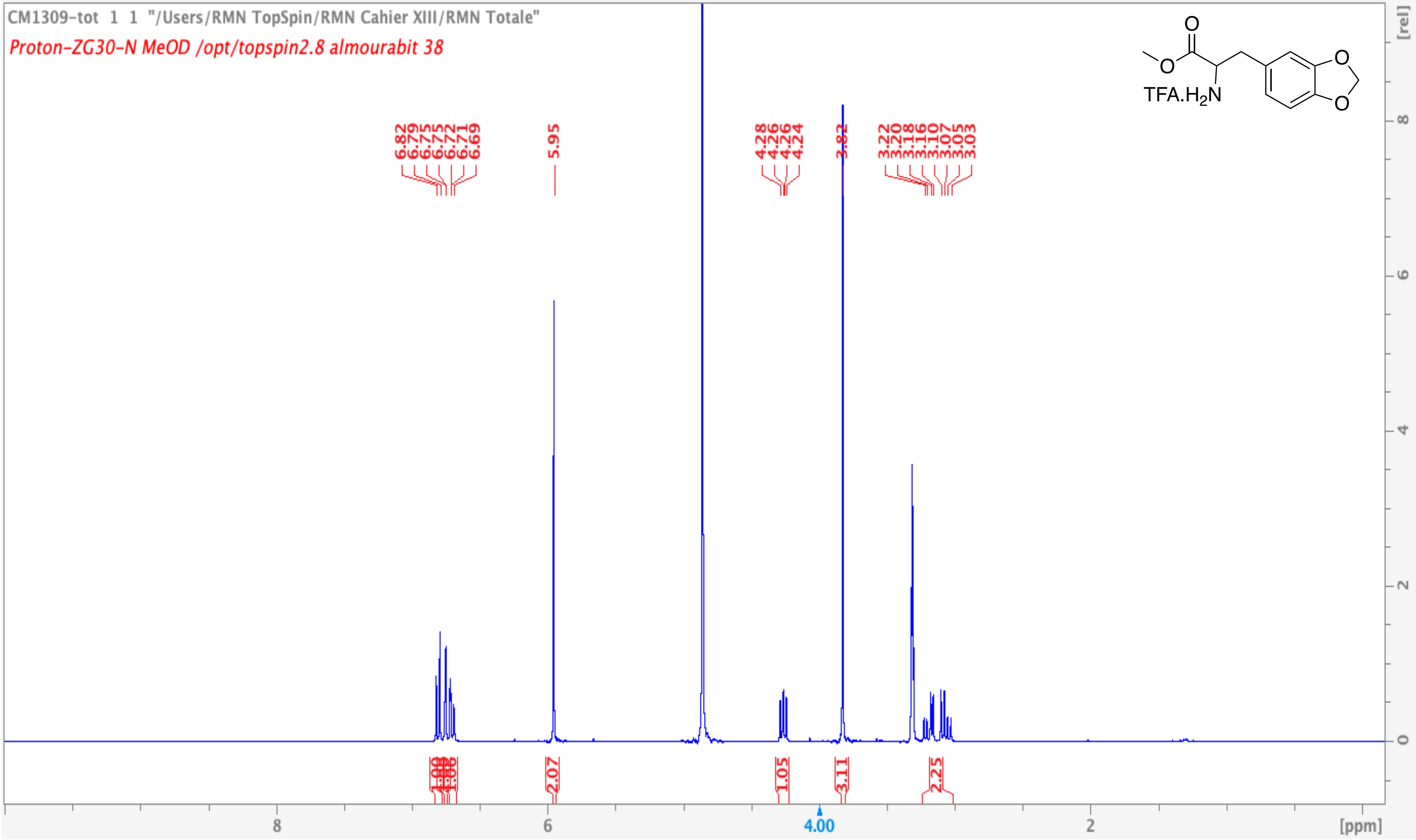

Figure S23. ^{13}C NMR spectrum of compound **23** in CD_3OD (75 MHz).

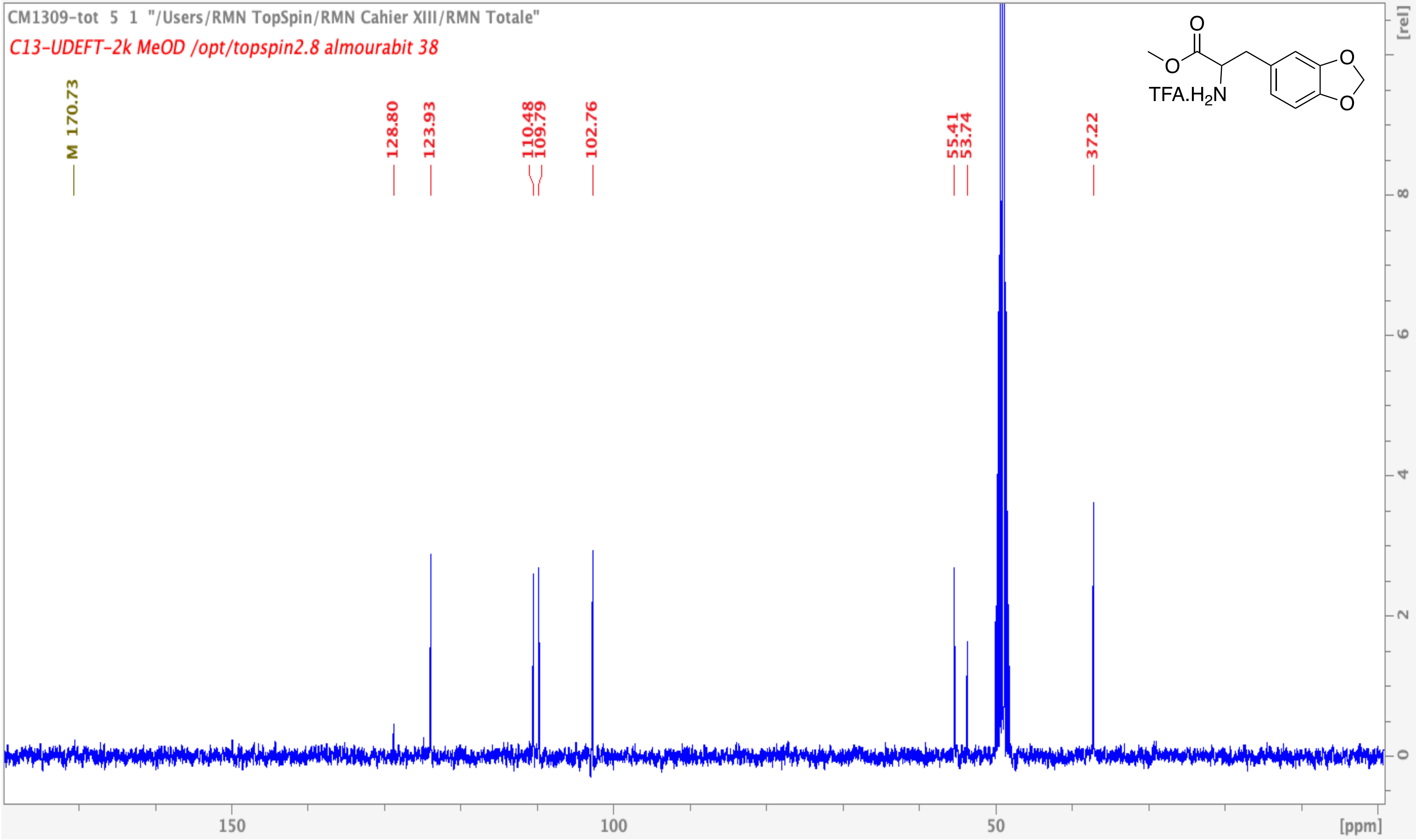

Figure S24. ^{1}H NMR spectrum of compound **24** in $CDCl_3$ (300 MHz).

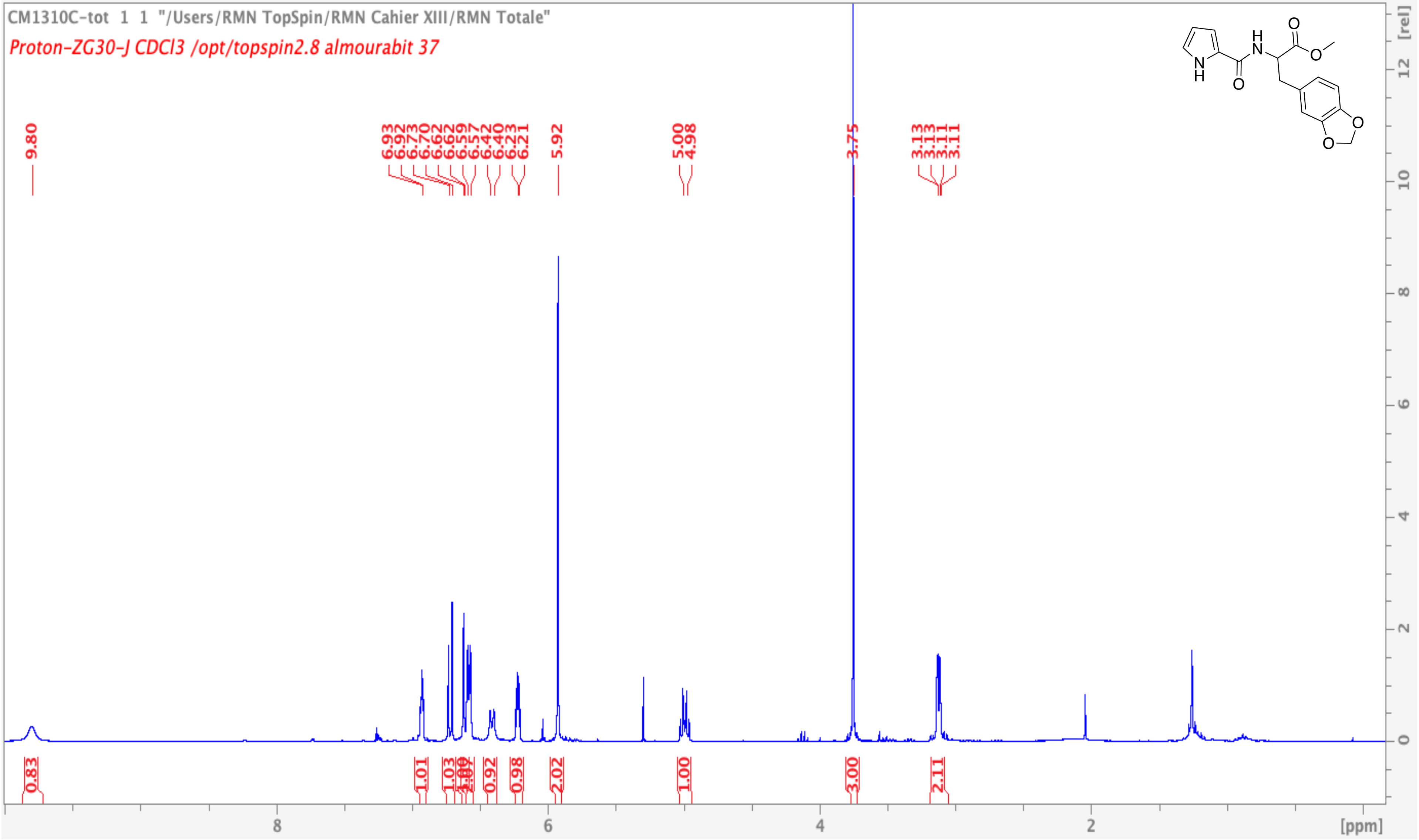

Figure S25. ^{13}C NMR spectrum of compound **24** in $CDCl_3$ (75 MHz).

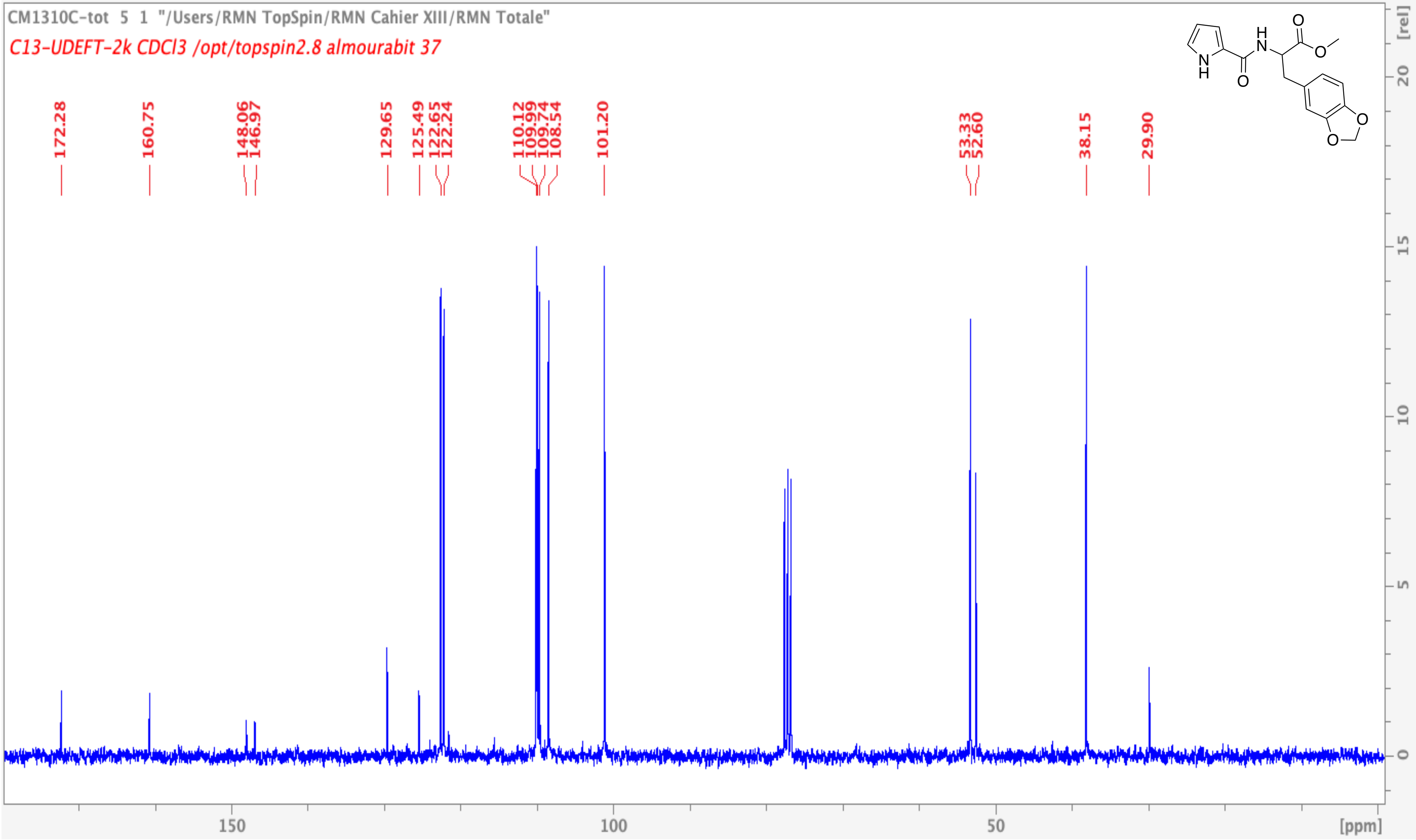

Figure S26. ^{1}H NMR spectrum of compound **25** in $CDCl_3$ (300 MHz).

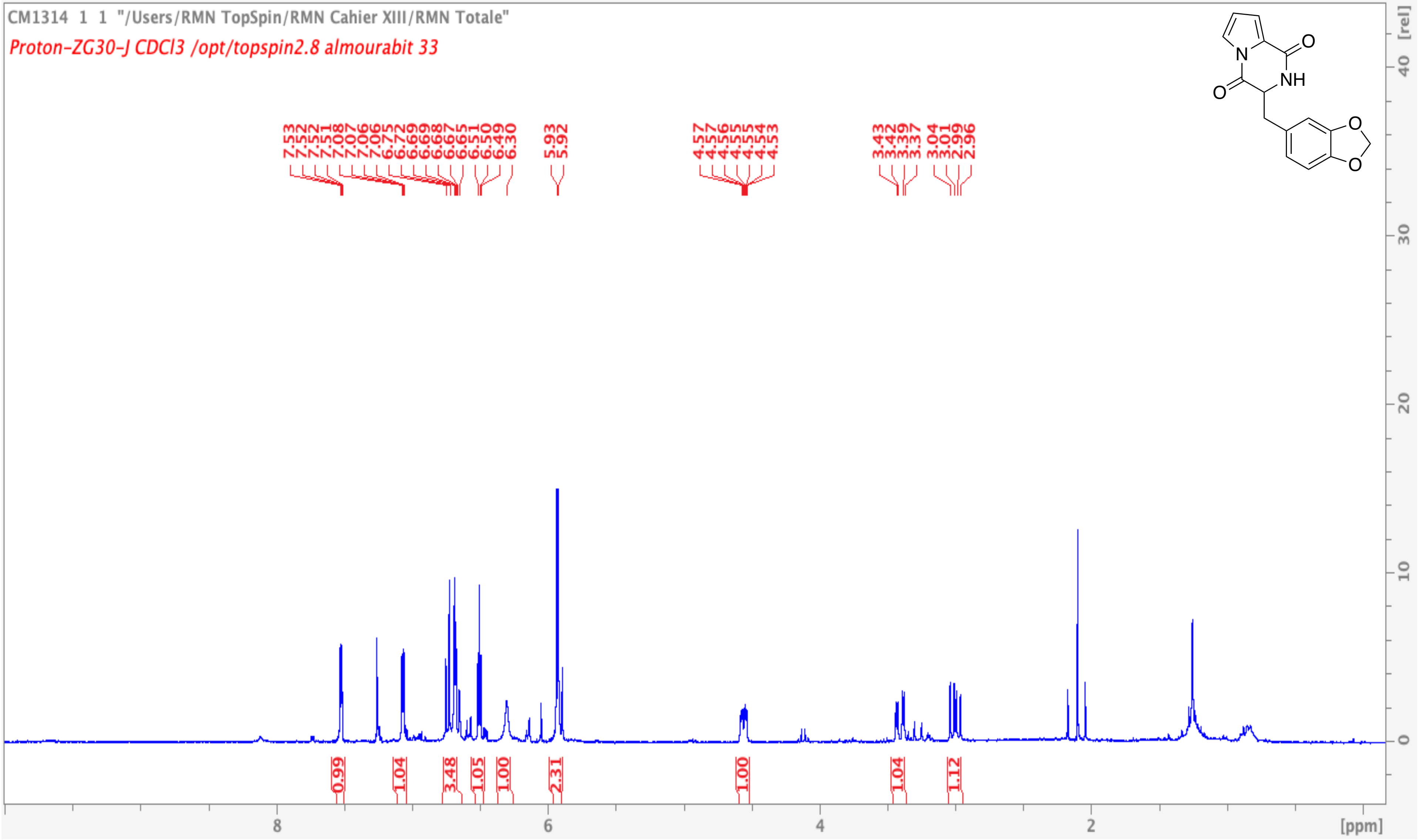

Figure S27. ^{13}C NMR spectrum of compound **25** in $CDCl_3$ (75 MHz).

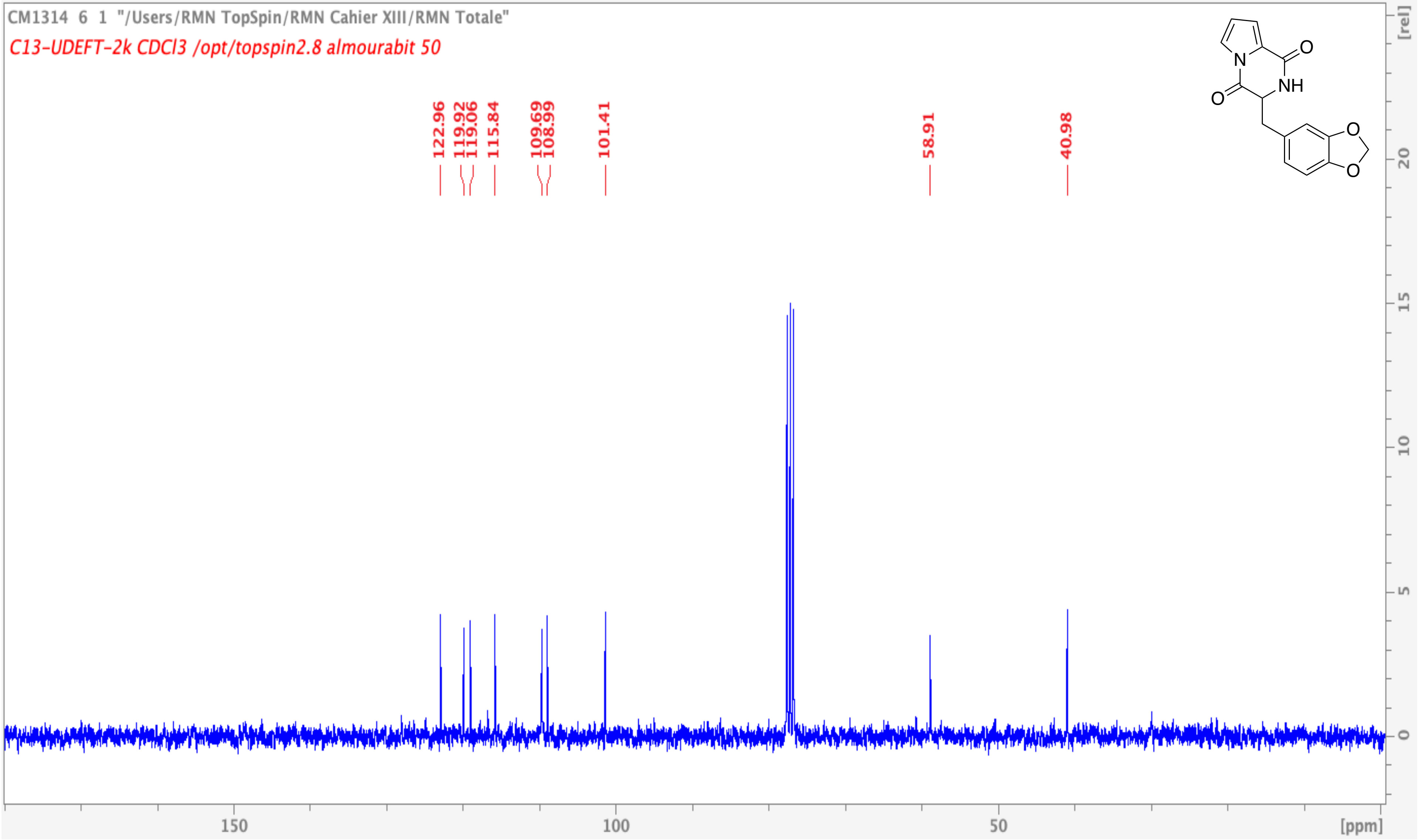

Figure S28. ^{1}H NMR spectrum of compound **26** in Acetone-d_6 (300 MHz).

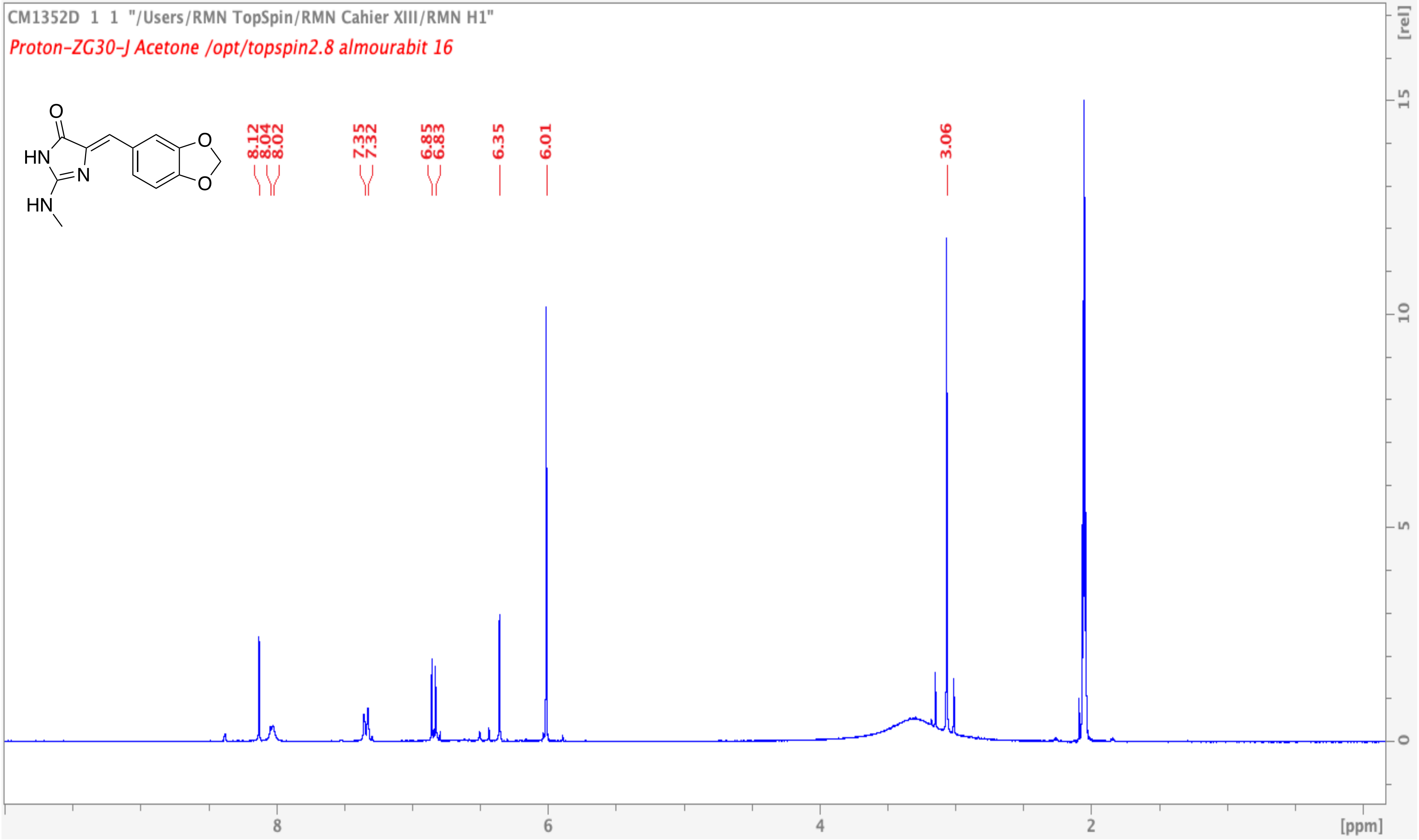

Figure S29. ^{1}H NMR spectrum of leucettamine B (**5**) in Acetone-d_6 (300 MHz).

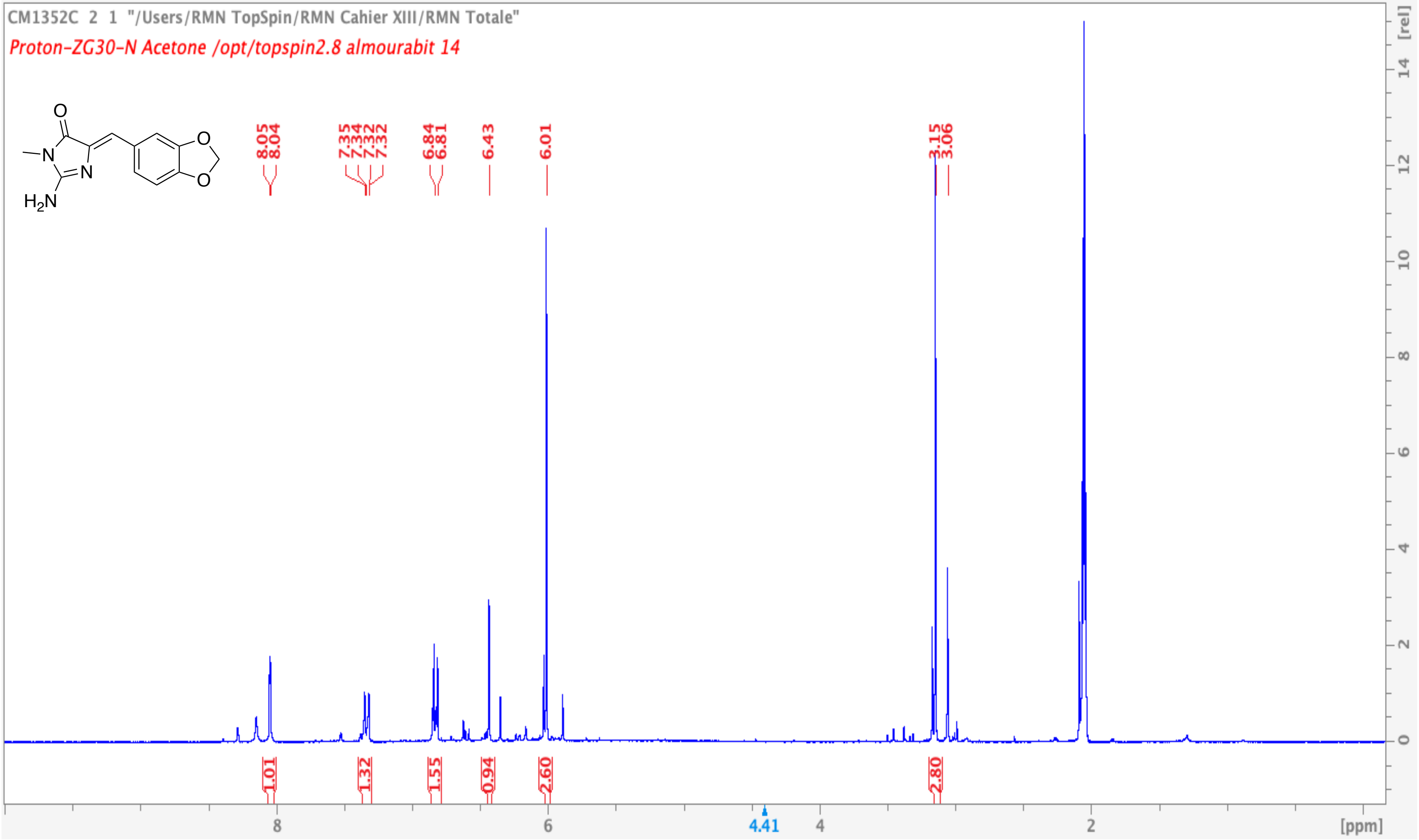

Figure S30. ^{13}C NMR spectrum of leucettamine B (**5**) in Acetone-d_6 (75 MHz).

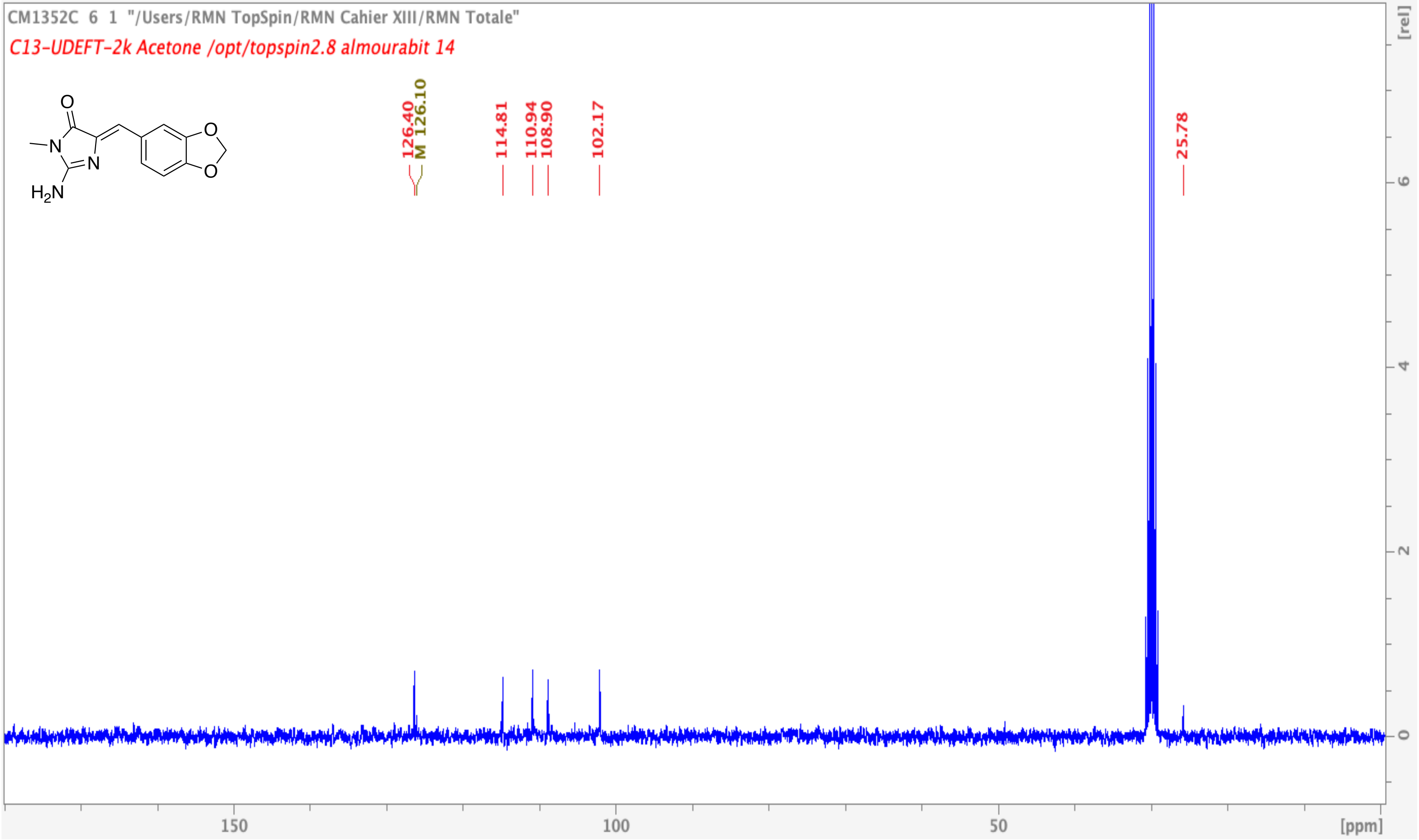

Figure S31. ^1H-^{13}C HMBC NMR spectrum of leucettamine B (**5**) in Acetone-d_6 (75 MHz).

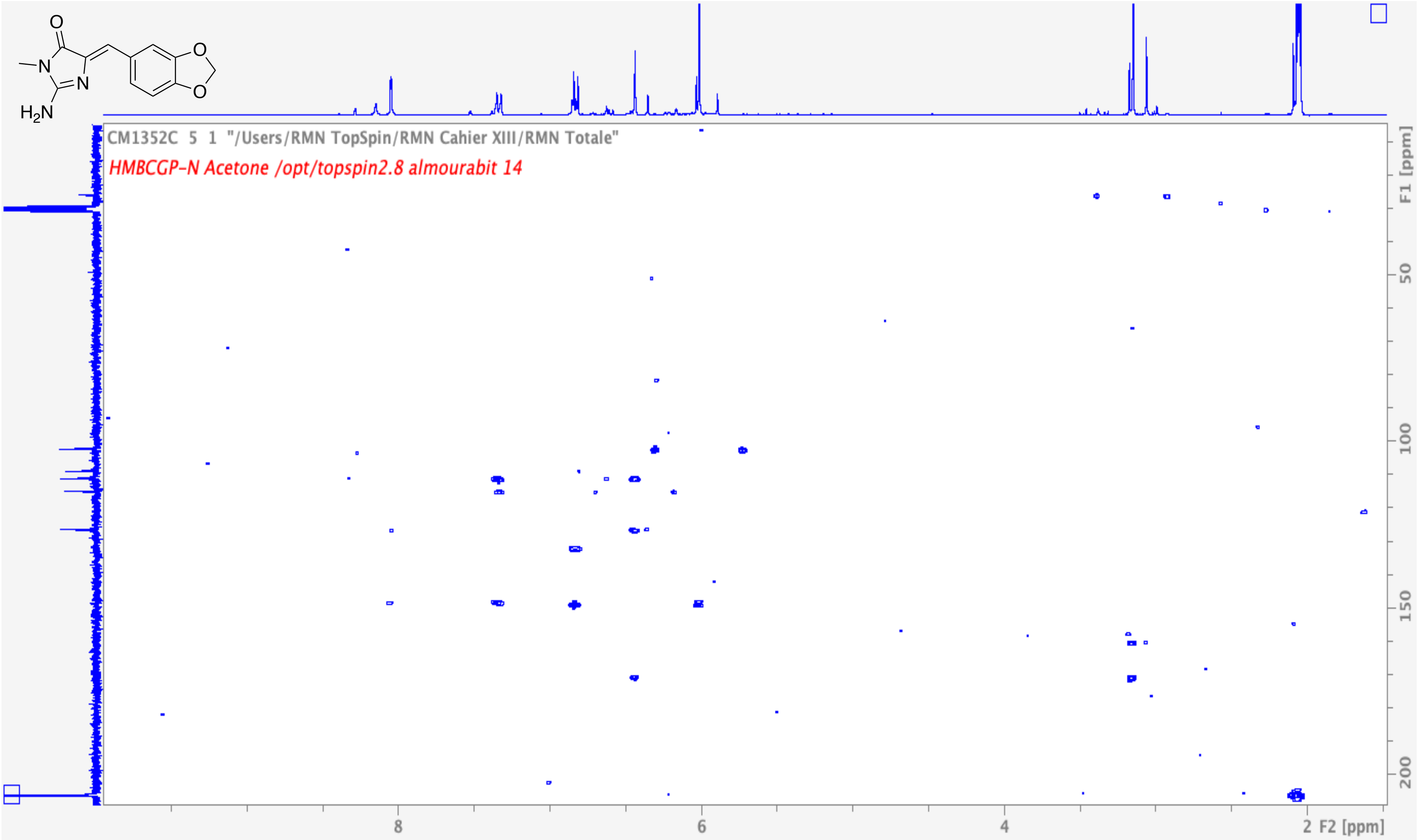

Figure S32. ^{1}H NMR spectrum of natural homodimeric (clathridine A)$_2$ Zn^{2+} (**9**) in $CDCl_3$ (500 MHz).

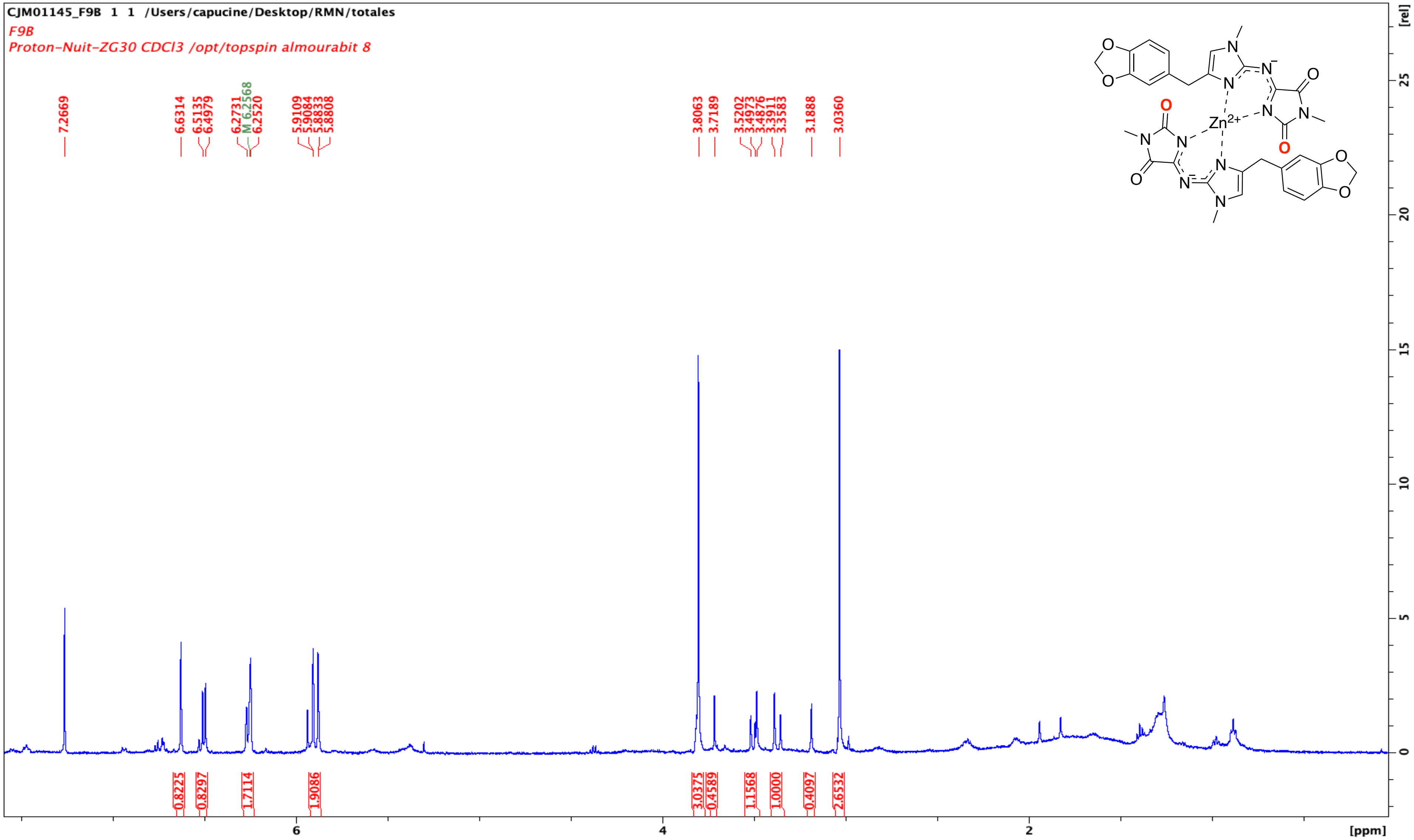

Figure S33. ^{1}H-^{13}C HSQC NMR spectrum of natural homodimeric (clathridine A)$_2$ Zn^{2+} (**9**) in $CDCl_3$ (500 MHz).

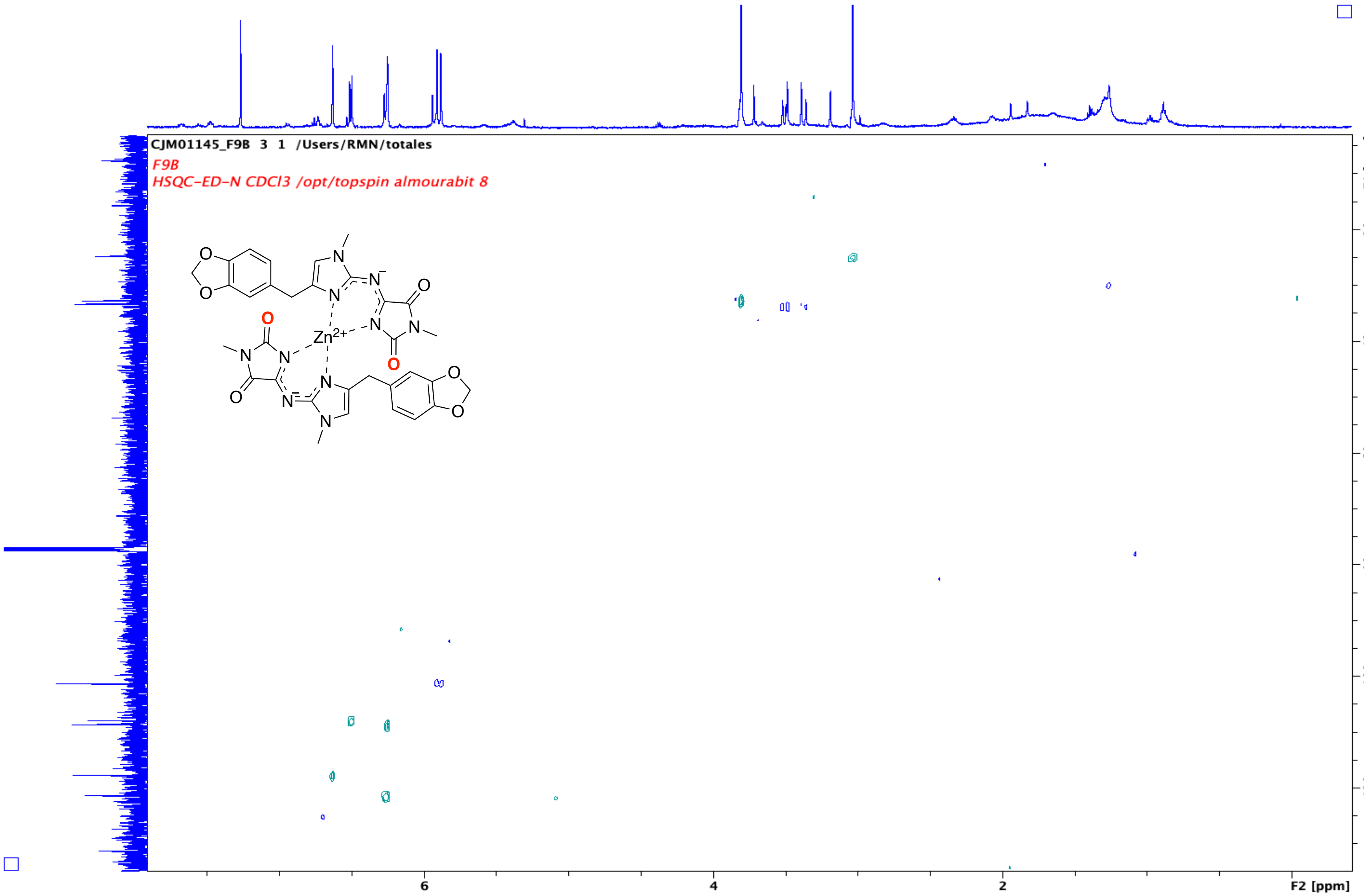

Figure S34. ^{1}H-^{13}C HMBC NMR spectrum of natural homodimeric (clathridine A)$_2$ Zn^{2+} (**9**) in $CDCl_3$ (500 MHz).

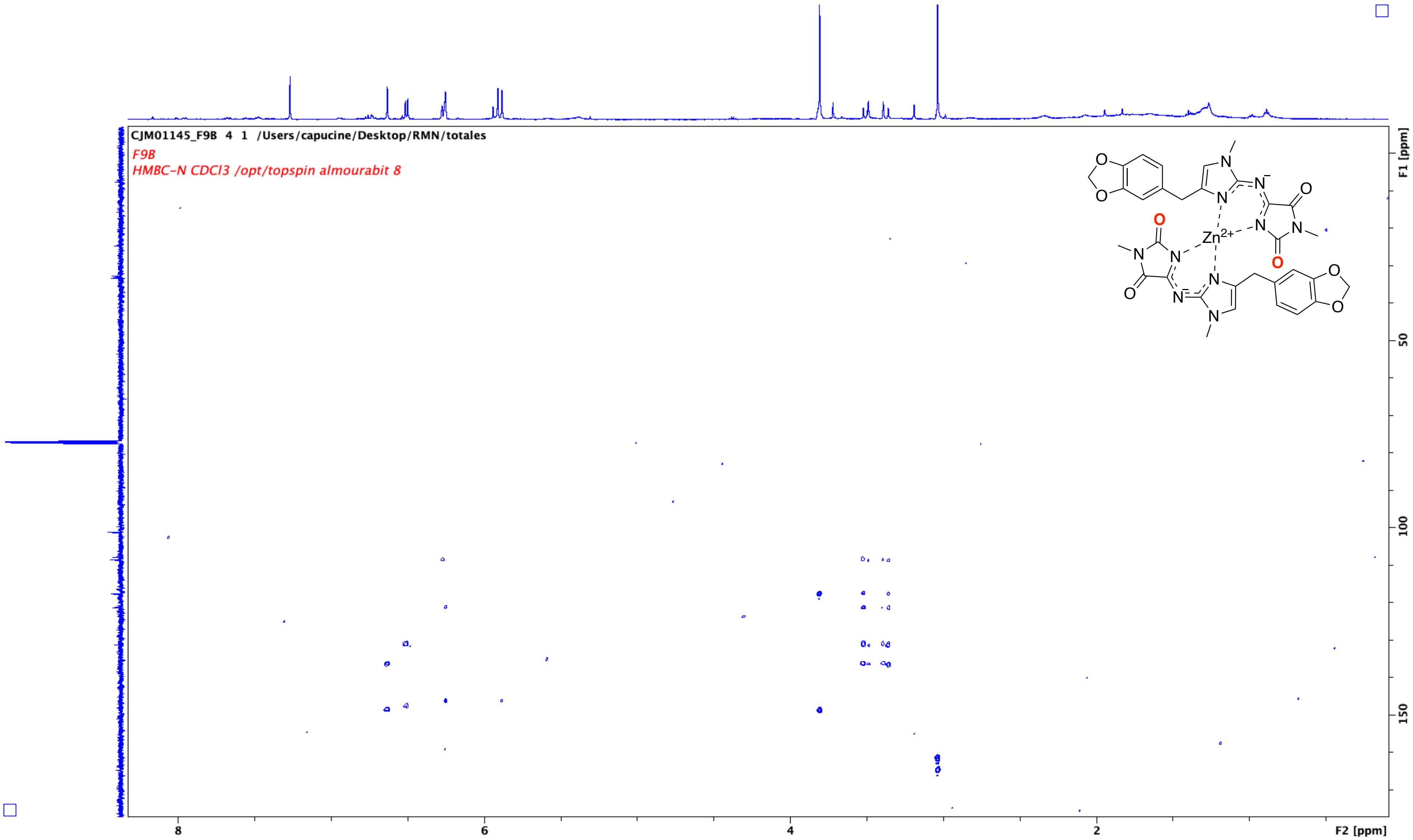

Figure S35. ^{1}H NMR spectrum of synthetic homodimeric (clathridine A)$_2$ Zn^{2+} (**9**) in $CDCl_3$ (500 MHz).

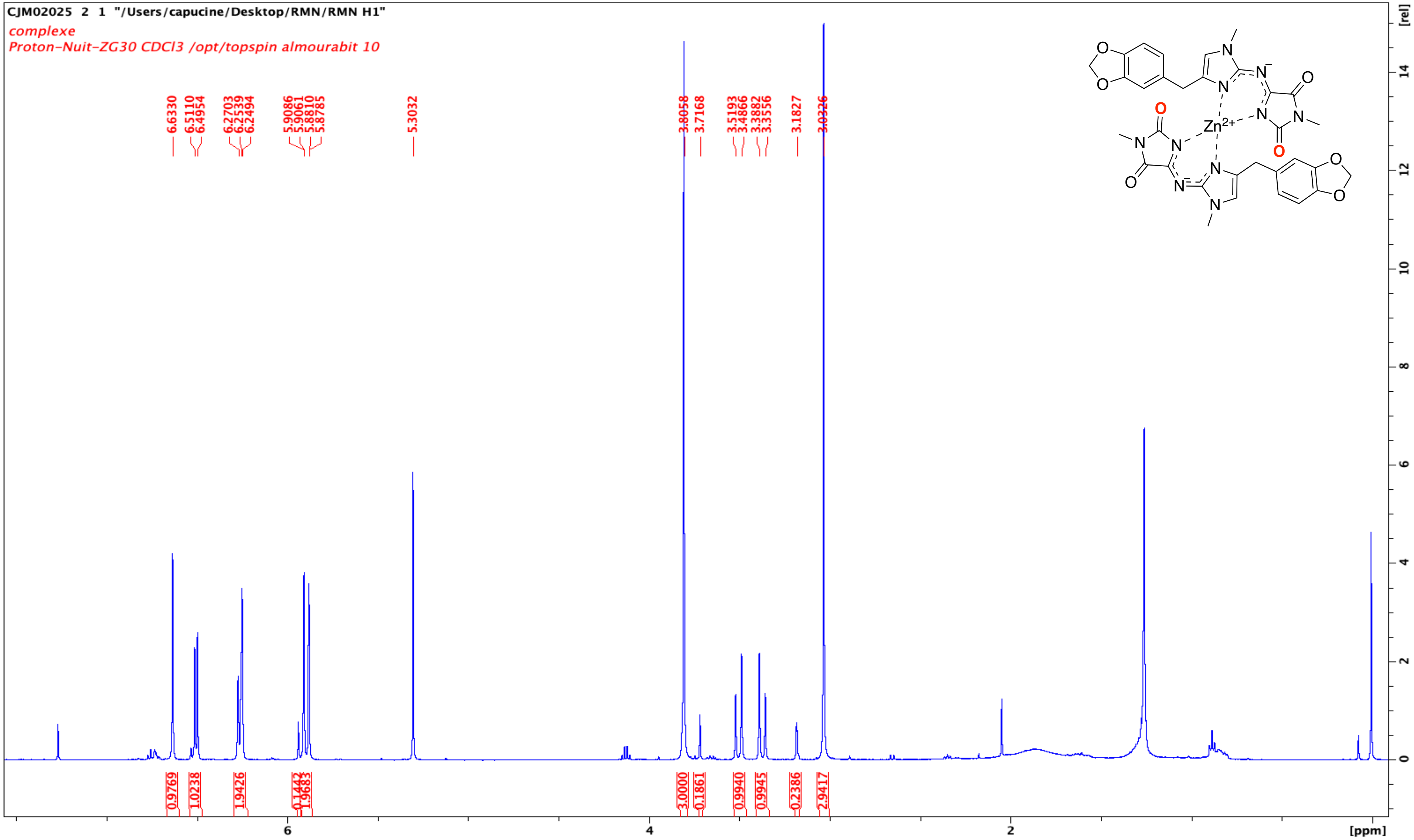

Figure S36. ^{13}C NMR spectrum of synthetic homodimeric (clathridine A)$_2$ Zn^{2+} (**9**) in $CDCl_3$ (125 MHz).

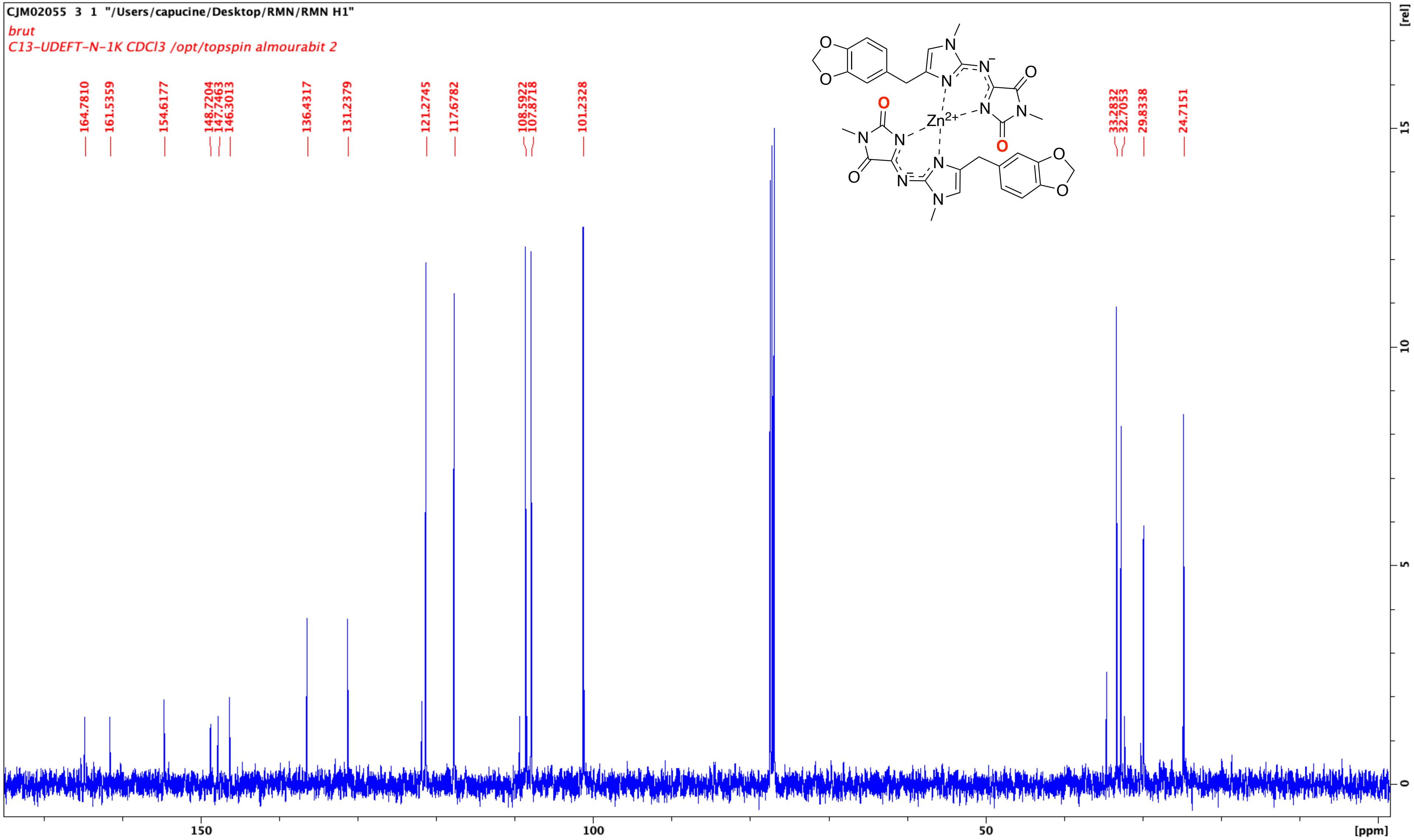

Figure S37. HR-ESI mass spectrum of the synthetic homodimeric (clathridine A)$_2$ Zn^{2+} (**9**).

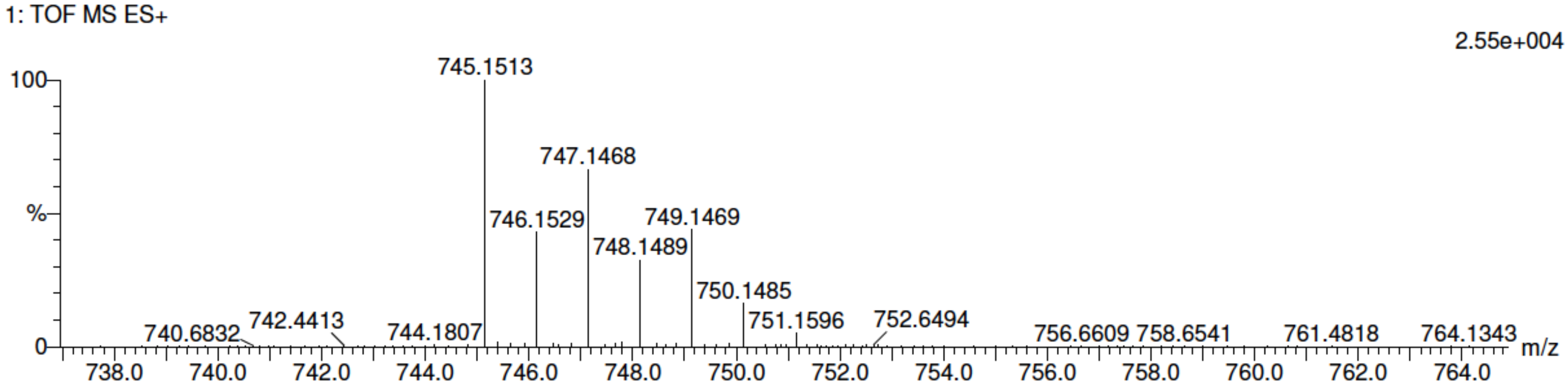

Minimum:				-1.5			
Maximum:		5.0	10.0	50.0			
Mass	Calc. Mass	mDa	PPM	DBE	i-FIT	i-FIT (Norm)	Formula
745.1513	745.1528	-1.5	-2.0	26.5	112.1	0.8	C41 H33 N2 O8 64Zn
	745.1461	5.2	7.0	23.5	111.9	0.6	C32 H29 N10 O8 64Zn

Figure S38. ^{1}H NMR spectrum of synthetic heterodimeric (clathridine A-clathridimine) Zn^{2+} (**10**) in $CDCl_3$ (500 MHz).

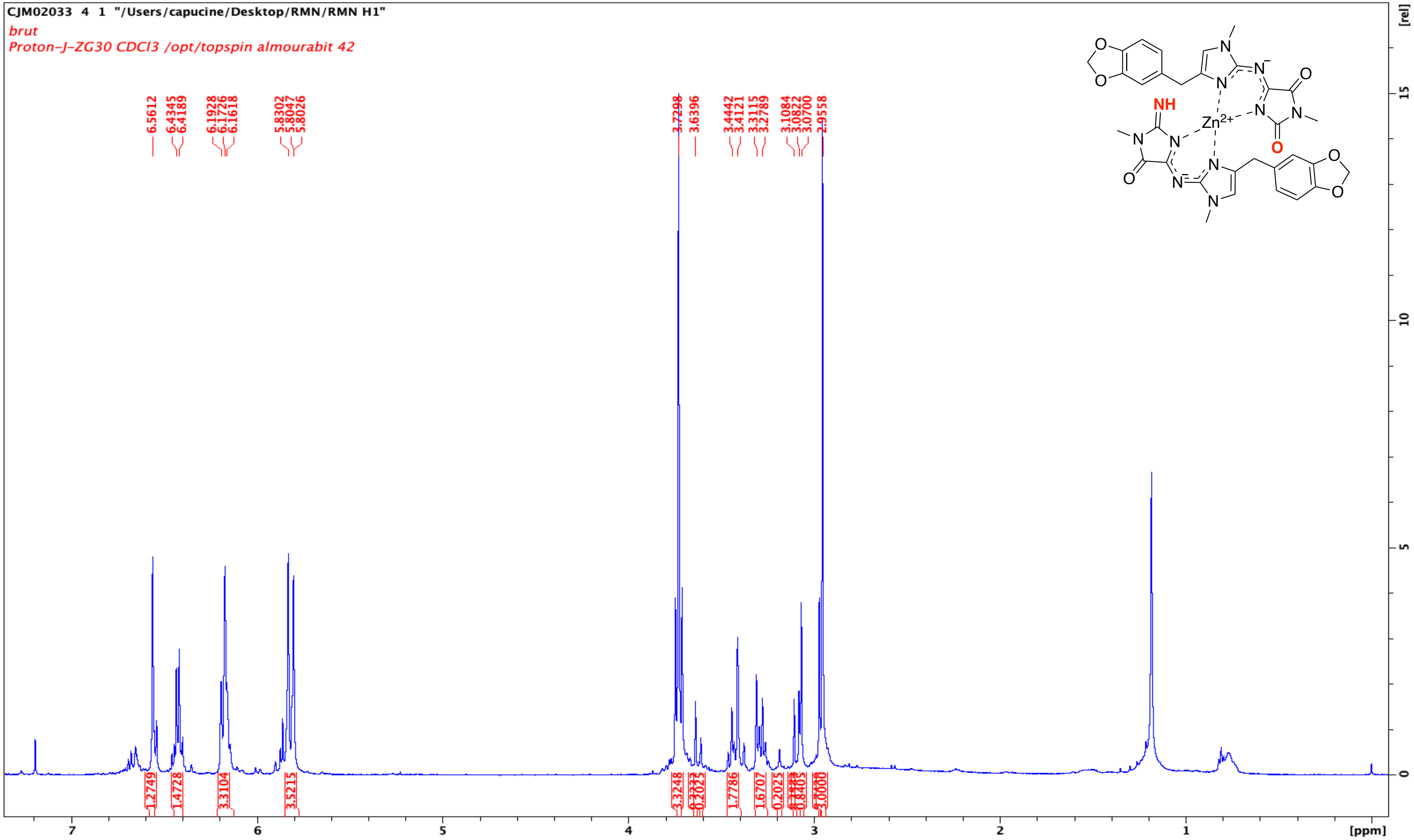

Figure S39. ^{13}C NMR spectrum of synthetic heterodimeric (clathridine A-clathridimine) Zn^{2+} (**10**) in $CDCl_3$ (125 MHz).

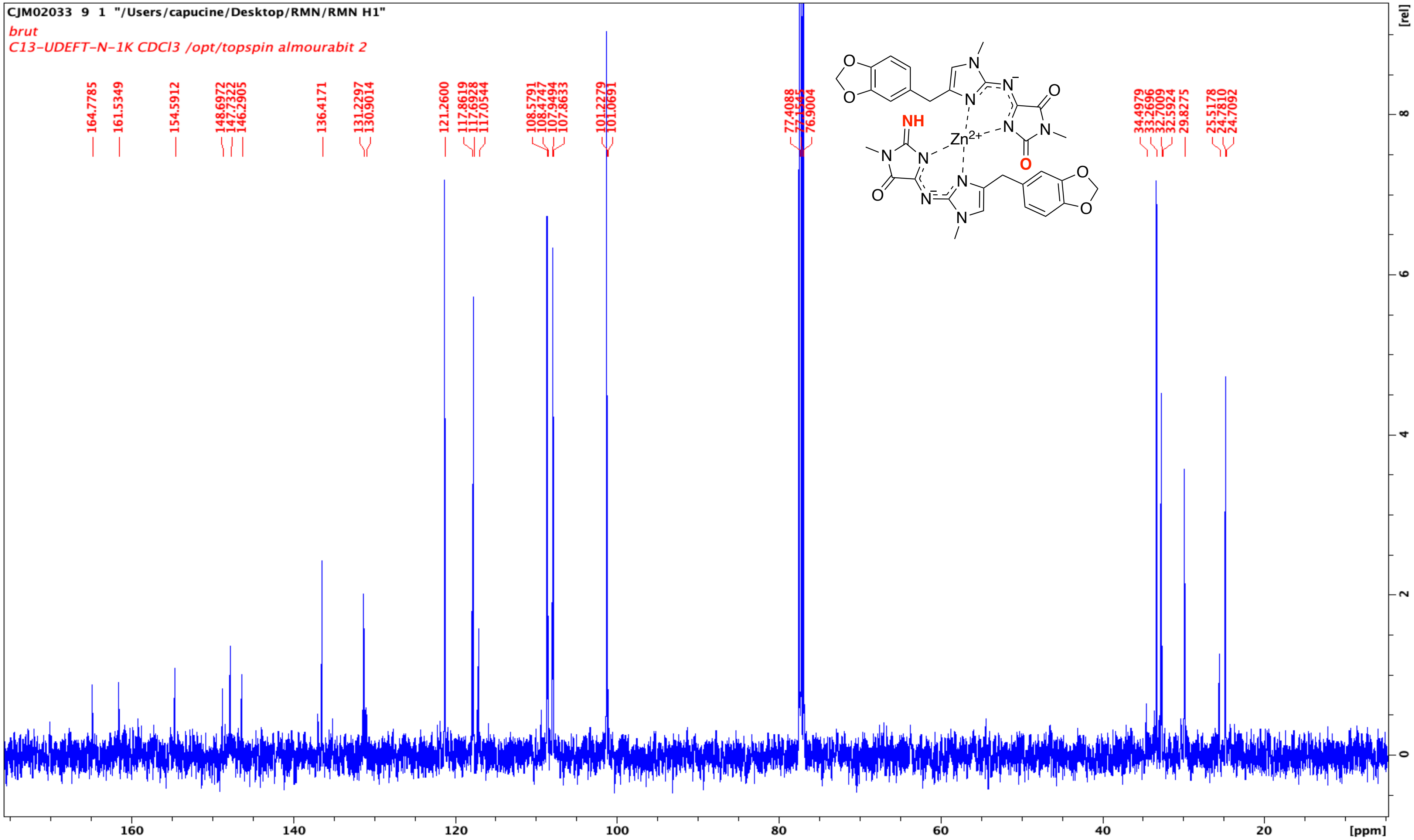

Figure S40. HR-ESI mass spectrum of the synthetic heterodimeric (clathridine A-clathridimine) Zn^{2+} (**10**).

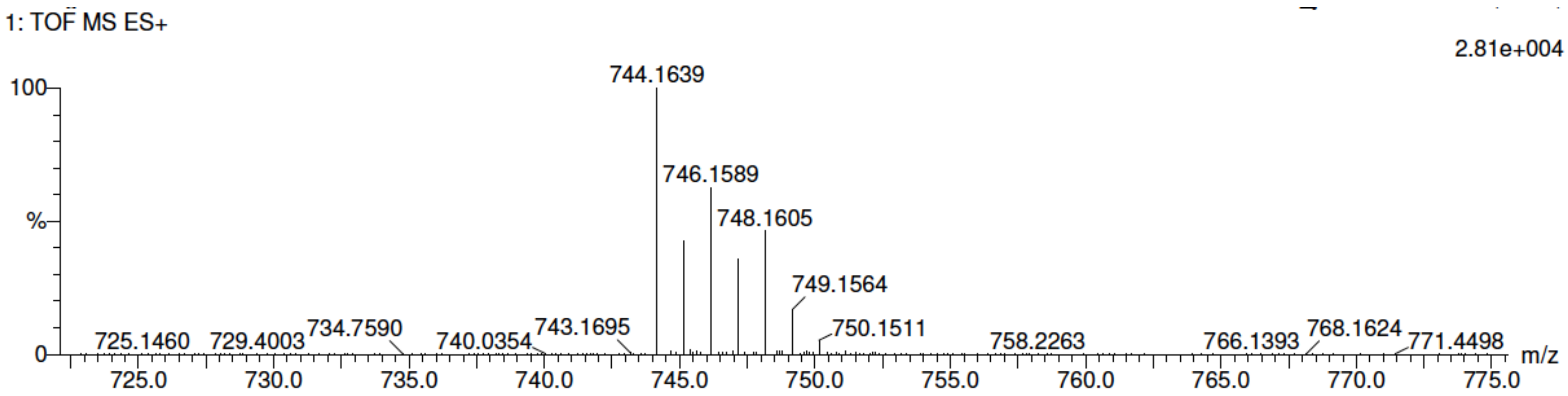

Minimum:				-1.5			
Maximum:		5.0	10.0	50.0			
Mass	Calc. Mass	mDa	PPM	DBE	i-FIT	i-FIT (Norm)	Formula
744.1639	744.1621	1.8	2.4	23.5	119.9	1.2	C32 H30 N11 O7 64Zn
	744.1576	6.3	8.5	26.5	120.0	1.3	C42 H34 N O8 64Zn
	744.1693	-5.4	-7.3	19.5	120.1	1.4	C26 H30 N15 O8 64Zn
	744.1688	-4.9	-6.6	26.5	120.2	1.5	C41 H34 N3 O7 64Zn

Figure S41. ^{1}H NMR spectrum of synthetic homodimeric (clathridimine)$_2$ Zn^{2+} (**27**) in $CDCl_3$ (500 MHz).

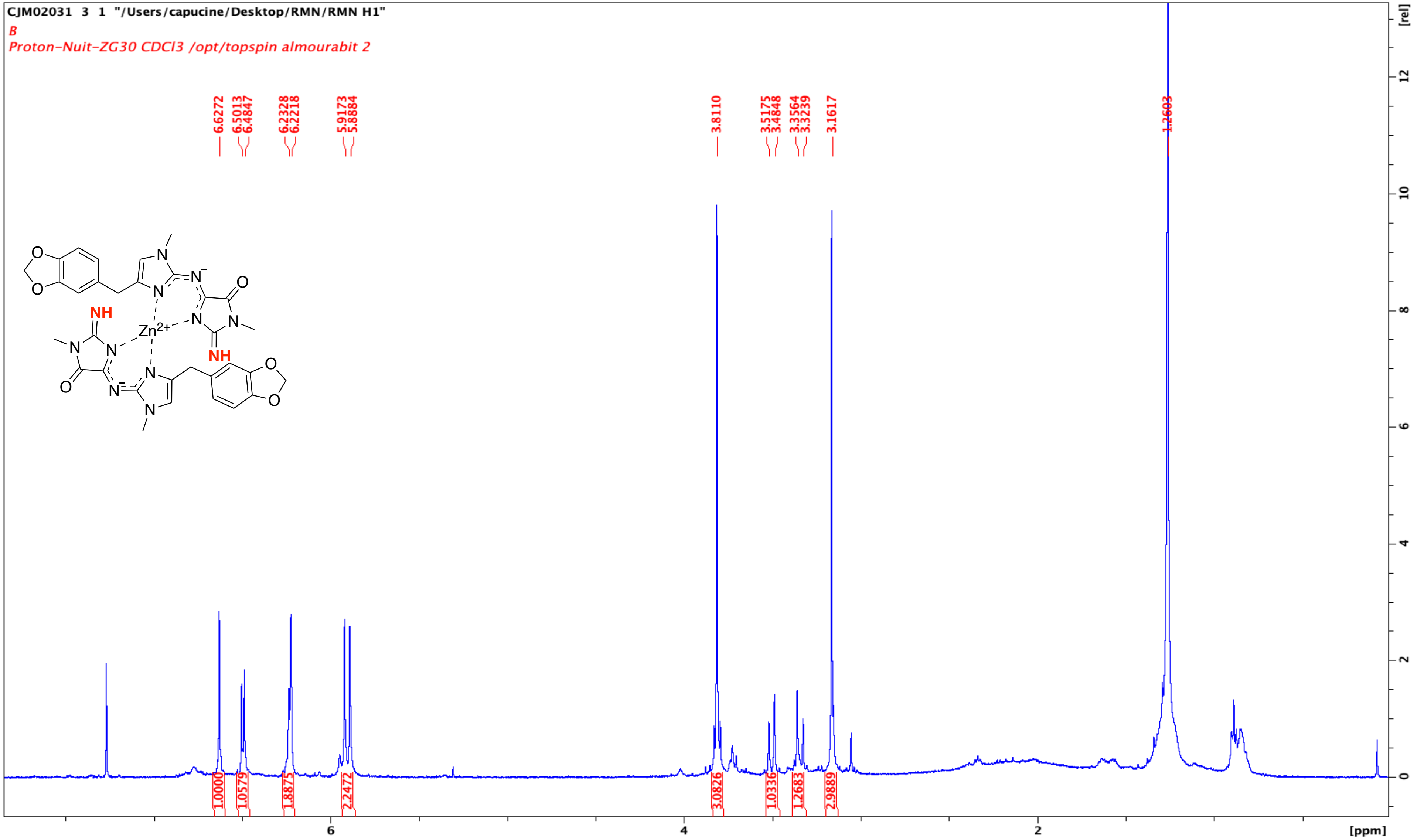

Figure S42. ^{13}C NMR spectrum of synthetic homodimeric (clathridimine)$_2$ Zn^{2+} (**27**) in $CDCl_3$ (125 MHz).

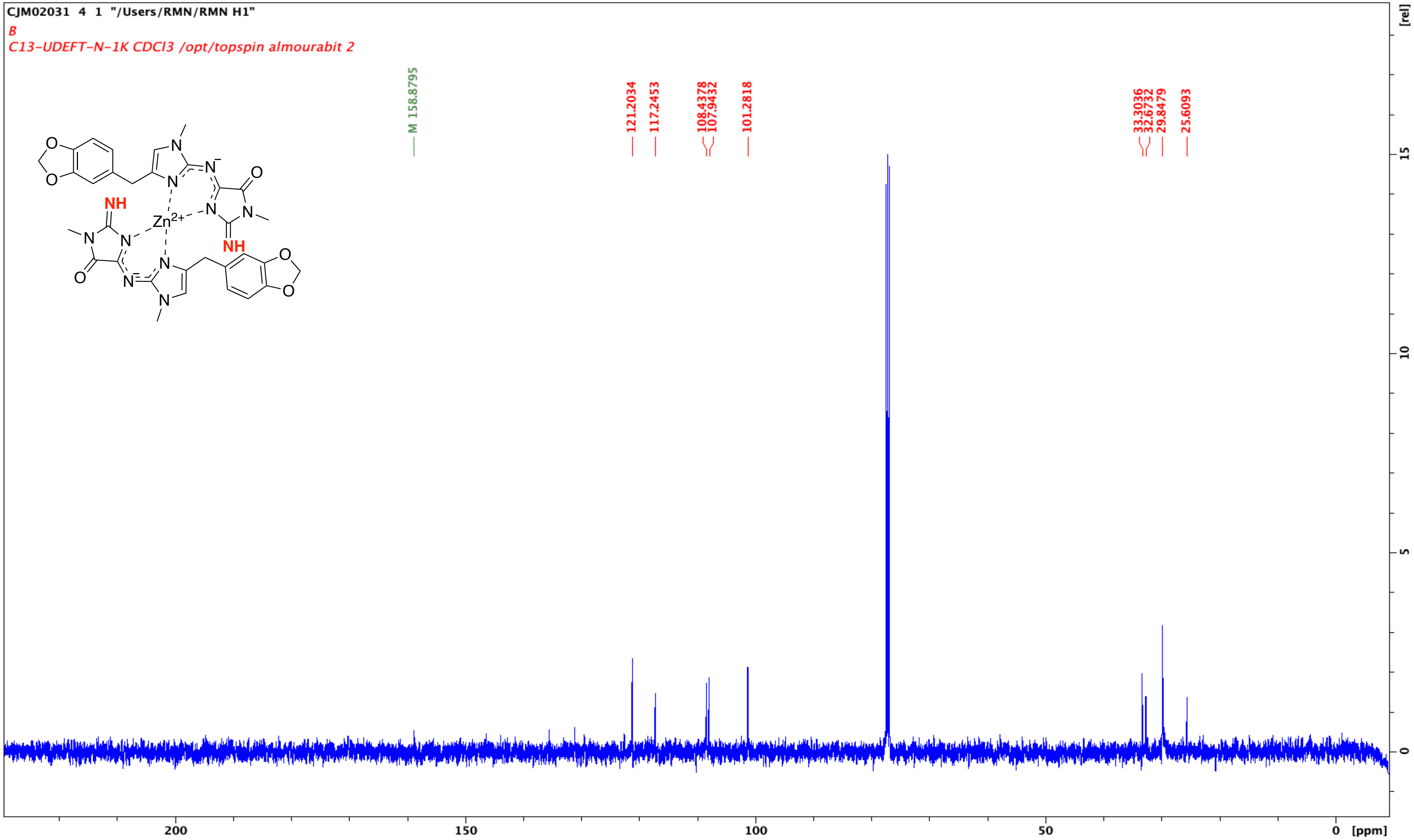

Figure S43. ^{1}H-^{13}C HMBC NMR spectrum of synthetic homodimeric (clathridimine)$_2$ Zn^{2+} (**27**) in $CDCl_3$ (125 MHz).

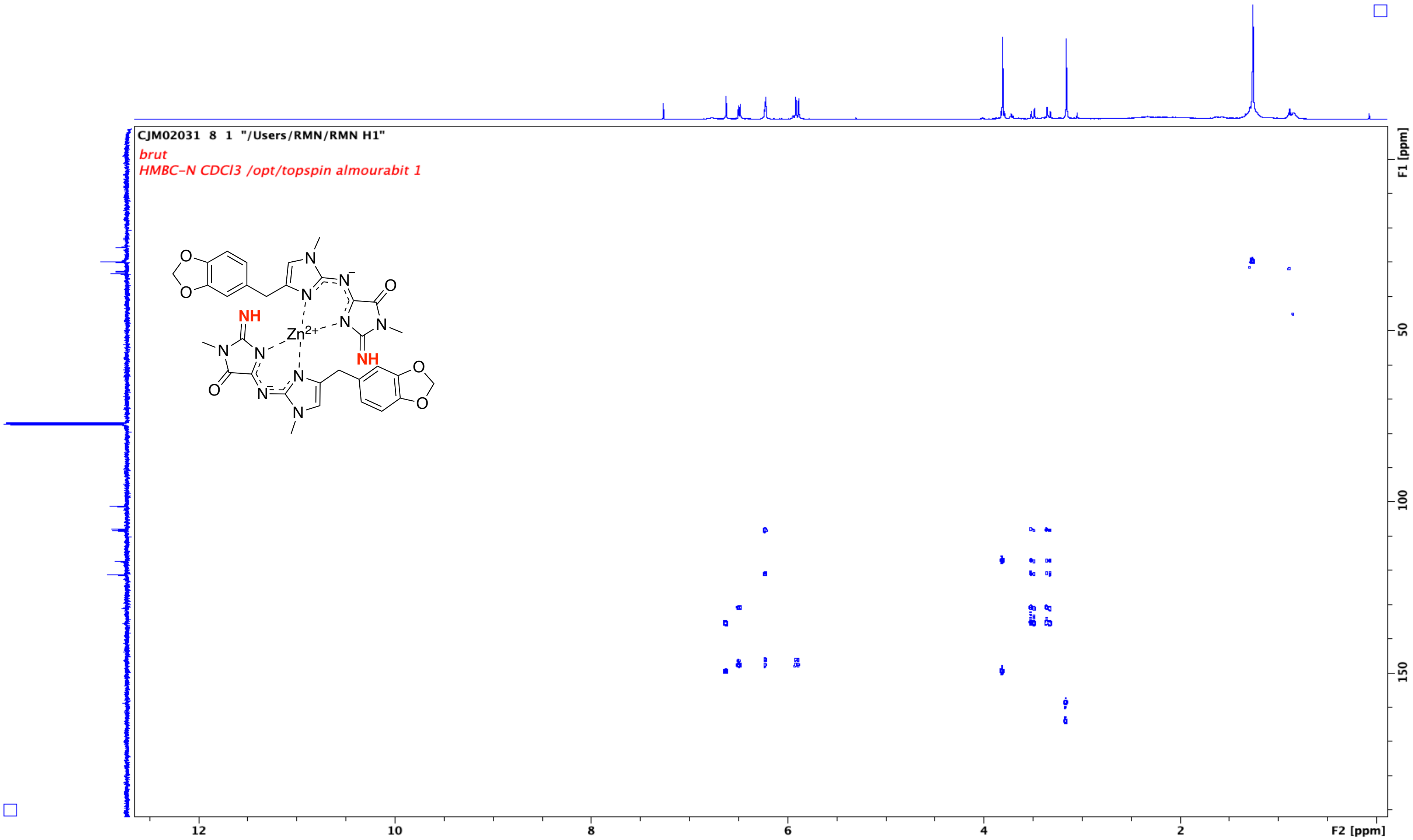

Figure S44. HR-ESI mass spectrum of the synthetic homodimeric (clathridimine)$_2$ Zn^{2+} (**27**).

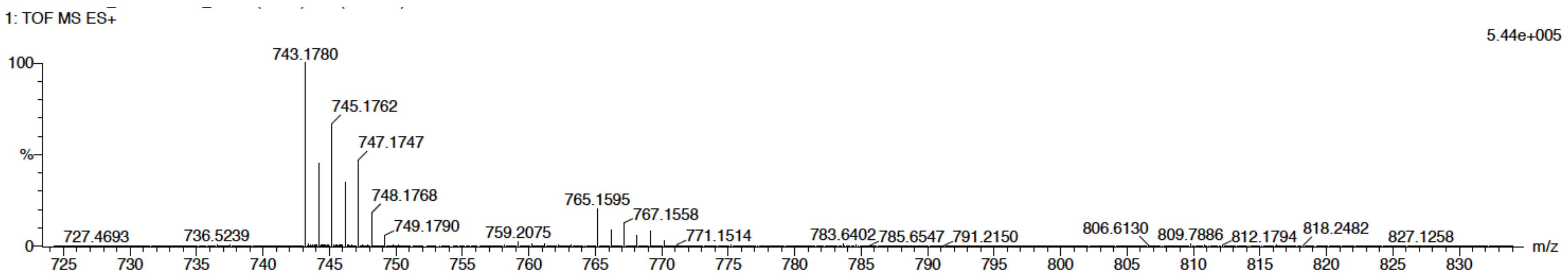

Minimum:				-1.5			
Maximum:		5.0	5.0	100.0			
Mass	Calc. Mass	mDa	PPM	DBE	i-FIT	i-FIT (Norm)	Formula
743.1780	743.1781	-0.1	-0.1	23.5	2112.7	0.4	C32 H31 N12 O6 Zn
	743.1768	1.2	1.6	18.5	2114.3	1.9	C31 H35 N8 O10 Zn
	743.1808	-2.8	-3.8	22.5	2114.4	2.0	C36 H35 N6 O8 Zn
	743.1749	3.1	4.2	31.5	2116.4	4.0	C43 H31 N6 O3 Zn
	743.1776	0.4	0.5	30.5	2118.5	6.2	C47 H35 O5 Zn
	743.1789	-0.9	-1.2	35.5	2118.5	6.2	C48 H31 N4 O Zn

Figure S45: LC-MS profiles of the synthetic mixture of complexes (blue) and the sponge crude extract (red), indicating the detection of the dimeric complexes including the minor heterodimeric complex **10**.

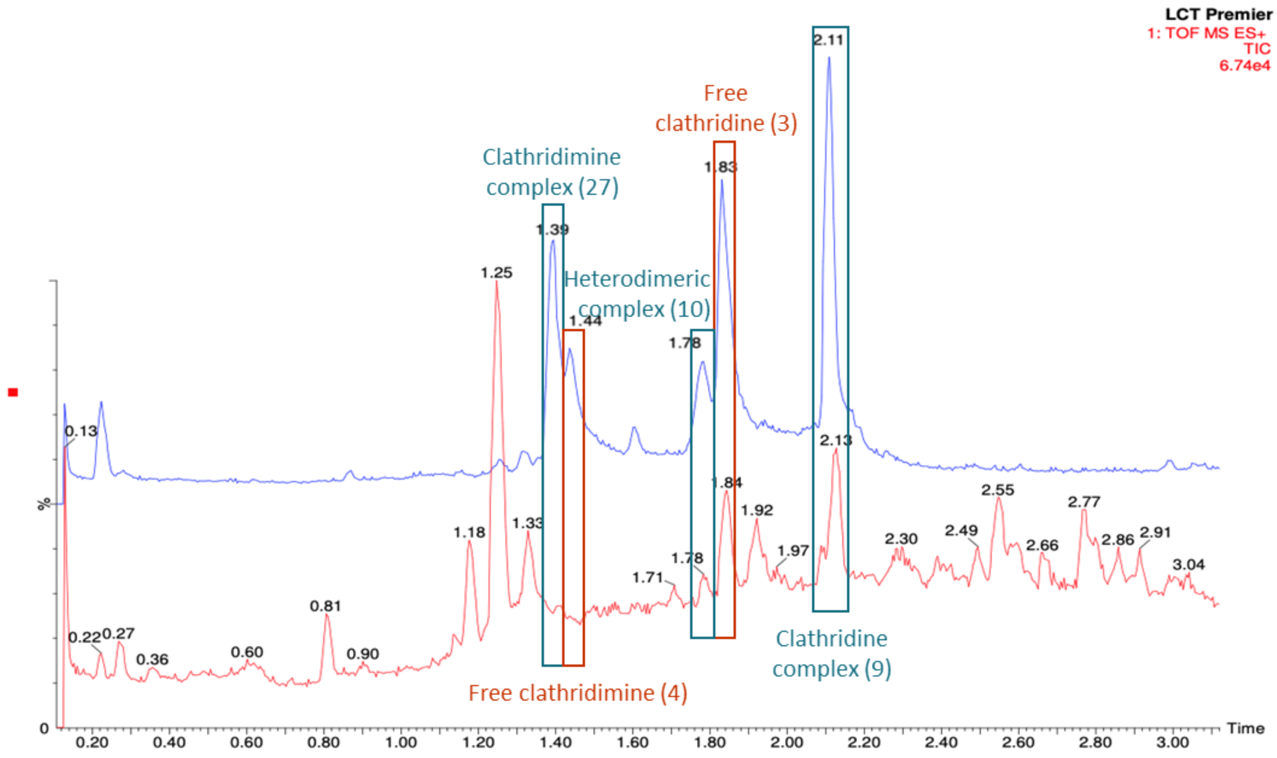

Figure S46: Superposition of the 1H NMR spectra of homodimeric (clathridine A)2Zn2+ (**9**) (green), homodimeric (clathridimine)2 Zn2+ (**27**) (red) and heterodimeric (clathridine A-clathridimine) Zn2+ (**10**) (blue).

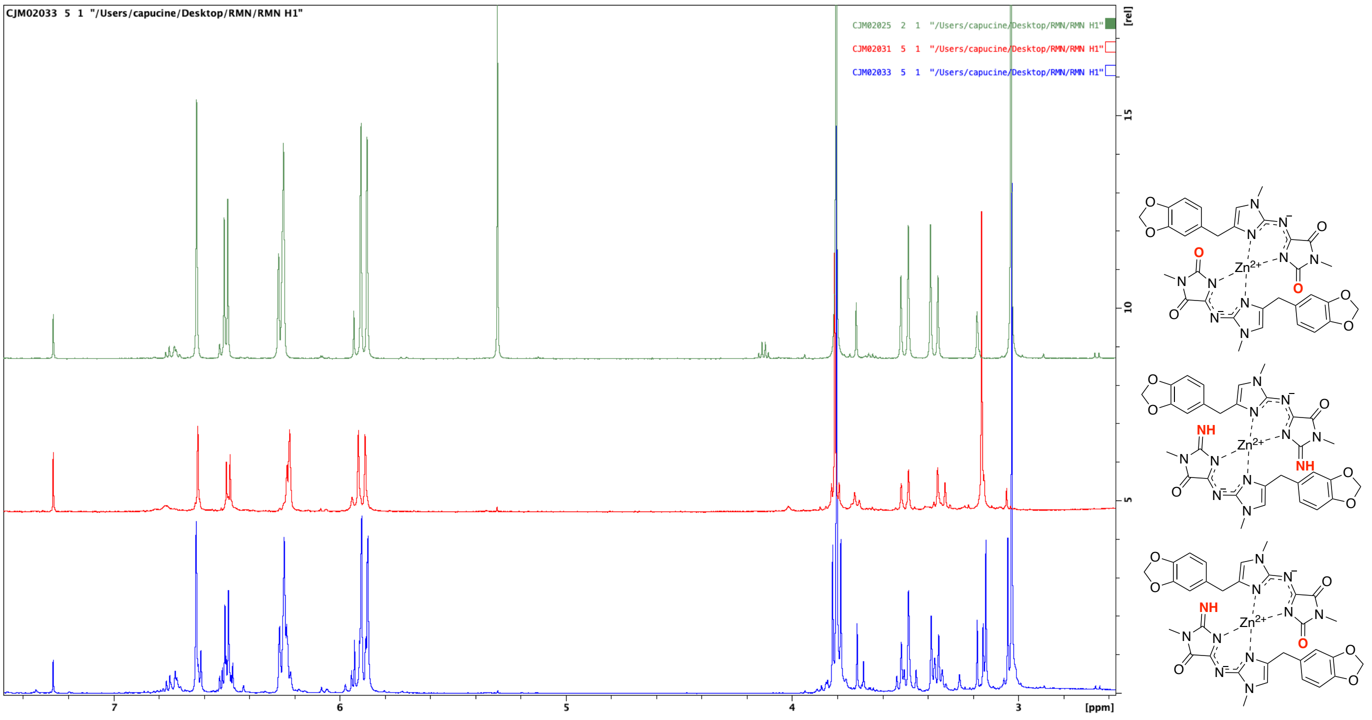

Figure S47: Zoom on the superposition of the 1H NMR spectra between 3 and 4 ppm, of homodimeric (clathridine A)2Zn2+ (**9**) (green), homodimeric (clathridimine)2 Zn2+ (**27**) (red) and heterodimeric (clathridine A-clathridimine) Zn2+ (**10**) (blue).

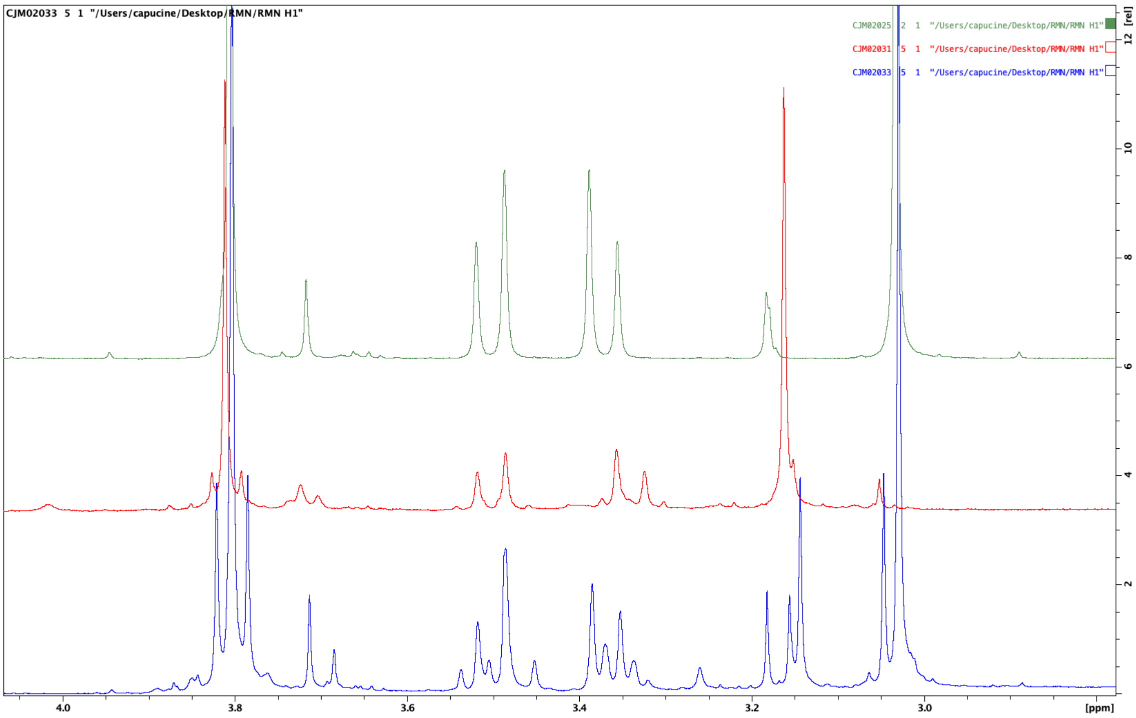

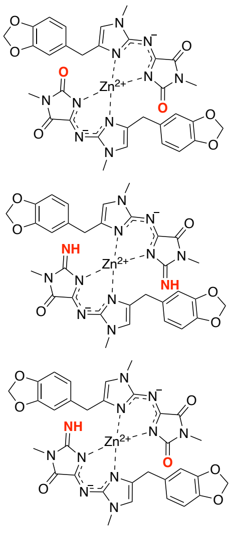

Single Crystal X-ray Crystallography (SC-XRD)

Crystal structure determination of homodimeric (clathridine A)$_2$ Zn^{2+} (9).

Crystal data for homodimeric (clathridine A)$_2$ Zn^{2+} (9)**:** $C_{32}H_{28}N_{10}O_8Zn[+1H_2O]$ (M =764.03 g/mol): triclinic, space group P-1 (no. 2), a = 9.4098(3) Å, b = 9.7775(3) Å, c = 21.1162(8) Å, α = 78.633(3)°, β = 87.210(3)°, γ = 63.813(3)°, V = 1707.55 (11) Å^3, Z = 2, T = 293.00 K, μ (Mo Kα) = 0.789 mm^{-1}, Dcalc = 1.486 g/cm^3, 32850 reflections measured (5.058° ≤ 2Θ ≤ 52.744°), 6964 unique (R_{int} = 0.0385, R_{sigma} = 0.0304) which were used in all calculations. The final R_1 was 0.0337 (I > 2σ(I)) and wR_2 was 0.0885 (all data).

Experimental

Crystals of compound **9** were obtained by slow evaporation of saturated DCM.

Single crystals suitable to X-ray diffraction structural analyses were transferred upon a microscope slide and one of them selected under a binocular, mounted on a nylon loop and fixed with Paratone® oil. Then, X-ray diffraction and crystallographic data were collected at room temperature using redundant ω scans on a Rigaku XtaLabPro single-crystal diffractometer using microfocus Mo Kα radiation and a HPAD PILATUS3 R 200K detector. CrysAlisPro 1.171.43.56a [1] was employed for the data processing, with SCALE3 ABSPACK scaling algorithm implemented for the empirical absorption correction using spherical harmonics and numerical absorption correction based on gaussian integration over a multifaceted crystal model.

Using Olex2 [2], the structures was readily solved by intrinsic phasing methods (SHELXT [3]), and by full-matrix least-squares methods on F^2 using SHELXL [4]. The non-hydrogen atoms were refined anisotropically, and hydrogen atoms, most of them were identified in difference maps and were treated as riding on their parent atoms.

Examination of this refined structure using the SQUEEZE procedure in PLATON [5] highlighted a void of volume of 177 Å^3 around the position 0 0 0.5 corresponding to crystallographic inversion centre. This void was filled by particularly disordered solvent whose contribution (calculated electron count of 17 electrons per void, *i.e. ca* one water molecule that could not be modelled properly) to the structure amplitudes was removed. Therefore, the likely half water molecule present in the asymmetric unit of the crystal was not included in the given chemical formula and other crystal data.

The molecular graphics presented here were computed with Mercury 2023.2.0 [6].

Crystallographic data have been deposited in the Cambridge Crystallographic Data Centre database (the deposition number is 2299450). Copies of the data can be obtained free of charge from the CCDC at www.ccdc.cam.ac.uk.

Citations

[1] Rigaku OD (2018). CrysAlis PRO. Rigaku Oxford Diffraction, Yarnton, Oxfordshire, England.
[2] Dolomanov, O.V., Bourhis, L.J., Gildea, R.J, Howard, J.A.K. & Puschmann, H. (2009), J. Appl. Cryst. 42, 339-341.
[3] Sheldrick, G.M. (2015). Acta Cryst. A71, 3-8.
[4] Sheldrick, G.M. (2015). Acta Cryst. C71, 3-8.
[5] Spek, A. L. (2015). Acta Cryst. C71, 9-18.
[6] Macrae, C. F., Edgington, P. R., McCabe, P., Pidcock, E., Shields, G. P., Taylor, R., Towler, M. & van de Streek, J. (2006). J. Appl. Cryst.39, 453-457.

Table 2: Crystal data and structure refinement

**Identification code**		**Compound 9**
**Empirical Formula**		$C_{32}H_{28}N_{10}O_8Zn$, 1[H_2O]
**Formula Weight**		764.03
**Crystal Color, Habit**		[light yellow, Prism]
**Crystal Dimensions (mm^3)**		0.24 × 0.2 × 0.05
**Crystal System**		triclinic
**Space Group**		*P-1*
**Unit cell dimensions**	*a* (Å)	9.4098(3)
	b (Å)	9.7775(3)
	c (Å)	21.1162(8)
	α (°)	78.633(3)
	β (°)	87.210(3)
	γ (°)	63.813(3)
**Volume (Å^3)**		1707.55(11)
**Z value**		2
**Calculated density $D_{calc.}$ (g.cm^{-3})**		1.486
**Absorption coefficient μ (mm^{-1})**		0.789
**F (000)**		788.0
**Diffractometer**		Rigaku XtaLAB PRO
**Radiation type**		Mo K_{α}
**Wavelength (Å)**		0.71073
**Voltage, Current (kV, mA)**		(50, 0.6)
***T* (K)**		293.00
**2θ range for data collection (°)**		5.058 to 52.744
**Limiting indices**		$-11 \leq h \leq 11$, $-11 \leq k \leq 12$, $-26 \leq l \leq 26$
**Reflections collected/unique**		32850/6964
**Completeness to θ full (%)**		99.8
**R_{int}**		0.0385
**Absorption correction**		Gaussian
**Refinement method**		Full-matrix least-squares on F^2
**Data/restraints/parameters**		6964/0/472
**Goodness-of-fit on F^2**		1.047
**Final R indices [$I>2\sigma(I)$]**	R_1	0.0337
	wR_2	0.0846
**R indices (all data)**	R_1	0.0424
	wR_2	0.0885
**Largest Δ peak and hole (e.Å^{-3})**		0.26/-0.23
**CCDC Deposit Number**		2299450

Figure S48: (left) ORTEP drawing of homodimeric (clathridine A)$_2$ Zn^{2+} (**9**) with thermal ellipsoids drawn at the 50% probability level; (right) Labelling scheme of the structure.

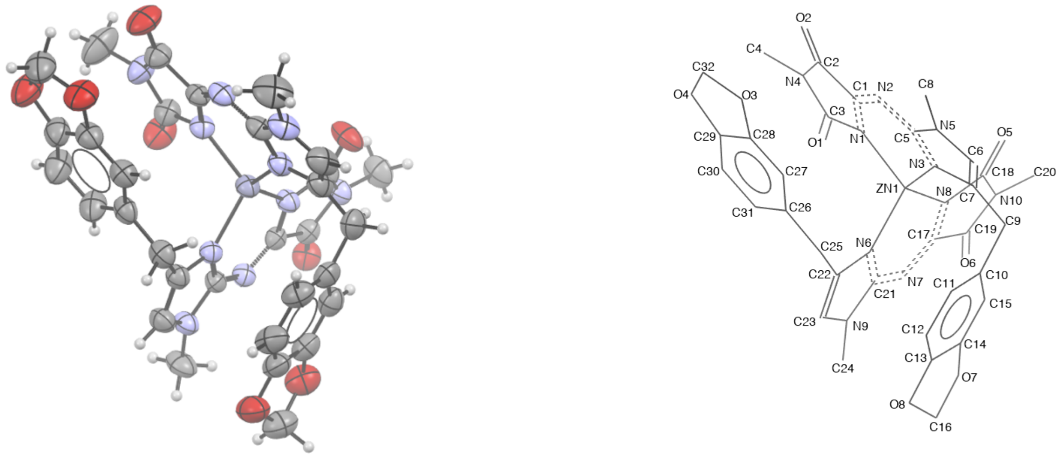

Figure S49: Energy diagram of intermediates involved in the hydrolysis reaction with one water molecule.

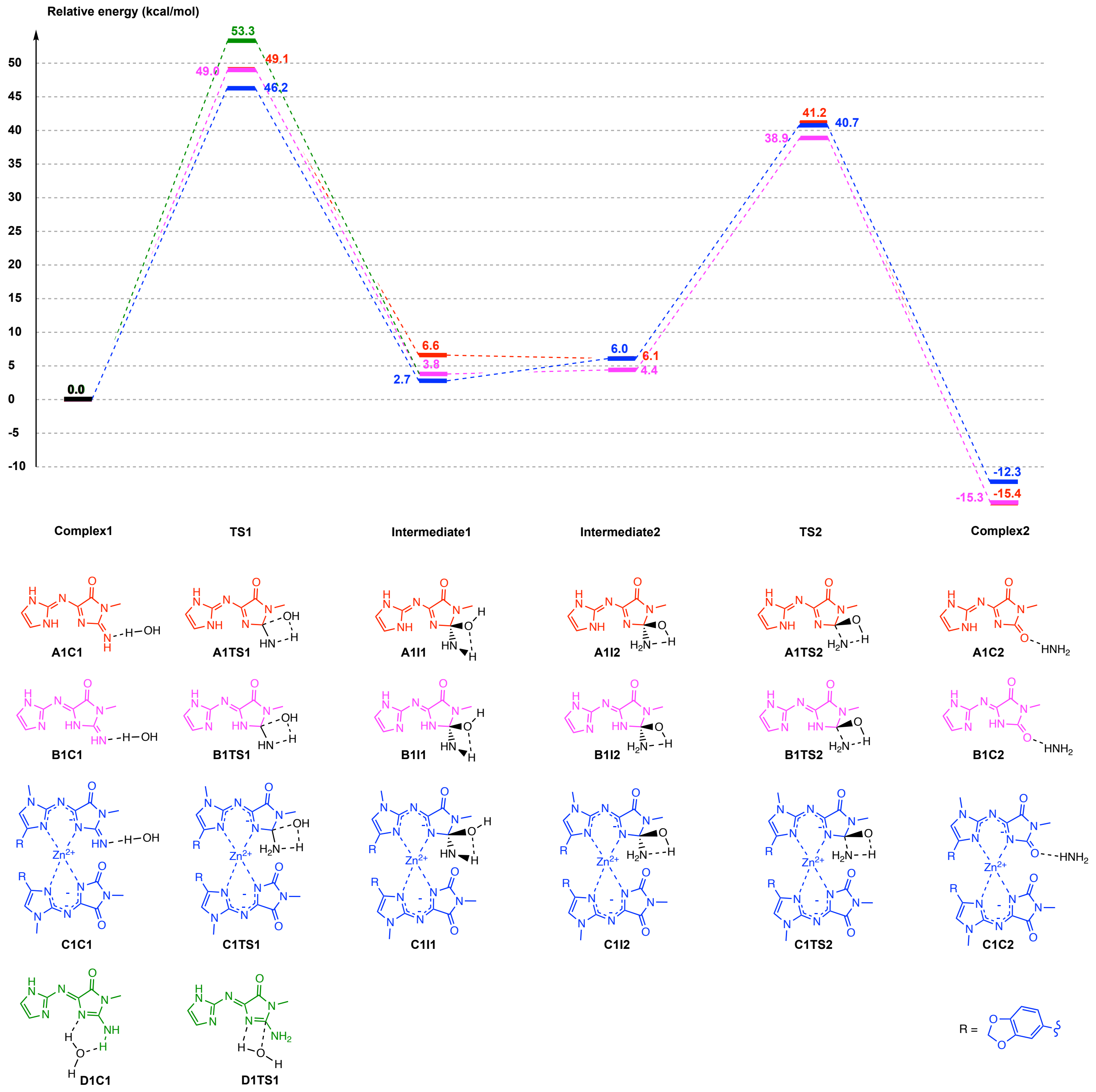

Figure S50: Energy diagram of intermediates involved in the hydrolysis reaction considering two water molecules.

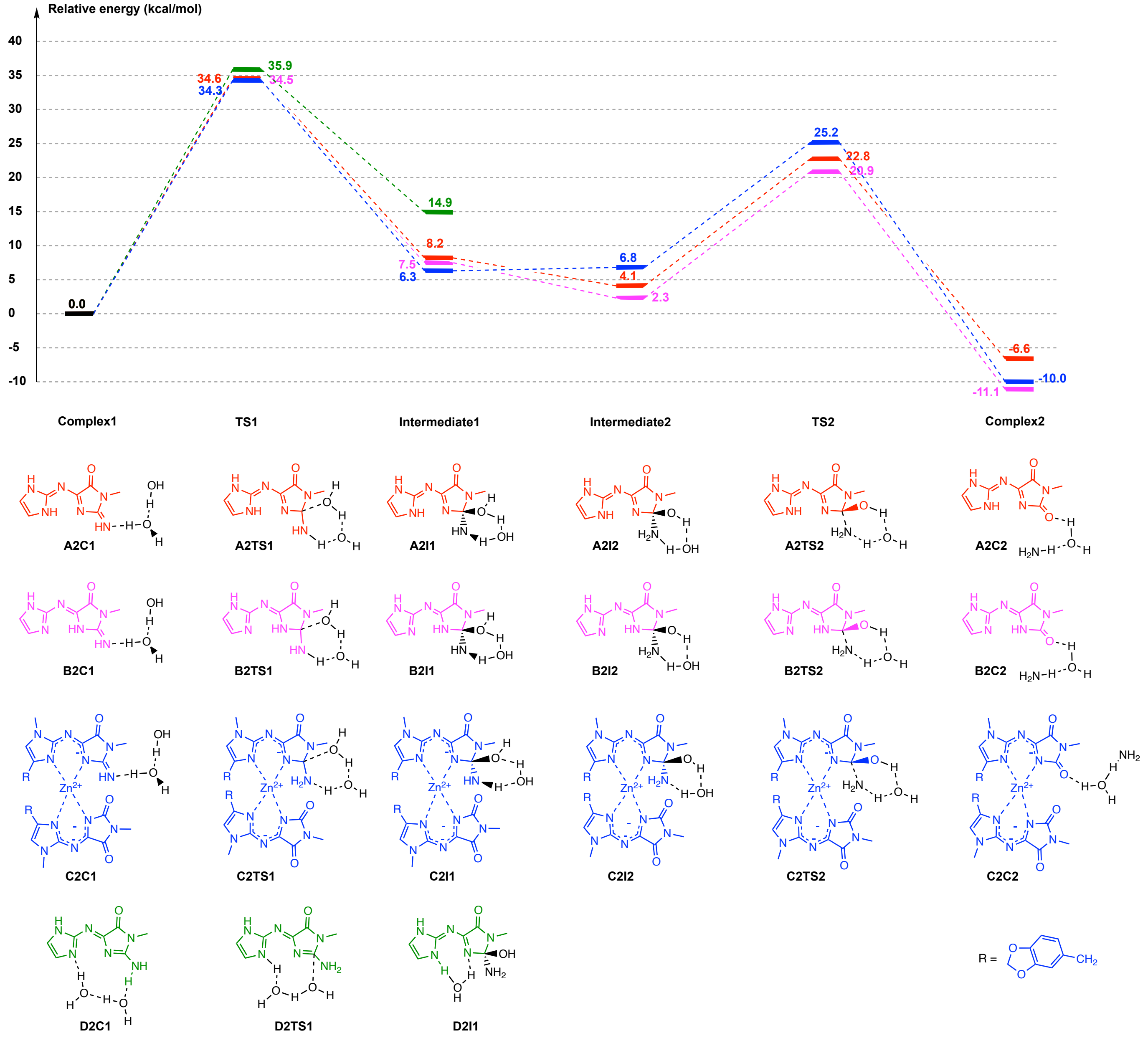

Figure S50: mineralization in comparison to Leucettamine B. Alizarin red nodules quantification of the ATDC5 cells micromass at Day 7, Day14 and Day21

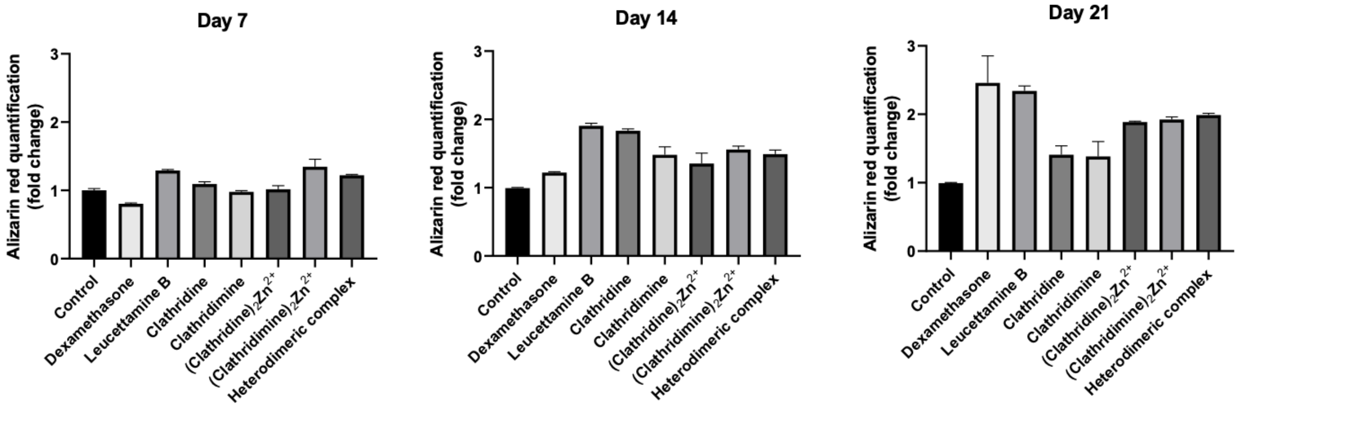

Supplement: Supplementary file 1 [file marinedrugs-22-00196-s001.zip › marinedrugs-2928238-supplementary.pdf]
